# Interaction of G-Protein βγ Complex with Chromatin Modulates GPCR-Dependent Gene Regulation

**DOI:** 10.1371/journal.pone.0052689

**Published:** 2013-01-09

**Authors:** Anushree Bhatnagar, Hamiyet Unal, Rajaganapathi Jagannathan, Suma Kaveti, Zhong-Hui Duan, Sandro Yong, Amit Vasanji, Michael Kinter, Russell Desnoyer, Sadashiva S. Karnik

**Affiliations:** 1 Department of Molecular Cardiology, Lerner Research Institute, Cleveland Clinic, Cleveland, Ohio, United States of America; 2 Department of Cell Biology, Lerner Research Institute, Cleveland Clinic, Cleveland, Ohio, United States of America; 3 Biomedical Imaging and Analysis Core, Lerner Research Institute, Cleveland Clinic, Cleveland, Ohio, United States of America; 4 Department of Computer Science, University of Akron, Akron, Ohio, United States of America; University of Massachusetts Medical, United States of America

## Abstract

Heterotrimeric G-protein signal transduction initiated by G-protein-coupled receptors (GPCRs) in the plasma membrane is thought to propagate through protein-protein interactions of subunits, Gα and Gβγ in the cytosol. In this study, we show novel nuclear functions of Gβγ through demonstrating interaction of Gβ_2_ with integral components of chromatin and effects of Gβ_2_ depletion on global gene expression. Agonist activation of several GPCRs including the angiotensin II type 1 receptor specifically augmented Gβ_2_ levels in the nucleus and Gβ_2_ interacted with specific nucleosome core histones and transcriptional modulators. Depletion of Gβ_2_ repressed the basal and angiotensin II-dependent transcriptional activities of myocyte enhancer factor 2. Gβ_2_ interacted with a sequence motif that was present in several transcription factors, whose genome-wide binding accounted for the Gβ_2_-dependent regulation of approximately 2% genes. These findings suggest a wide-ranging mechanism by which direct interaction of Gβγ with specific chromatin bound transcription factors regulates functional gene networks in response to GPCR activation in cells.

## Introduction

The Gβ and Gγ subunits form a functionally inseparable Gβγ complex that generate the quiescent heterotrimeric G-proteins by associating with Gα-GDP. Current models show that G-protein activation by G-protein coupled receptors (GPCRs) occur at the plasma membrane (PM). Second messengers or protein–protein interactions leading to spatio-temporal propagation of signals initiated by Gα and Gβγ to the nucleus occurs in the cytoplasm, however translocation of G-protein subunits to nucleus is not frequently considered a possibility [1]. This view is changing due to the discovery of the shuttling of Gα and Gβγ subunits from the PM to cell organelles, such as the Golgi, mitochondria, endosomes, and occasionally, the nucleus [2], [3]. It is possible therefore, that Gα or Gβγ complex translocates to nucleus and participate in gene regulation.

Gene regulation through G-protein signaling is crucial to human adaptation and survival which reflects the enormous success of therapeutics targeting GPCRs, the largest family of receptors encoded by the human genome. The finely tuned expression of an appropriate set of genes in a cell depends on multiple transcription factors (TFs) and transcriptional co-activators. GPCRs enhance gene transcription by facilitating the interaction of histone acetyl transferases (HATs), such as p300/CBP, to TFs on chromatin [4]. Alternatively, recruitment of histone deacetylases (HDACs) to chromatin-bound TFs, such as myocyte enhancer factor 2A (MEF2A), represses transcription, and the repression is relieved by GPCR signals [5]. Nuclear localization of β-arrestins [6], GRK5 [7] and RGS proteins [8] is reported which suggests that these proteins recruited into the nucleus upon ligand activation of GPCRs may participate in the epigenetic processes that are essential for the functioning of cells. Whether Gα or Gβγ which are the primary transducers of GPCR signals, regularly enter the nucleus and directly participate in GPCR-coordinated transcriptional response remains unclear. Reports of Gβ_1_ or Gβ_2_ association with the glucocorticoid receptor [9], Gβ_1_γ_2_ association with HDAC5 [10], [11], Gβ_5_ association with the nuclear shuttling of the R7 family of RGS proteins [8] and Gβγ_5_ association with the adipocyte enhancer binding protein [12] suggest a potential broad role of Gβγ in gene regulation. Therefore, we hypothesized that agonist activation of a typical GPCR such as the angiotensin II type 1 receptor (AT_1_R), changes the composition of chromatin-associated proteins which may include changes in the levels of specific G-protein subunits.

An unbiased high-throughput mass spectrometry analysis of the nuclear proteome upon activation of a GPCR led us to discover the interactions of Gβ_2_γ_12_ with chromatin. We found that the level of Gβ_2_ increased in the nucleus upon activation of diverse GPCRs and that Gβ_2_ was essential for agonist-induced MEF2A function. Gβ_2_ interacted with a sequence motif present in several TFs, and this interaction accounted for the coordinated gene regulatory function of Gβγ.

## Materials and Methods

### Reagents

The following reagents were used: HEK-293 cells (American Type Culture Collection) and NRVMs (Lonza); the pBudE4.1 plasmid, hygromycin and FuGENE 6™ (Invitrogen); geneticin (Gibco); Benzonase™ (Novagen); the agonists 5-hydroxytryptamine (5-HT), dobutamine (DOB), and isoproternol (ISO); and anti-skeletal-actinin, anti-myc, and anti-FLAG antibodies, and anti-FLAG-M2 agarose beads (Sigma); antibodies against STAT1, STAT3, H2A, H2B, H4, MEF2A, TAF, Gαq, pan Gβ, Gβ_2_, NFAT, GATA4 and α-actinin-1 (Santa Cruz Laboratories); TBP (Abcam) and phospho- and total HDAC5 antibodies (Genscript); an anti-HA antibody (Zymed Laboratories); an α-actinin-4 specific antibody (Immunoglobe); an amino-terminal FLAG-tagged human Gβ_2_ plasmid and a myc-tagged human Gγ12 (UMR) plasmid; and an α-actinin-4 plasmid (Origene).

### Nuclear and cytosolic fractionation

The nucleus and cytosol were isolated using the NUC101 nuclei isolation kit as detailed by the manufacturer (Sigma-Aldrich). The nuclear fractions were stained with DAPI, and subsequent visualization was performed using confocal microscopy to check for the integrity of nuclei. Nuclear protein was extracted using Benzonase (10 units/ml at 37°C for 60 min), which digests the nucleic acids without denaturing the proteins (chromatin proteins). The pellet was further extracted to isolate the tightly bound proteins using 0.45 N sulfuric acid (acid fractions). The purity of the fractions was determined by immunoblotting for specific cellular compartment markers, histones H1 and H2A (nuclear) and Gqα (cytosolic).

### Site directed mutagenesis and plasmid construction

The amino terminus HA-tagged rat AT_1_R [13] under the control of the human cytomegalovirus (CMV) promoter was generated in the pcDNA3 plasmid. FLAG-Gβ_2_ was subcloned into pBudE4.1 under the EF1 promoter. Nested primers were designed to delete each of the seven WD repeats in the FLAG-Gβ_2_ construct. All of the subcloned plasmid constructs and the WD40 mutants were verified by DNA sequencing.

### Transfection and generation of AT_1_R -expressing stable hek-293 cells

Routine transient transfection of HEK-293 cells was performed with FUGENE 6™ per the manufacturer's recommendations. The cell line stably expressing HA-AT_1_R was established by clonal isolation using geneticin (600 µg/ml) selection.

### LC-tandem mass spectrometry and protein identification

For Gel C analyses [14], the gels are run to attain 50% resolution of the electrophoresed proteins. The gels were cut into three regions, and each of these regions was further cut into five equal parts and digested with trypsin. The peptides were extracted and concentrated, and the digest was analyzed by LC-tandem MS [15]. The proteins contained in the nuclear fractions were identified using a shotgun sequencing approach [16]. Relative quantitation was determined using a spectrum counting approach. The MS results were also examined by plotting mass chromatograms for the respective peptides. Data were also searched using SEQUEST (ThermoFisher, San Jose, CA) with mass tolerances set at 3.0 Da for peptides and 2.0 for fragment ions using the standard variable oxidation of methionine (+16 Da) and carbamidomethylation on cysteine (+57 Da) as fixed modification. The MASCOT program (www.matrixscience.com) was used to compare all of the CID spectra with the NCBI non-redundant database and to identify the protein. Matching peptides were verified by manual interpretation.

### Isolation of ventricular myocytes from adult C57BL6 mice

The isolation procedure for ventricular cardiac myocytes from adult C57BL6 mice has been reported in detail [17]. Handling of animals used for myocyte preparation is approved by IACUC using standard protocol recommendation. Cardiac myocytes were enzymatically dispersed via Langendorff perfusion of mouse hearts [18]. Following isolation, cells were treated with 1 µM AngII for 30 min and then fixed with 3% paraformaldehyde and subjected to immunocytochemical analysis.

### Measurements of [Ca^2+^] flux

Single ventricular myocytes were incubated with 1 mM fura-2 acetoxymethyl ester (Molecular Probes) for 10 min at room temperature in the dark. [Ca^2+^]_i_ signaling was measured using a dual excitation spectrofluorometer (Deltascan RFK6002, Photon Technology International) at excitation wavelengths of 340 and 380 nm and an emission wavelength of 510 nm as previously described [19]. Steady-state [Ca^2+^]_i_ transient signals were recorded at a pacing frequency of 0.5 Hz in the absence of AngII. The stimulation was stopped and then followed by 1 µM AngII treatment. The resulting AngII-induced [Ca^2+^]_i_ signal was recorded as a qualitative index of the initial sarcoplasmic reticulum (SR) [Ca^2+^]_i_ load in the cardiomyocytes.

### Immunocytochemistry and confocal microscopy

Immunolocalization using confocal microscopy were performed essentially as described previously [20]. For 3D-visualization of confocal image stacks, the confocal image slices of myocytes labeled with DAPI and FITC (0.13 mm×0.13 mm×0.04 mm resolution in X, Y, and Z directions, respectively) were imported into Image-Pro 6.1 (Media Cybernetics, Silver Springs, MD) as a multi-plane sequence and subsequently split into blue and green channels. Customized sequence segmentation scripts were then applied to the blue (DAPI) channel to threshold and binarize each slice. The binarized image stack was then multiplied with the green channel stack (plane-by-plane) to extract green fluorescence localized to the nucleus. Both binarized nuclear slices and their corresponding green fluorescent slices were exported into MicroView (GE Healthcare, Piscataway, NJ), reconstructed into 3D volumes (Z-dimension resolution was increased five times to improve definition of flattened nuclei), and rendered as iso-surfaces. Lastly, these iso-surfaces (DAPI and FITC) were merged together, and the opacity of the nucleus was adjusted to allow visualization of the underlying protein.

### Reporter assays

The MEF2-luciferase assay (Promega) was performed as recommended by the manufacturer. Briefly, the MEF2-luciferase plasmid (1 µg) was transfected into AT_1_R-expressing cells in the presence or absence of Gβ_2_ to evaluate the role of Gβ_2_ in modulating MEF2A activity. The WD40 repeats of the N-terminal FLAG-tagged Gβ_2_ (FLAG-Gβ_2_) construct were sequentially deleted to create FLAG-Gβ_2_ ΔWD(1–7) mutants. The HEK-AT_1_R cells were then transfected with FLAG-Gβ_2_ ΔWD mutants, the MEF2-luciferase plasmid and 0.15 µg of the βGal plasmid (transfection control).

### Immunoprecipitations with FLAG- Gβ_2_ and WD40 repeat mutants

The role of WD40 repeats in Gβ_2_ involved in the interaction with MEF2A was performed and quantified (Kodak Imager ID 3.6) as previously described [21].

### Deacetylase assays

The HDAC activity (UPSTATE) was performed per the manufacturer's guidelines. For deacetylase assays, the nuclear and cytosolic fractions were obtained from AT_1_R and AT_1_R-Gβ2i cells (+/−30 min of 1 µM AngII). Actinin (1 µg anti-actinin-1 antibody)-associated deacetylase activity was measured in 100 µg each of the cytosolic and nuclear fractions. The samples were incubated with gentle mixing at 4°C overnight. Immunoprecipitates were collected via centrifugation and washed twice with 1 ml of ice-cold phosphate buffered saline (PBS). The resin was assayed for deacetylase activity.

### RNAi-mediated knockdown of Gβ_2_

A DNA vector-based siRNA was designed to stably knockdown the expression of Gβ_2_ (gene name: GNB2) in HEK-293 cells [21]. The target sequence, ACTGGGTACCTGTCGTGTT [21], and the scrambled sequence, CGGTGTTCTACGTGGCTAT, were cloned into the pRNATU6.1/hygro plasmid under the control of the U6 promoter and a hygromycin selection marker. The cells were also co-transfected with the HA-AT_1_R-expressing plasmid that contained a neomycin selection marker. Selected clones were maintained in media containing hygromycin (100 µg/ml) and geneticin (600 µg/ml).

### Microarray analysis

HEK-293 (AT_1_R; −/+AngII and AT_1_R-Gβ_2_i; −/+AngII) cells were harvested under RNase-free conditions, and RNA was isolated using the RNeasy kit (Qiagen). RNA-based probe synthesis and hybridization were performed by the Gene Expression Array Core Facility at Case Western Reserve University (www.geacf.net) as described previously [21] using high-density oligonucleotide HG-U133 Plus 2 arrays (Affymetrix, Inc.; Santa Clara, CA). Gene expression changes predicted in the Gβ2i cells for proteins examined in this study were validated by western blot analysis of Gβ2i cell lysates.

Functional and network analyses of gene expression data were performed using Ingenuity software (Ingenuity Systems, http://www.ingenuity.com). The score assigned to any given gene network takes into account the total number of molecules in the data set, the size of the network, and the number of “network eligible” genes/molecules in the data set. The network score is based on the hypergeometric distribution and is calculated with the right-tailed Fisher's exact test. The network score is the negative log of this P-value. To identify the genes regulated by the 4 key TFs, MEF2A, NFAT, STAT1, and STAT3, we used the ‘Build Networks – Expand by one group interaction’ algorithm in MetaCore™ (www.genego.com). Only transcriptional regulation interaction was considered for this analysis, and the list of 658 genes (Gβ2-regulated genes) was overlaid onto these networks. Only those genes that were transcriptionally regulated by one or more of these TFs as well as differentially regulated in our datasets are shown in the figure.

### Molecular modeling

Reference 3D structures of Gβ1γ2 [PDB: 1GP2] were retrieved from the Protein Data Bank (PDB) (http://www.rcsb.org/pdb/home/home.do) through the NCBI website (http://www.ncbi.nlm.nih.gov). For homology modeling, target and template sequences were aligned using CLUSTAL X. The alignment was then submitted electronically to the Swiss Model server (http://www.expasy.org), which generates the homology model based on the template structure. Energy computations were performed in vacuo using the GROMOS96 implementation of the Swiss PDB Viewer (SPDBV) program (Swiss Institute of Bioinformatics). Energy minimization was carried out by 20 cycles of steepest descent, and minimization was stopped when the Δ energy was below 0.05 kJ/mol, as previously described. Hydrogens were added using VEGA ZZ (University of Milan, Italy; (http://www.ddl.unimi.it/vega/index2.htm). The model was then submitted for Ramachandran analysis. The structures were visualized using the PYMOL program (The PyMOL Molecular Graphics System, DeLano Scientific, Palo Alto, 2002).

### Statistical analysis

All experiments were performed three or more times. For image analysis, approximately 50–100 cells were analyzed in each set, and representative images are shown. All data are expressed as the mean ± SEM of at least three independent experiments. Each experiment was performed in triplicate unless otherwise indicated. Data were analyzed using an unpaired Student's t-test (*P*<0.05) using GraphPad Prism 4 software.

## Results

### Gβ_2_ and Gγ_12_ traffic into the nucleus upon GPCR activation

**Figure 1:**
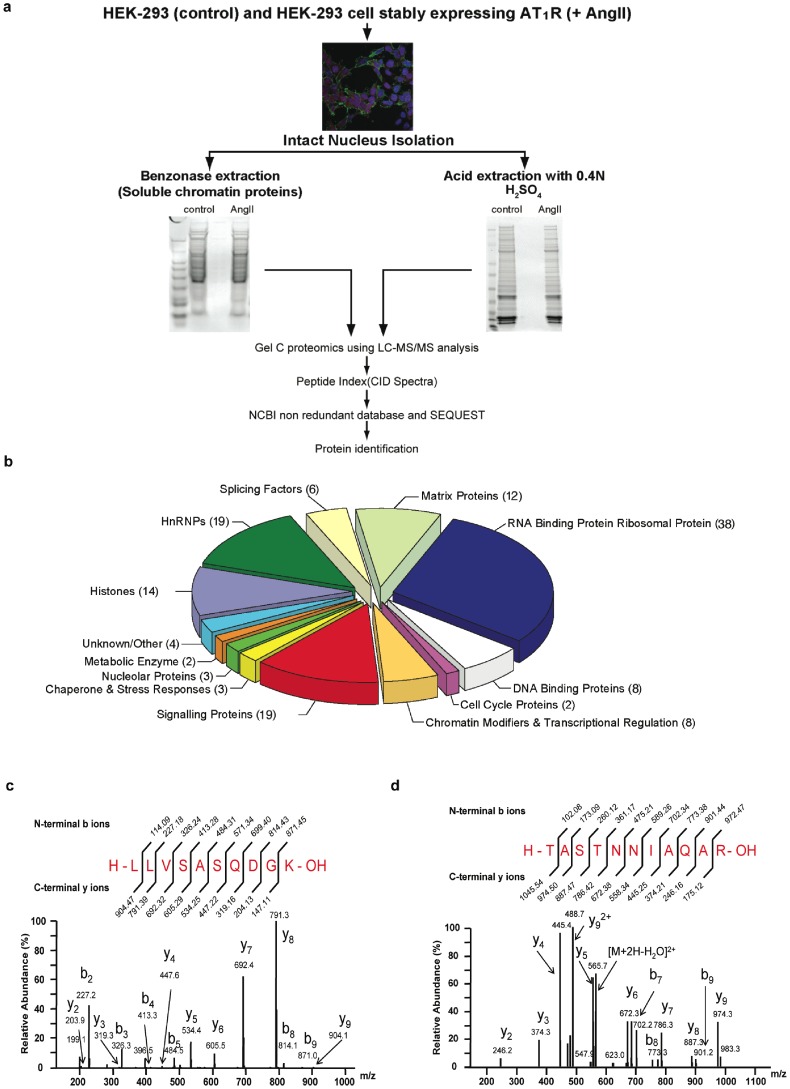
G-protein β_2_ and γ_12_ subunits are components of the nuclear proteome. (a) Schematic for the isolation of intact nuclei from the cytosol and characterization of the nuclear proteome by mass spectrometry analysis. (b) Composition of the nuclear proteome of HEK-AT_1_R cells 30 min after AngII activation of AT_1_R (list of proteins is shown in Fig. S3). (c) CID spectrum of the Gβ_2_-specific tryptic peptide; peptide coverage is shown in Table S1. (d) CID spectrum of the Gγ_12_-specific tryptic peptide.

To test the hypothesis that agonist activation of GPCRs changes the composition of chromatin-associated proteins, we examined angiotensin II type 1 receptor (AT_1_R), which is a peptide hormone GPCR. Mechanisms of AT_1_R signaling have been extensively studied in the attempt to improve AT_1_R-targeted therapies for hypertension, cardiac hypertrophy and end organ damage. Agonist (e.g., AngII)-mediated activation of AT_1_R has been reported to induce the nuclear mobilization of TFs, including GATA binding protein (GATA4), nuclear factor of activated T cells (NFAT), signal transducer and activator of transcription 3 (STAT3), nuclear factor-kappaB (NF-κB), extracellular signal-regulated kinases (ERK1/2), protein kinase C and HDAC5, during the progression of cardiovascular diseases [22]–[27]. We used a human embryonic kidney (HEK) 293 cell clone, HEK-AT_1_R, as a surrogate model system to identify the proteins that mobilize to the nucleus and associate with chromatin (Fig. 1). In HEK-AT_1_R cells, AngII induced G_q/11_-PLC calcium signaling and pERK1/2 signaling (detailed in Fig. S1). Expression of early growth response genes was subsequently induced a result that was also found in neonatal cardiomyocytes stimulated with AngII [26], [28]. The AngII effects were blocked by treatment with the AT_1_R-selective antagonist, losartan. To prepare the nuclear proteome, AT_1_R was activated for 30 min, which was determined as the time when AngII-induced pERK1/2 association with chromatin was maximal. The nuclear and cytosol subcellular fractions isolated were well separated, as indicated by the absence of Gα subunits in the chromatin preparation and the absence of the histone, H2A, in the cytosolic preparation (Fig. S2). When the nuclear proteome was queried for collision-induced dissociation (CID) spectra of peptides corresponding to abundant plasma membrane and cytosolic proteins, none corresponding to integrins, Gα, GAPDH and cytochrome b5 were detected, which further confirmed the authenticity of our nuclear proteome preparation.

The largest groups of proteins found were RNA-binding proteins, heterogeneous nuclear ribonucleoproteins, splicing factors, nucleolar proteins, ribosomal RNA-binding proteins and the proteins involved either directly in DNA binding or in cell cycle and gene regulation (Fig. 1b, Fig. S3). Most of these molecules are established nuclear proteins and/or shuttling proteins that contain a nuclear localization signal (NLS). Many signaling proteins (13.7%) without an obvious NLS present in nuclear proteome included Gβ_2_, Gγ_12_, and α-actinin-4 (Figs. 1b, S3, S4). The CID spectrum of the signature peptides, LLVSASQDGK for the Gβ_2_ isoform (Fig. 1c) and TASTNNIAQAR for the Gγ_12_ isoform (Fig. 1d; see Table S1 for peptide coverage), were identified by SEQUEST (www.proteomicswiki/index.php/SEQUEST). The Gβ and Gγ subunits form an obligate functional monomer and translocate together. The nuclear partition coefficient estimated by WoLFPSORT (http://wolfpsort.org/) [29] for the Gβ_2_γ_12_ complex was −0.13 (equivalent to HDAC5, which is known to localize in both the nucleus and cytoplasm), indicating the potential for Gβ_2_γ_12_ to enter the nucleus upon GPCR activation. The nuclear partition coefficient of Gγ_12_ alone was −0.13. The nuclear partition coefficient of Gβ_2_ alone was identical to α-actinin-4 (−0.47), which is also known to localize both in the nucleus and cytoplasm [30], suggesting that Gβ_2_ most likely enters the nucleus upon GPCR activation.

### Gβ_2_ content in the nuclear proteome

**Figure 2:**
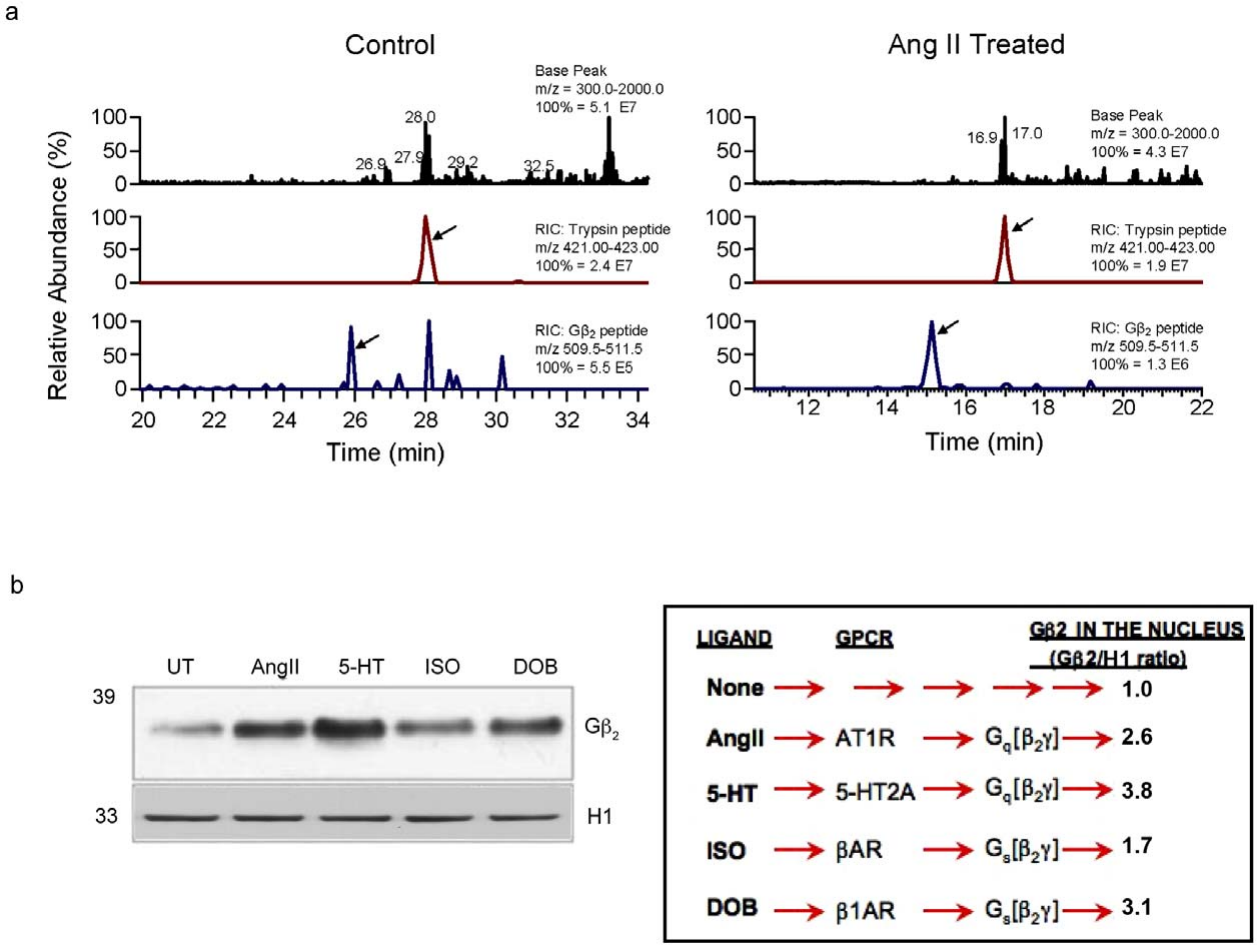
Abundance of the G-protein β_2_ subunit in the nucleus. (a) Mass spectrometry evidence for the differential nuclear translocation of Gβ_2_. The Gβ_2_-specific tryptic peptide (LLVSASQDGK) was monitored in control and AngII treated samples. The right hand corner in each panel gives identity of peptide by m/z ratio and the 100% abundance value of the peptide in that chromatogram. In the chromatogram shown, m/z ratio 509.5–511.5 identified the Gβ_2_ peptide and the 100% abundance value of 1.3E6 after AngII treatment is 2.47-fold higher when compared to the 100% abundance value 5.5E5 of the control. Applying the same calculation, change in abundance of spiked-in control trypsin peptide, m/z 421.0–423 was 0.79. The actual fold change of Gβ_2_ peptide was calculated, 2.47/0.79 = 3.13 in this chromatogram. (b) An increase in Gβ_2_ in the nuclear fraction upon exposure of HASM cells to various prohypertrophic agonists (1 µM AngII for AT_1_R, 1 µM 5-HT for 5-HT2AR; 10 µM isoproterenol for βAR and 1 µM dobutamine for β1AR). Nuclear fractions were immunoblotted for Gβ_2_ and histone H1 as loading controls. See schematic for the relative levels of Gβ_2_ in the nuclei of samples treated with various agonists compared with the untreated (UT) control.

The label-free approach of spectrum counting [31] estimated a significant increase in Gβ_2_ translocation into the nucleus upon AT_1_R activation (Fig. 2a). The abundance of peptides from spiked-in trypsin was comparable in AngII treated HEK-293 and HEK-AT_1_R samples. The relative abundance of the Gβ_2_ peptide, LLVSASQDGK, in the chromatin of AngII activated HEK-AT_1_R sample (NL1.3E6) increased ≈3.1 fold when compared to HEK-293 sample. This fold increase was independently corroborated through additional analysis (Fig. S5).

A variety of prohypertrophic agonists, including AngII, enhanced the nuclear translocation of Gβ_2_ (>1.7 fold) in human aortic smooth muscle (HASM) cells as validated by western blotting (Fig. 2b). The adrenergic receptor agonist (dobutamine) coupled to Gα_s_ was as effective (Fig. 2b, see schematic) in the nuclear mobilization of Gβ_2_ as the Gα_q_-activating agonists (AngII and 5-HT). The activation of different GPCRs may release different Gβγ isoforms, which may participate in chromatin functions with different efficacies. We envision Gβ_2_ as a direct mediator of the nuclear effects of activated GPCRs.

### Agonist-induced nuclear translocation of Gβ_2_

**Figure 3:**
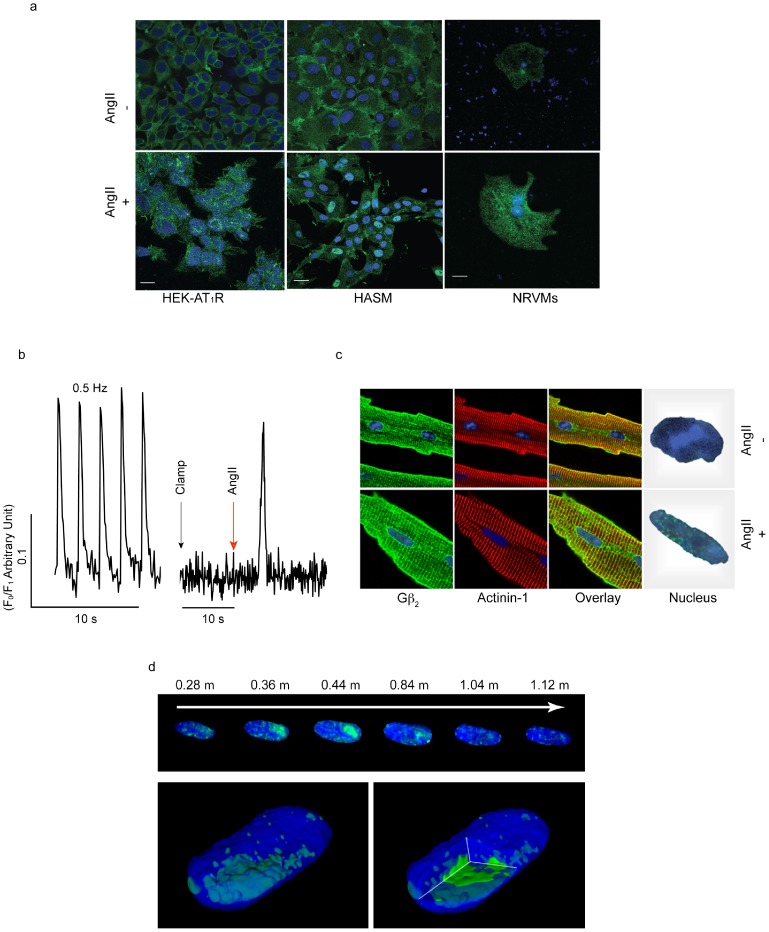
Agonist-activated nuclear translocation of Gβ_2_ in intact cells. (a) The HEK-AT_1_R, HASM and NRVM cells were treated with vehicle or 1 µM AngII for 30 min and fixed. Gβ_2_ is shown in green. The nucleus (blue, stained with DAPI) shows green staining that corresponds to Gβ_2_ in the nuclei. (b) Post-isolation viability and AngII response as assessed by calcium signals in AMVMs. The AMVMs that were paced at a frequency of 0.5 Hz displayed steady-state [Ca^2+^]_i_ transient signals. When AMVM pacing was stopped, the [Ca^2+^]_i_ signals ceased, and upon treatment with 1 µM AngII, the [Ca^2+^]_i_ signal resumed. (c) Beating AMVMs were treated with vehicle or 1 µM AngII for 30 min and fixed. α-Actinin-1 was labeled red and Gβ was labeled green. The far right-hand inset shows a magnified image (1000×) of a single nucleus. The nucleus displays green staining that corresponds to Gβ. Note: α-actinin-1 is a sarcomeric marker and does not translocate to the nucleus. (d) 3-D reconstruction of a mouse cardiac myocyte nucleus (confocal microscopy image). Green fluorescence represents Gβ_2_, and blue represents DAPI staining. The top panel shows the localization of Gβ_2_ (in Z-plane) from the top to the bottom of the myocyte nucleus. The lower panel shows an intact AMVM nucleus and a slice through the nuclear image that depicts a significant accumulation of Gβ_2_ inside the nucleus of the AMVM cell upon AngII/AT_1_R activation. Note: all images were acquired using a 63× objective (1.4 N.A.) at 0.232 µM/pixel in the plane resolution and 0.041 µM/pixel in the Z-axis resolution. The confocal image is a representative image of N = 3, and in each experiment, >50 cells were scored. Scale bars = 50 µm.

Indirect immunofluorescence staining demonstrated an increase in Gβ_2_ in the nucleus of HEK-AT_1_R and HASM cells and neonatal rat ventricular myocytes (NRVMs) upon treatment with AngII (Fig. 3a). Treatment with losartan prevented the increase in Gβ_2_ in the nucleus (Fig. S5a). To confirm that Gβ_2_ translocation was physiologically relevant in cells, we isolated adult mouse ventricular myocytes (AMVMs). Pacing and AngII treatment elicited calcium transients in AMVMs after isolation (Fig. 3b). AngII treatment stimulated the translocation of Gβ_2_ (Fig. 3c) from the cytoplasm into the nucleus of AMVMs (≈4.0-fold). Three-dimensional (3-D) image reconstruction of myocyte nuclei (Fig. 3d) showed the association of Gβ_2_ with chromatin. Thus, using different analytical methods, a 2.5- to 4.5-fold increase in the nuclear translocation of Gβ_2_ was observed in different types of cells upon AngII treatment (Fig. S5b).

### Association of Gβ_2_with components of chromatin

**Figure 4:**
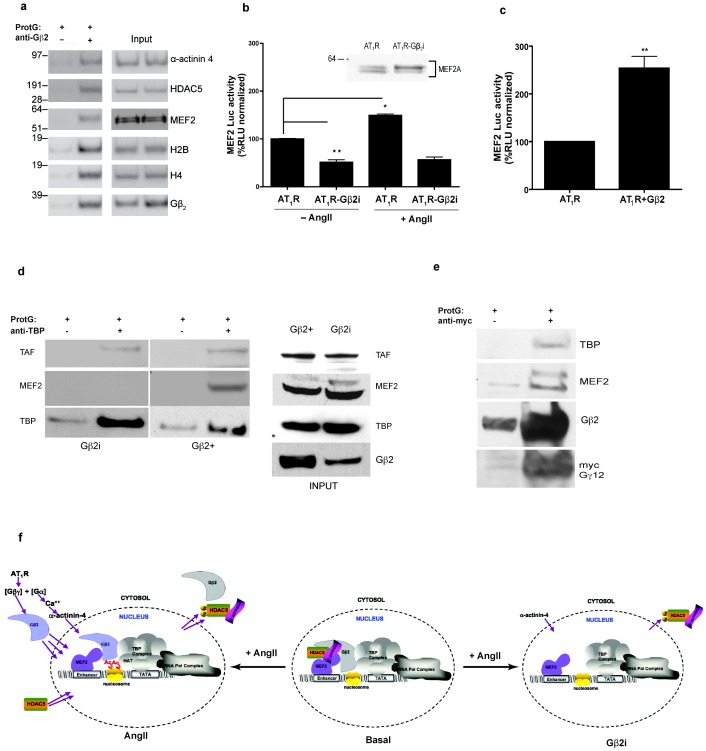
Interaction of Gβ_2_ in the nuclear proteome and mechanism of modulation of MEF2A transcriptional activity. (a) Gβ_2_ coimmunoprecipitates with α-actinin-4, HDAC5, MEF2A, and the histones H2B and H4. The nuclear fractions (100 µg) prepared from HEK-AT_1_R cells treated with AngII (1 µM for 30 min) were subjected to pull-down with only ProtG (−) or with a Gβ_2_ antibody and ProtG (+). The immunoblot on the right shows the abundance of the respective proteins in the immunoprecipitates (− and +) as well as input lysates for the − and + samples. Gel-C peptide index mining provided further supporting evidence for the provisional interactome of nuclear Gβ_2_ (Table S1). (b) A significant increase in MEF2-luciferase activity (*p = 0.039) when AT_1_R was exposed to AngII (bars 1 and 3 from right). The basal MEF2-luciferase activity was significantly (∼50%) attenuated in AT_1_R-Gβ_2_i cells when compared to wild-type AT_1_R (bars 1 and 2; **p = 0.002). The RLU is normalized to co-expressed β-gal activity in each sample. Data were further normalized to basal MEF2-luciferase activity in wild-type AT_1_R cells. Inset: No significant change was detected in MEF2A protein levels in the cell lysate. (c) A significant increase in MEF2-luciferase activity upon FLAG-Gβ_2_ overexpression. (d) In Gβ_2_-positive cells, immunoprecipitation with anti-TBP antibodies revealed the interaction of TBP with MEF2A and TAF. In the absence of Gβ_2_ (Gβ_2_i cells), TBP failed to co-immunoprecipitate MEF2A, but TAF was co-immunoprecipitated. The immunoblot on the right shows the abundance of the TAF, TBP, MEF2A and Gβ_2_ proteins in lysates (INPUT; Gβ_2_ and Gβ_2_i). (e) Upon AT_1_R activation with AngII, the transiently transfected myc-Gγ_12_ translocated to the nucleus with endogenous Gβ_2_ and associated with TBP and MEF2A. (f) Model depicting the modulation of MEF2A-dependent gene transcription by Gβ_2_-associated proteins (MEF2, HDAC5, α-actinin-4, TBP and TAF). **Basal:** In this state, Gβ_2_ forms a complex with MEF2A, HDAC5 and Actinin-4. Histones are deacetylated locally. This yields the basal transcription (for instance, that of MEF2-luciferase). **AngII:** MEF2A forms a complex with Gβ_2_, which also interacts with the TBP-TAF complex. Incoming Gβ_2_ displaces the existing repressor complex (i.e., the α-actinin-4-associated pHDAC is exported into the cytosol). In this state, the recruitment of HATs to the complex results in the acetylation of histones, synergy with the TBP complex, and activation of MEF2-luciferase transcription. **Gβ2i:** In this state, the absence of Gβ_2_ leads to the cytosolic localization of the actinin-HDAC complex, as shown in Figure S8. In addition, MEF2A cannot interact with TBP, which leads to a lack of synergy and the attenuation of basal transcription. Note: the schematic shows no change in the TBP and RNA polymerase complex.

Our nuclear proteome preparation was enriched in protein complexes that were associated with an AngII-activated state of the genome. In this state, Gβ_2_ interacted with the AngII-responsive TF, MEF2A, the core histones, H2B and H4, the histone-modifying enzyme, HDAC5, and the calcium binding scaffold protein, α-actinin-4 (Fig. 4a). Gβ_2_ did not associate with histones H1, H2A and H3 or with pERK1/2.

Gβ_2_ association with MEF2A and histones H2B and H4 suggested that Gβ_2_ interacted with nucleosomes at the promoters of MEF2 regulated genes. The Gβ_2_ interactions with α-actinin-4 and HDAC5 suggested that Gβ_2_ played a role in AngII-mediated remodeling of chromatin by these two proteins. Actinin-1 and -4 are isoforms ubiquitously expressed in non-muscle tissues. They are calcium-sensitive proteins that engage class II HDACs in nucleo-cytoplasmic trafficking [32]. Class II HDACs, including HDAC5, regulate gene expression through association with TFs and alteration of the histone code at gene promoters [5]. We hypothesize that Gβγ is a component of the multiprotein complex at the promoters of MEF2 regulated genes that modulate transcription.

### Essential role of Gβ_2_ in MEF2A-regulated transcription

The mechanism of gene regulation by Gβ_2_ was determined using small interfering RNA (siRNA) based loss-of-function approach that was similar to that used by Krumins and Gilman [33]. The Gβ_2_ mRNA and protein levels were specifically reduced upon stable expression of Gβ_2_-targeted siRNA in cells, hereafter referred to as Gβ_2_i cells, whereas mRNA levels of other Gβ isoforms remained unchanged (Fig. S6, a–b). Normal Gq mediated signals upon activation of AT_1_R by AngII, such as the activation of ERK1/2 in the cytosol (Fig. S6, c–d), the accumulation of pERK1/2 in the nucleus (Fig. S6, e–f) and the mobilization of calcium from intracellular stores (Fig. S6g) remained unaltered in Gβ_2_i cells.

In the Gβ_2_i and control HEK-AT_1_R cell lysates, MEF2A protein levels were similar (Fig. 4b; inset). But the basal MEF2-luciferase reporter gene expression driven by the MEF2A protein was significantly reduced in the Gβ_2_i cells, and AngII treatment did not increase MEF2-luciferase. In the HEK-AT_1_R cells, AngII treatment increased MEF2-luciferase, whereas the AT_1_R blockers, losartan and candesartan, antagonized the AngII-mediated increase in MEF2-luciferase (Fig. S7a and S7b). The overexpression of Gβ_2_ in HEK-AT_1_R cells further increased AngII-mediated MEF2-luciferase (Fig. 4c). In the Gβ_2_i cells, α-actinin-4 and the α-actinin-associated HDACs were sequestered in the cytoplasm (Fig. S8a and S8b), suggesting that these shuttling proteins preferentially remained in the cytosol when Gβ_2_ was knocked down. The cytoplasmic sequestration of α-actinin-4-HDAC has been shown to act as a mechanism to increase MEF2A-dependent transcription [32]. However, the reduction of basal and AngII-induced MEF2-luciferase in Gβ_2_i cells when sufficient MEF2A was present indicates that Gβ_2_ plays a novel role in MEF2-luciferase gene transcription in normal cells.

We propose that the interaction between Gβ_2_ and MEF2A proteins is a GPCR-specific transcriptional cue that facilitates synergy between the MEF2A and TATA-binding protein (TBP) and transcription activating factor (TAF) complex in modulating transcription. As shown in Fig. 4d, in the presence of Gβ_2_, MEF2A interacted with the TBP/TAF complex (reverse co-immunoprecipitations (co-IPs) are shown in Fig. S9a). Knockdown of Gβ_2_ in the Gβ_i_ cells specifically disrupted the interaction of MEF2A with TBP; however, the interaction between TBP and TAF was not affected. These results suggest that the synergy between the MEF2A and TBP/TAF complex requires Gβ_2_. Hence, the knock-down of Gβ_2_ accounts for the decrease in basal as well as AngII-activated expression of MEF2-luciferase. In Fig. 4e, we independently assessed the nuclear localization of myc-tagged Gγ_12_ in HEK-AT_1_R cells upon AngII treatment. The myc-tagged Gγ_12_ associated with endogenous Gβ_2_, MEF2A and TBP. Thus, a novel Gβ_2_γ_12_-dependent multiprotein complex is formed in the nucleus and is essential for the transcriptional activation of the MEF2 promoter.

Previous studies have shown that class II HDACs directly interact with MEF2A and repress transcription through histones deacetylation [5]. The MEF2A-HDAC interaction is dynamically regulated. Our results indicate that weak basal transcription may result from the involvement of Gβ_2_ in a complex with TBP/TAF and MEF2A-HDAC, as the nucleosomes were deacetylated locally in this state (basal in Fig. 4f). An agonist-activated increase in Gβ_2_ in the nucleus is expected to generate a nascent enhancer complex in which Gβ_2_ interacts with TBP/TAF and MEF2A without HDAC5. This complex may facilitate recruitment of HATs, leading to local acetylation of histones and promoting AngII-stimulated transcription. The knockdown of Gβ_2_ weakened the synergy between TAF/TBP and MEF2A, thereby attenuating transcription. The cytoplasmic localization of α-actinin-4 and HDAC in the Gβ_2_i cells suggests that HDAC shuttling regulates Gβ_2_-dependent transcription in the nucleus.

### WD repeat structure in Gβ_2_ is essential for its interaction with MEF2A

To gain insight into the molecular interaction between Gβ_2_ and MEF2A, we used a mutagenesis approach. Each of the 7 WD repeats was sequentially deleted to create seven ΔWD mutants (Fig. S9b). All WD repeat deletion mutants interacted with MEF2A (Fig. S9c), and the ΔWD2, ΔWD3, and ΔWD5–ΔWD7 mutants stimulated MEF2A function, whereas the ΔWD1 mutant did not (Fig. S9d). Therefore, potentiating the MEF2A function appears to require a structure that includes the WD1 repeat of Gβ_2_. Rather surprisingly, the ΔWD4 mutant had a significant stimulatory effect, suggesting that WD4 in Gβ_2_ might be the site of its interaction with HDACs (which are MEF2A co-repressors). Thus, the WD1 repeat of Gβ_2_ is essential for promoting MEF2A function; however, all of the WD repeats contributed to interaction between Gβ_2_ and MEF2A. This led us to hypothesize that MEF2A makes contact with the central canal of the Gβ_2_ toroidal structure.

### Gβ_2_ interacts with TFs that share an amino acid sequence motif

**Figure 5:**
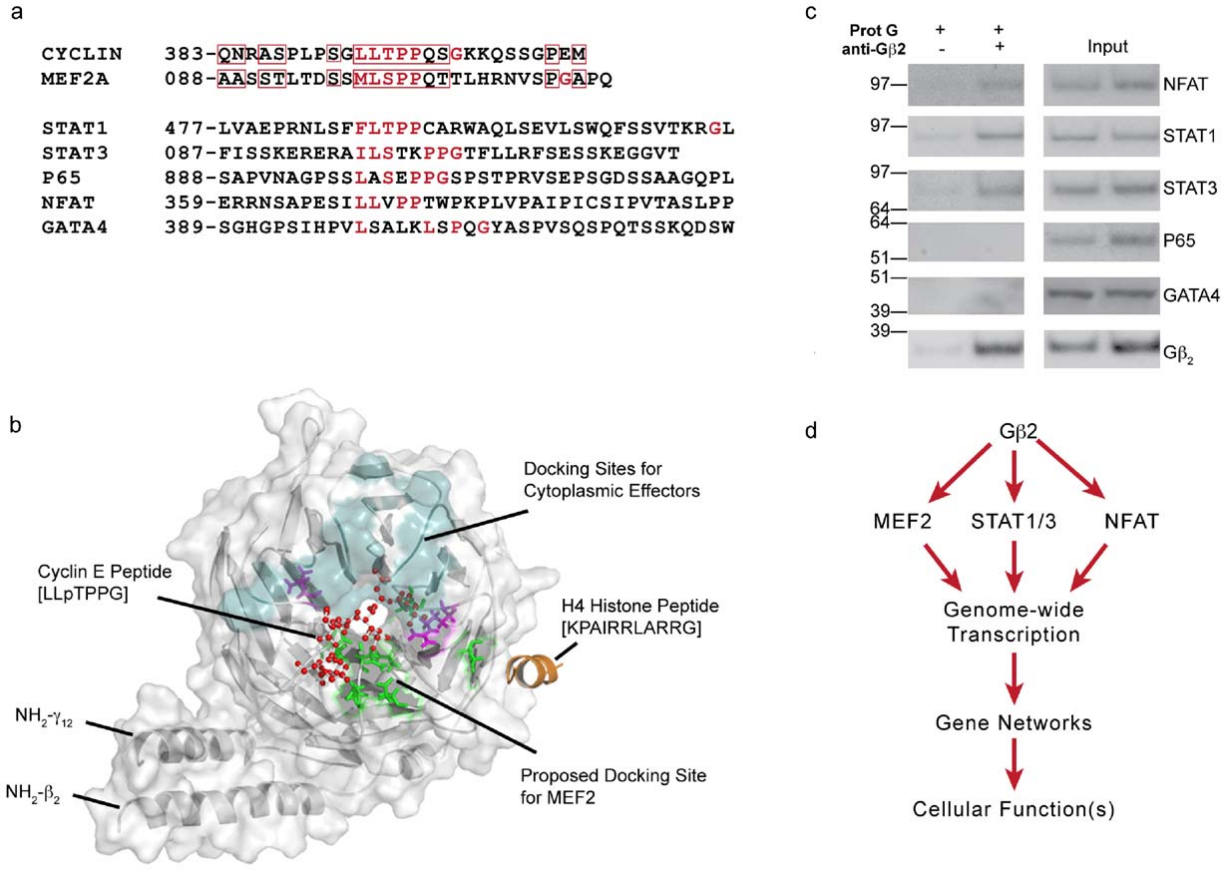
3-D model of the proposed Gβ_2_ interaction with MEF2A and histone H4. (a) Multiple sequence alignment using the CLUSTAL W program revealed that the phosphopeptide motif (–LLpTPPG–) was conserved in the TFs that associated with Gβ_2_ (MEF2A, STAT1, STAT3 and NFAT), but not in NFκB and GATA4. (b) Surface model of Gβ_2_γ_12_ based on Gβ_1_γ_2_ crystal coordinates. Gβ_2_γ_12_ is shown in gray, and the common site of interaction with cytoplasmic effectors (Gα and PLCβ) is shown in teal. The –LLpTPPG– peptide (shown as red ball and stick) anchors to the central core of the β-propeller structure and makes contact with the amino acid residues shown in green and purple. The purple side chains contacting the peptide are conserved charge interactions. The histone H4 tail peptide, shown in brown, may interact on the surface of the WD7 repeat. (c) Co-immunoprecipitation of Gβ_2_ with the AngII-responsive TFs, NFAT, STAT1, and STAT3, but not with GATA4 and p65 NFκB. The nuclear fractions (100 µg) prepared from HEK-AT_1_R cells treated with AngII (1 µM for 30 min) were subjected to pull-down with only ProtG (−) or with a Gβ_2_ antibody and ProtG (+). The immunoblot on the right shows the abundance of the respective proteins in the immunoprecipitates (− and +) and input lysates for the − and + samples. (d) Gβ_2_ interaction with selective AngII-responsive TFs, suggesting a role for Gβ_2_ in genome wide transcription that eventually leads to changes in cellular functions.

To localize a putative MEF2A binding site on Gβ_2_γ_12_, we evaluated chromatin-associated proteins that have been proven to interact with the central canal of the β-propeller proteins (see the methods). Combining molecular modeling, bioinformatics and evolutionary relationship (interactions of vertebrate β-propeller proteins in chromatin are not known) created a model for the interactions of MEF2A and histones with Gβ_2_. Several proteins that bind to the β-propeller central canal, including cyclin E binding to Cdc4, use a phosphopeptide (–LLTPPG–) docking site [34]–[36]. A similar sequence motif was conserved in MEF2A, and when aligned, an extended homology with the cyclin E region was found (Fig. 5a). Molecular modeling and docking experiments (detailed in the methods) indicated that MEF2A could dock at the central canal of Gβ_2_ (Fig. 5b) and that the proposed MEF2A binding site should not overlap with the interaction sites for cytoplasmic effectors of Gβ_2_ [37]. The histone H4 peptide binds WD7 in *Drosophila* β-propeller protein p55 [38]. In the Gβ_2_γ_12_ model (Fig. 5b), the binding site for histone H4 was conserved; thus, WD7 in Gβ_2_ may interact with the nucleosome.

Bioinformatic analysis revealed that one copy of the –LLTPPG– motif was conserved in several AngII-responsive TFs, including STAT1/3 and NFAT, but not in NFκB p65 or GATA4 (Fig. 5a). We tested this prediction by protein interaction analysis, and the data revealed that Gβ_2_ indeed associated specifically with NFAT and STAT1/3, but not with NFκB p65 or GATA4 (Fig. 5c). By interacting with multiple TFs via the conserved –LLTPPG– motif, Gβ_2_ can coordinate the expression of multiple target genes. The human genome harbors >10^5^ sites for each of the Gβ_2_-interacting TFs, and transcription at some of these sites must be Gβ_2_-dependent in response to GPCR agonists. Gβ_2_ and other Gβγ isoforms may also coordinate GPCR-dependent gene transcription (Fig. 5d). The clinical success of GPCR-targeted drugs indicates that the therapeutic benefits of these drugs potentially include modulation of Gβγ-dependent chromatin remodeling. These insights led us to investigate the genome-wide transcription profile upon knockdown of Gβ_2_.

### Gβ_2_-dependent global gene expression pattern

**Figure 6:**
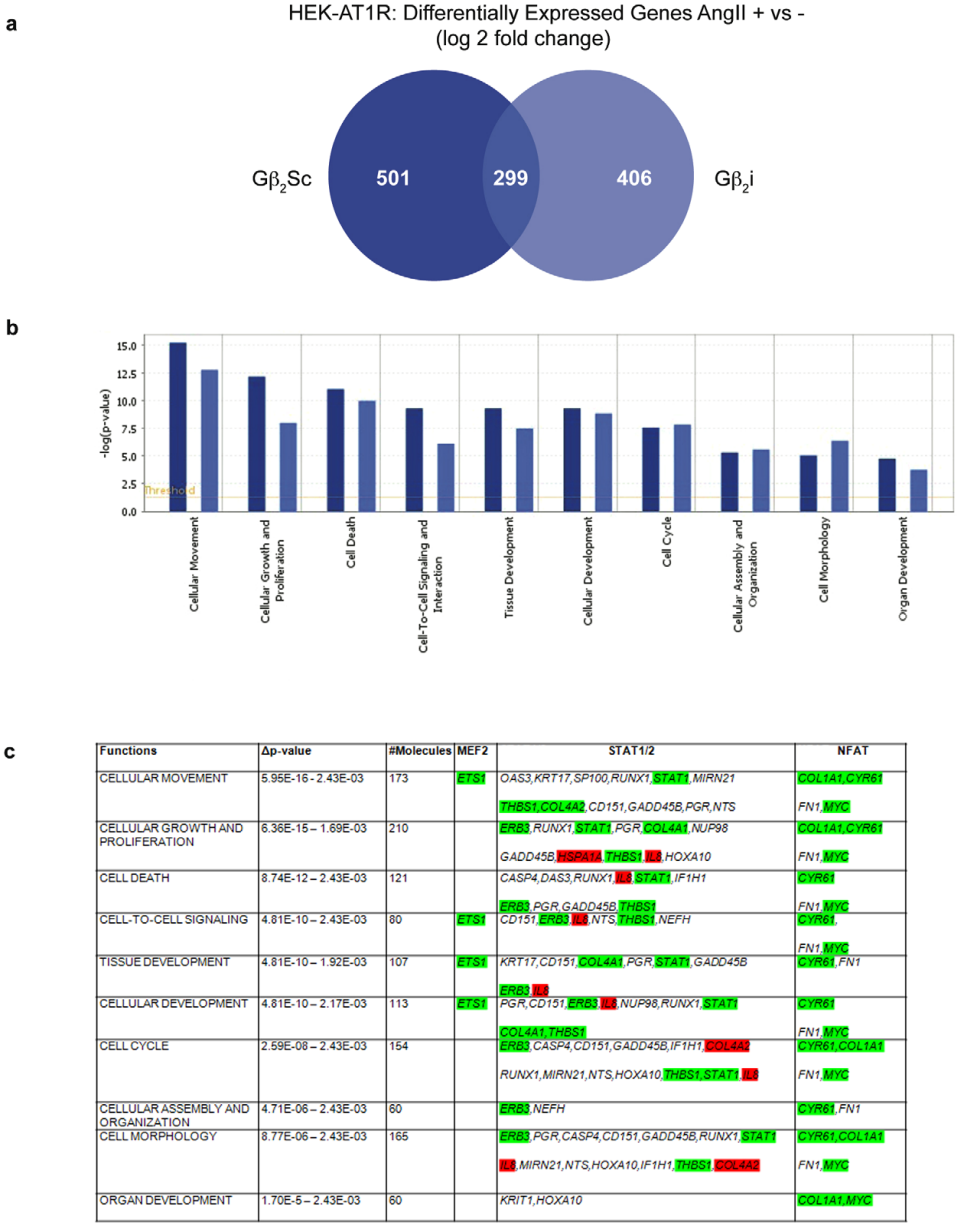
Gβ_2_-dependent global gene expression patterns. Altered gene expression patterns and gene networks that engage common biological processes are shown. Of the >47,000 transcripts monitored, 705 unique and annotated transcripts (2% of the transcriptome) were differentially affected by AngII stimulation in the Gβ_2_i cells (see Table S5). Out of these, 299 transcripts were identical to the transcripts in the Gβ_2_Sc control, indicating that these transcripts were regulated by Gβ_2_-independent signals from AT_1_R, and the remaining ≈400 transcripts were specifically regulated by Gβ_2_. The false discovery rate was <3%. (a) Venn diagram: a total of 800 genes were modulated in Gβ_2_Sc cells, and 705 genes were modulated upon Gβ_2_ knockdown (Gβ_2_i) in AT_1_R-expressing cells treated with AngII (1 µM for 30 min). (b) The altered cell functions upon Gβ_2_ knockdown. (c) The hierarchy of gene functions, Δ*p*-value, number of molecules involved and genes regulated by the Gβ_2_-interacting TFs, MEF2A, NFAT, and STAT1/STAT3 (derived from the ‘Build Networks – Expand by one group interaction’ algorithm in MetaCore™). Shown in green are down regulated genes, in red are up regulated genes in an independent experiment. Additional promoter information is shown in Table S2.

Expression profiling indicated that ≈400 transcripts were differentially regulated by Gβ_2_-dependent signals (Fig. 6a, Table S5). The Ingenuity Pathway analysis sorted the expression data to gene networks that reflected the capacity of the gene products (i.e., receptors, enzymes, scaffold proteins, and extracellular matrix components) to influence specific cellular functions. The most prominent cellular functions that were altered are shown in Figure 6b. Each of the significantly altered cellular functions consisted of a network of >60 molecules (Fig. 6c), indicating that Gβ_2_ knockdown substantially altered gene regulation (see Δp-value). Gβ_2_ knockdown transformed the “cellular growth and function” network to the “cellular growth and function in disease” network (Fig. S10). The network score of 41 before Gβ_2_ knockdown indicated that there was a 10^−41^ chance that these genes were randomly present in the network. The network score after the knockdown was 40. Sixteen core molecules were unaffected by Gβ_2_ knockdown. The functional change in the Gβ_2_i cells appeared to be due to co-opted PM-resident transmembrane proteins (e.g., platelet-derived growth factor receptors and integrins) and secreted proteins (e.g., interleukin-1, serine protease inhibitors of the SERPIN gene family, insulin-like growth factor-1, and platelet-derived growth factor). Interestingly, the promoters of differentially expressed genes contained the binding sites for one or more of the TFs that associated with Gβ_2_ (Table S2). Analysis using Metacore™ revealed a set of genes (Fig. 6c) that were transcriptionally regulated by MEF2A, NFAT, STAT1, and STAT3. Gβ_2_ knockdown affected AngII-dependent transcription of these genes as confirmed by real time RTPCR, which may be the direct cause for the transformation of network function. *In vivo*, when the AT_1_R stimulus becomes chronic or when Gβ_2_ is not regulated properly, the dynamics of the signaling networks might tilt towards a disease state that can promote damage to the tissue as well as contribute to chronic disorders. We conclude that Gβ_2_ is a master regulator of gene expression programs in response to agonist activation of AT_1_R and likewise other the GPCRs.

## Discussion

PM-to-nuclear translocation of Gβγ and its regulation of nuclear effectors is a novel paradigm in GPCR signaling. The most common role for Gβγ may be in mediating synergy between different transcriptional regulatory complexes at gene promoters. The specific and dynamic changes that are orchestrated by Gβγ could involve facilitating the interaction of the enhancer complex (MEF2A) with the TFIID complex (TBP/TAF), the stepwise dissociation of negative regulators (HDACs) from transcriptional regulatory complexes and the association of positive regulators (HATs, co-activators). Novel nuclear targets of Gβγ were identified in the present work, and the ability of the Gβγ complex to regulate nuclear translocation of glucocorticoid receptor [9] and HDAC5 [10], [11] has been previously reported. The ability of Gβγ to facilitate interactions between multiple proteins that are involved in gene regulatory complexes can explain the signaling specificity and the high-level transcriptional output by G-proteins. Many proteins in the multiprotein complex can promote gene expression individually; however, none of these components, with the exception of Gβγ can function unequivocally as a GPCR-specific enhancer of gene transcription.

In specialized cells, such as cardiac and smooth muscle cells, the intracellular distribution of some GPCRs, including the AT_1_R, and G-proteins has been reported [39]. A consensus regarding how GPCRs signal in the subcellular compartments apart from PM resident GPCRs or how intracellular and cell surface GPCR signaling coordinated is still evolving. It is possible that extracellular agonists reach the intracellular compartments, such as the nucleus and promote local G-protein signaling in specialized cells. Local G-protein activation may also regulate the nuclear targets of Gβγ as well as the generalized retrograde translocation of Gβγ from the PM to the nucleus.

A direct role for G-protein subunits in orchestrating gene responses to GPCRs is thought to be limited because the repertoire of conventional signaling targets of heterotrimeric G-proteins are localized in the PM and cytosol. The discovery of Gβ_2_ translocation to the nucleus and its role in the regulation of gene networks define Gβγ as a key missing link through which GPCRs modulate gene expression. A variety of GPCR agonists promote the nuclear translocation of Gβγ in physiologically relevant cells, indicating its universal significance.

WD repeat β-propeller proteins are integral components of chromatin-modifying complexes in lower eukaryotes [40]. Members of this family (>165 proteins) exhibit similar structures and remarkably, perform similar types of nuclear functions [40]–[45]. However, the nuclear functions of heterotrimeric Gβγ proteins, which are the founding members of the β-propeller protein family, have remained elusive. Taken together with previous data [8]–[12], our findings suggest that Gβγ proteins mediate chromatin remodeling, which may be an evolutionarily ancient and essential function in vertebrates. Histone gene clusters and histone-modifying enzymes were indeed modulated in Gβ_2_i cells (Table S3), similar to the regulation of histone genes by β-propeller proteins in drosophila [42] and histone deacetylases in chicken [45]. Thus, controlling the expression of chromatin-regulating complexes may be a critical function of vertebrate Gβγ.

Global gene regulation dependent on Gβγ provides a mechanism for direct gene regulatory function of an activated GPCR in a variety of biological contexts. Nearly 2% of the modulated genes in Gβ_2_i cells are members of the GPCR superfamily or are involved in signal transduction activated by GPCRs (Table S4), and they also include ≈30% of cardiac hypertrophic marker genes (Table S4) [24]. Increased G-protein signaling is a trigger for the reactivation of the fetal gene program, which is a hallmark feature of cardiac hypertrophy and heart failure [24]–[25]. The extent to which deregulation of gene expression *in vivo* is due to extensive reconfiguration of the epigenome and/or involves Gβ_2_ is a critical question that remains to be elucidated. Conceivably, the Gβγ pathway could be targeted pharmacologically to control physiological and pathological chromatin responses and may be particularly useful in the setting of chronic disorders [46], in which dysregulated GPCR signaling is known to play an important role. Therefore, it is essential to gain a better understanding of the role of different Gβγ isoforms in epigenetic regulation.

## Supporting Information

Methods S1 **Details of experimental protocol for some methods described.**
(DOC)

Figure S1 **Pharmacological and biochemical analysis of HEK-293 cells stably expressing HA-tagged AT_1_R.** (a) Scatchard analysis; the kinetics of binding ^126^I-[Sar^1^,Ile^8^] AngII (measured K_d_ (812 pM) and B_max_ (5.3 pmol/mg) to AT_1_R. (b) AngII ligation with AT_1_R mobilizes calcium from intracellular stores. (c) Immunocytochemical analysis of HEK-293 cells stably expressing HA-tagged AT_1_R (labeled green with FITC) and visualized by confocal microscopy. Under quiescent conditions, the receptors are localized at the plasma membrane. Receptor activation with 1 µM AngII caused PM ruffles (white arrows) followed by a significant increase in the immunoreactivity of pERK1/2 (labeled red) in the nucleus (blue) for up to 60 min. Note that the confocal image shown here is after 10 min of AngII stimulation. In all subsequent experiments, 30 min of stimulation was used.
(TIF)

Figure S2 **Preparation and validation of the chromatin proteome.** Nuclear fraction extraction for mass spectrometry analysis. Cytoplasmic and nuclear fractions were prepared from untransfected (UT) and AT_1_R-expressing HEK-293 cells treated with different ligands (AngII, losartan and candesartan). Fifty micrograms of protein was loaded onto 10% Nu-PAGE gels and subjected to western blot analysis. The G-protein α-subunit, Gαq was only found in the cytoplasmic fraction, whereas histone H2A was found in the nucleus, and T-ERK1/2 was present in both fractions. Note: the chromatin proteome was queried for CID spectra of peptides corresponding to plasma membrane and cytosolic marker proteins (e.g., integrins, Gα, GAPDH, βactin, and cytochrome b5). None of the peptides corresponding to the above abundant proteins were detected in the nucleus, which confirms the fractionation procedure.
(TIF)

Figure S3 **Classification of the chromatin proteome of AT_1_R-activated cells.** All peaks with at least 15 product ions in the MS/MS spectra were extracted. The peak lists from three replicate experiments were searched against mouse and rat reference sequences using search parameters for human protein tryptic fragments and allowing for standard modifications and cleavage variation (1 missed cleavage/peptide). Quantitative analysis was performed by label-free spectrum counting after applying a threshold peptide ion score of 30 for MS/MS interpretation. All peptides were manually validated. The minimum criterion for positive identification of any protein was the presence of one signature peptide with a manually validated CID spectra. A total of 173 proteins were present on the peak list, of which 137 proteins met the selection criteria applied.
(TIF)

Figure S4 **The CID spectra of α-actinin-4 peptides.** The chromatin proteome of AT_1_R-activated cells consisted of peptides (CISQEQMOXQEFR, TINEVENQILTR, FAIQDISVEETSAK) assigned (MASCOT/NCBI non-redundant database) to α-actinin-4.
(TIF)

Figure S5 **Gβ2 accumulates in the nucleus upon AT_1_R activation by AngII and is blocked by treatment with the AT_1_R antagonist, losartan.** (a) The HEK-AT_1_R cells untreated or treated with 1 µM losartan and HA-AT_1_R were labeled red, and Gβ2 was labeled green. The inset in the right top corner of the middle Gβ2 panel shows a magnified image (1000×) of a single cell (arrow in overlay). The nucleus of the cell shows green staining that corresponds to Gβ2 in the nuclei. (b) Different analytical methods, including mass spectrometry (MS), immunoblot (IB) analysis and immunocytochemistry (ICC), showed equivalent fold changes in Gβ2 accumulation in the nucleus upon AT_1_R activation. A pixel counting approach estimated (50 cells, n = 3) that ∼30% of the Gβ2 pool was localized in the nucleus when AT_1_R was activated with 1 µM AngII for 30 min in HASM and HEK-AT_1_R cells. This distribution accounts for ∼2.5–4.5-fold increases in Gβ2 levels in the nucleus which is similar to that estimated by other methods.
(TIF)

Figure S6 **AT_1_R-mediated cytoplasmic signaling events are unaffected upon RNAi-mediated silencing of Gβ2.** (a) Total lysates were prepared from untransfected HEK293 cells, dual plasmid-transfected clones expressing AT_1_R with scrambled Gβ2-scrambled (Gβ2Sc) and AT_1_R with a Gβ2RNAi plasmid. Lysates were subjected to immunoblot analysis to detect AT_1_R expression (anti-HA), Gβ (pan antibody) and β actin (loading control). Both of the cell lines exhibited equivalent levels of AT_1_R. The B_max_ (maximal specific binding) obtained for AT_1_R-Gβ2Sc was 8.7+/−0.9 pmol/mg and 9.7+/−0.9 pmol.mg for AT_1_R-Gβ2i with a K_d_ value of 1732.5+/−170 pM. Taken together, both cell lines expressed comparable levels of AT_1_R. (b) Table showing the Affymetrix array gene expression data from Gβ2i stable cell lines compared to Gβ2+ cells revealed a knockdown specifically for GNB2. (c–d) Both cell types were serum starved for a minimum of 18 hr and then exposed to vehicle (−) or 1 µM AngII (+) for 5, 10, 15, 20, 30 and 60 min. Lysates were immunoblotted for pERK1/2 and total ERK1/2 in Gβ2Sc and Gβ2i cells. The phosphorylation of ERK1/2 upon AngII activation of AT_1_R was preserved in the absence of Gβ2. (e–f) Immunocytochemical analysis followed by confocal imaging of pERK1/2 (labeled green) localized in the nuclei (labeled blue with DAPl) in Gβ2Sc and Gβ2i cells upon AT_1_R activation with AngII. (g) Calcium mobilization upon AngII activation of AT_1_R was preserved in Gβ2Sc and Gβ2i cells (fluorescence-based assay using FLEX Station 3).
(TIF)

Figure S7 **The AT_1_R blockers, losartan and candesartan, prevented the increase in AngII-mediated MEF2 reporter activity.** (a) AngII treatment increased MEF2-luciferase expression, and this increase was blocked by treatment with the AT_1_R antagonist, losartan (∼54%), and (b) candesartan (∼96%). Note: losartan is a less potent AT_1_R antagonist compared to candesartan. Data are expressed as % RLU normalized to the AngII response (100%) with losartan/candesartan alone as 0%. Error bars indicate standard error of the mean (n = 3) of experiments performed in duplicate. *P* values were * = 0.03 and ** = 0.02 using an unpaired t-test (two-tailed with Welch's correction in GraphPad Prism software).
(TIF)

Figure S8 **Gβ2 modulates the export of the α-actinin-4-HDAC complex from the nucleus to the cytosol.** (a) Immunocytochemical analysis of AT_1_R and AT_1_R-Gβ2RNAi cells revealed increased cytoplasmic localization of α-actinin-4 (green) compared with control. (b) An actinin-associated HDAC activity assay on the cytosolic fraction of AT_1_R in AT_1_R-Gβ2 RNAi cells (no agonist and AngII 1 µM for 30 min). There was a significant increase in actinin-associated HDAC activity upon AngII treatment of AT_1_R cells. There was a significant increase under quiescent conditions in Gβ2i cells (no agonist). *P* value: * = 0.049 and † = 0.034. No significant change was observed upon agonist exposure under conditions of Gβ2 knockdown.
(TIF)

Figure S9 **The WD repeats in Gβ2 form a platform to allow for the formation of a multimeric protein complex.** Reverse Co-IPs were performed with Prot G (−) and Prot G (+) antibodies. (a) Gβ2, MEF2, TBP and TAF antibodies (+) were used for immunoprecipitation (IP), and the samples were immunoblotted (IB) for interacting proteins as shown here. (b) Schematic representation of sequential WD repeat deletions in Gβ2. (c) Co-immunoprecipitation with anti-M2 FLAG beads in FLAG-Gβ2/mutants and MEF2-expressing cells showed no significant change in MEF2 association with Gβ2 (n = 3). (d) MEF2 functional activity stimulated by Gβ2/WD repeat deletion mutants. Error bars indicate the standard error of the mean (n = 3), and *P* values were †, ‡ <0.003 and * = 0.011 as calculated using an unpaired t-test (two-tailed) with Welch's correction in GraphPad Prism software.
(TIF)

Figure S10 **Influence of Gβ2 knockdown on the cellular growth and proliferation network.** **The knockdown of Gβ2 affected function of this network.** (a) The *Cellular Growth and Proliferation Network* was derived from the IPA analysis of differentially regulated genes in the wild-type cells (in AngII vs. untreated cells). (b) The *Cellular Growth and Proliferation Network* derived from differentially regulated genes in the Gβ2i cells (in AngII vs. untreated cells). The assigned function for the *Cellular Growth and Proliferation Network* in Gβ2i cells (i.e., cellular growth and proliferation in connective tissue disorders and in nervous system development and function). (c) The *Cellular Growth and Proliferation Network* derived from Gβ2i cells is superimposed onto the wild-type network and shows the presence of molecules that now participate in the network and thus assigns it new specialized function.
(TIF)

Table S1 **The Peptide Index.**
(DOC)

Table S2 **Location of Binding Sites for Gβ_2_ Interacting Transcription Factors within Promoters of Genes Associated with Cellular Growth and Proliferation Network.**
(DOC)

Table S3 **Gβ_2_ Modulates the Expression of Histone and Histone Modifier Genes.**
(DOC)

Table S4 **Gβ_2_ Modulates the Expression of Genes Involved in G-Protein Coupled Receptor Signaling.**
(DOC)

Table S5 **Differentially Expressed Genes (p<0.01) Upon Gβ_2_ Knockdown.**
(DOC)
