# Correction: Interaction of G-Protein βγ Complex with Chromatin Modulates GPCR-Dependent Gene Regulation

**DOI:** 10.1371/journal.pone.0155198

**Published:** 2016-05-04

**Authors:** Anushree Bhatnagar, Hamiyet Unal, Rajaganapathi Jagannathan, Suma Kaveti, Zhong-Hui Duan, Sandro Yong, Amit Vasanji, Michael Kinter, Russell Desnoyer, Sadashiva S. Karnik

The authors would like to correct an error in Fig 4 that was introduced during the preparation of this figure for publication. In Fig 4A the two bottom left panels of H4 duplicate the Gβ_2_ panels. The H4 panels in Fig 4A have been corrected. The HDAC5 panel in Fig 4A duplicates the STAT1 panel shown in Fig 5C. The HDAC5 panel in Fig 4A has been corrected.

The authors have stated that cropped blots were provided to a colleague to create the finalized image for publication. The error was introduced to Fig 4 at that point in time, and the original blots were not retained because of a change of institution. The authors confirm that these changes do not alter their findings.

The revised Fig 4A can be downloaded from this correction as S1 Fig.

## Supporting Information

S1 Fig Fig 4A showing incorrect and revised blots. (TIF)
