# Supplementary material for: Interaction of G-Protein βγ Complex with Chromatin Modulates GPCR-Dependent Gene Regulation
Source: PLoS One. 2013 Jan 9;8(1):e52689. doi: 10.1371/journal.pone.0052689 (PMC3541368; doi:10.1371/journal.pone.0052689)
Supplement: Methods S1 — Details of experimental protocol for some methods described. (DOC) [file pone.0052689.s001.doc]

**METHODS S1**

nuclear and cytosolic fractions: The nuclei isolation kit (NUC101) was used as detailed by the manufacturer (Sigma-Aldrich). We used the appropriate markers for the cytosol and nuclei to systematically assess the purity of the fractions (Fig. S2). Briefly, 50–60% confluent cells in 10-cm plates from control or AT_1_R-activated groups were used for mass spectrometry analysis. Cells were subjected to fractionation per the manufacturer's guidelines. The supernatants from the first centrifugation at 1500 x *g* constituted the cytosolic fraction (S1). The resuspended pellet (nuclei) was washed twice with the same buffer. Staining with DAPI and subsequent visualization was performed using confocal microscopy to check for the integrity of the nuclei. Nuclear proteins were extracted using benzonase (10 units/ml at 37ºC for 60 min), which essentially digests the nucleic acids without altering the protein (chromatin proteins). The pellet was further extracted to isolate the tightly bound proteins (acid fractions). The purity of the fractions was ascertained by immunoblotting for specific cellular compartment markers, histone H2A (nuclear) and G-protein α-subunit (cytosolic).

mef2 reporter assay: The MEF2 reporter assay (Promega) was performed per the manufacturer’s guidelines. Briefly, 1 μg of the MEF2-luciferase reporter plasmid was transfected into AT_1_R-expressing cells in the presence or absence of Gβ2 to evaluate the role of Gβ2 in modulating MEF2 activity. Further, the N-terminal FLAG-tagged Gβ2 and sequentially deleted N-terminal FLAG-tagged Gβ2 construct to generate ΔWD1 through ΔWD7 deletion mutants. HEK-AT_1_R cells were then transfected with 1 μg of the MEF2 reporter plasmids and 150 ng of the βGal plasmid (transfection control).

hdac deacetylase assay: The HDAC activity (UPSTATE) was performed per the manufacturer’s guidelines. For deacetylase assays, the nuclear and cytosolic fractions were obtained from an AT_1_R cell line and AT_1_R-Gβ2i cells in the presence or absence of agonist (30 min of 1 μM AngII). To measure the deacetylase activity in the presence or absence of Gβ2 on AT_1_R activation, protein G Sepharose beads were mixed with purified rabbit polyclonal antibodies against actinin (1 μg) and incubated with 100 μg each of either the cytosolic or nuclear fractions. The samples were incubated with gentle mixing at 4ºC overnight. The immunoprecipitates were collected via centrifugation and washed twice with 1 ml of ice-cold PBS. The resin was assayed for deacetylase activity using the commercial HDAC assay kit.

calcium mobilization: Calcium mobilization in response to AngII (1 μM) in HEK-AT_1_R Gβ2sc and HEK-AT_1_R Gβ2i cells was measured in 96-well microplates using Molecular Devices Flexstation3 and the

FLIPR Calcium 4 Assay Kit, which contains the calcium sensitive dye indicators. Upon AngII binding to the receptor, calcium is released into the cytoplasm of the cell.  The dye binds to the intracellular calcium and becomes fluorescent, thus capturing the rapid kinetics of calcium signaling

Detailed in Ref:

Davies MA et al. Pharmacologic analysis of non-synonymous coding h5-HT2A SNPs reveals alterations in atypical antipsychotic and agonist efficacies. *Pharmacogenomics J.* 6(1), 42-51 (2006).
