# Supplementary figures and images for: Interaction of G-Protein βγ Complex with Chromatin Modulates GPCR-Dependent Gene Regulation
Source: PLoS One. 2013 Jan 9;8(1):e52689. doi: 10.1371/journal.pone.0052689 (PMC3541368; doi:10.1371/journal.pone.0052689)

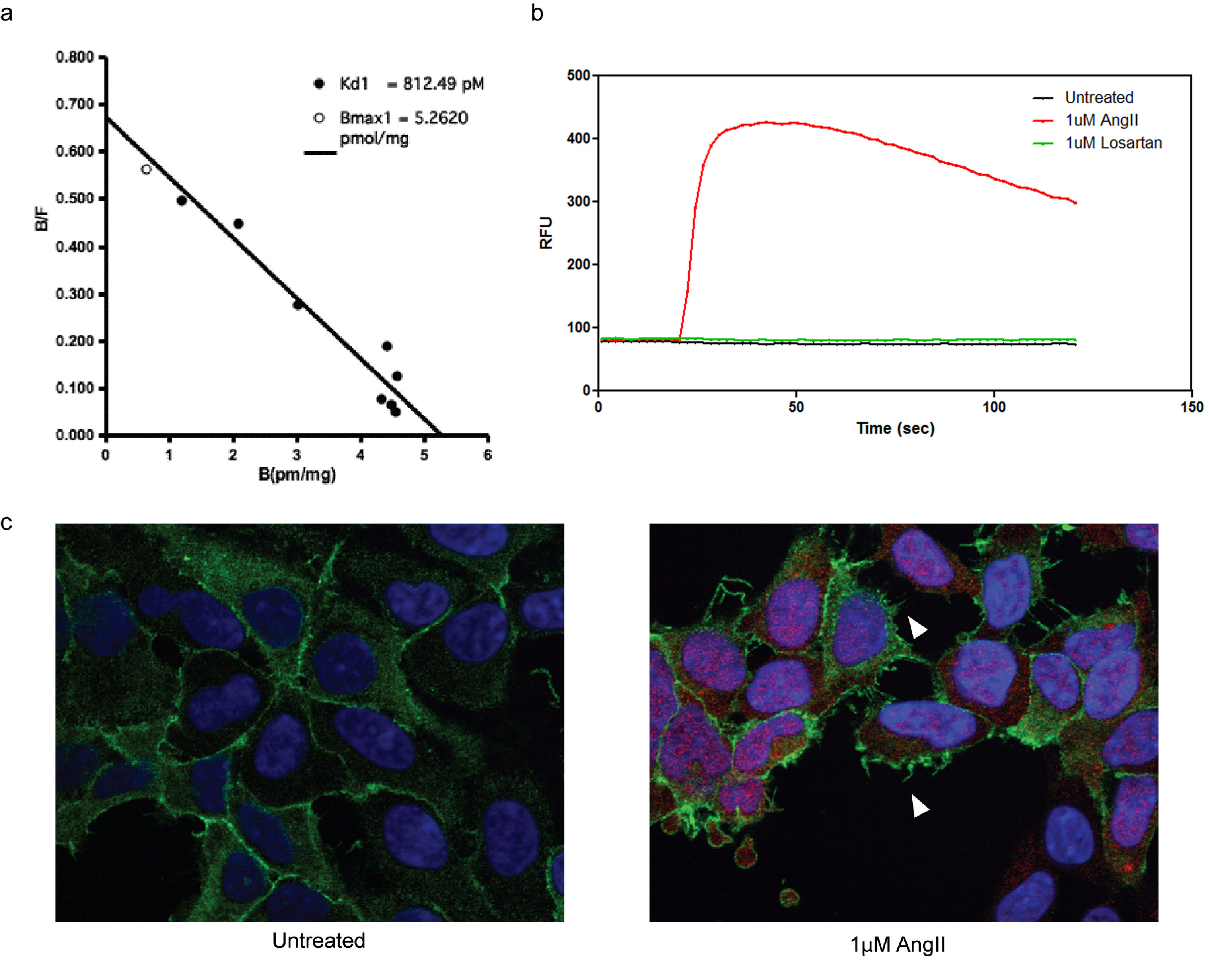

Supplement: Figure S1 — Pharmacological and biochemical analysis of HEK-293 cells stably expressing HA-tagged AT1R. (a) Scatchard analysis; the kinetics of binding 126I-[Sar1,Ile8] AngII (measured Kd (812 pM) and Bmax (5.3 pmol/mg) to AT1R. (b) AngII ligation with AT1R mobilizes calcium from intracellular stores. (c) Immunocytochemical analysis of HEK-293 cells stably expressing HA-tagged AT1R (labeled green with FITC) and visualized by confocal microscopy. Under quiescent conditions, the receptors are localized at the plasma membrane. Receptor activation with 1 µM AngII caused PM ruffles (white arrows) followed by a significant increase in the immunoreactivity of pERK1/2 (labeled red) in the nucleus (blue) for up to 60 min. Note that the confocal image shown here is after 10 min of AngII stimulation. In all subsequent experiments, 30 min of stimulation was used. (TIF) [file pone.0052689.s002.tif]

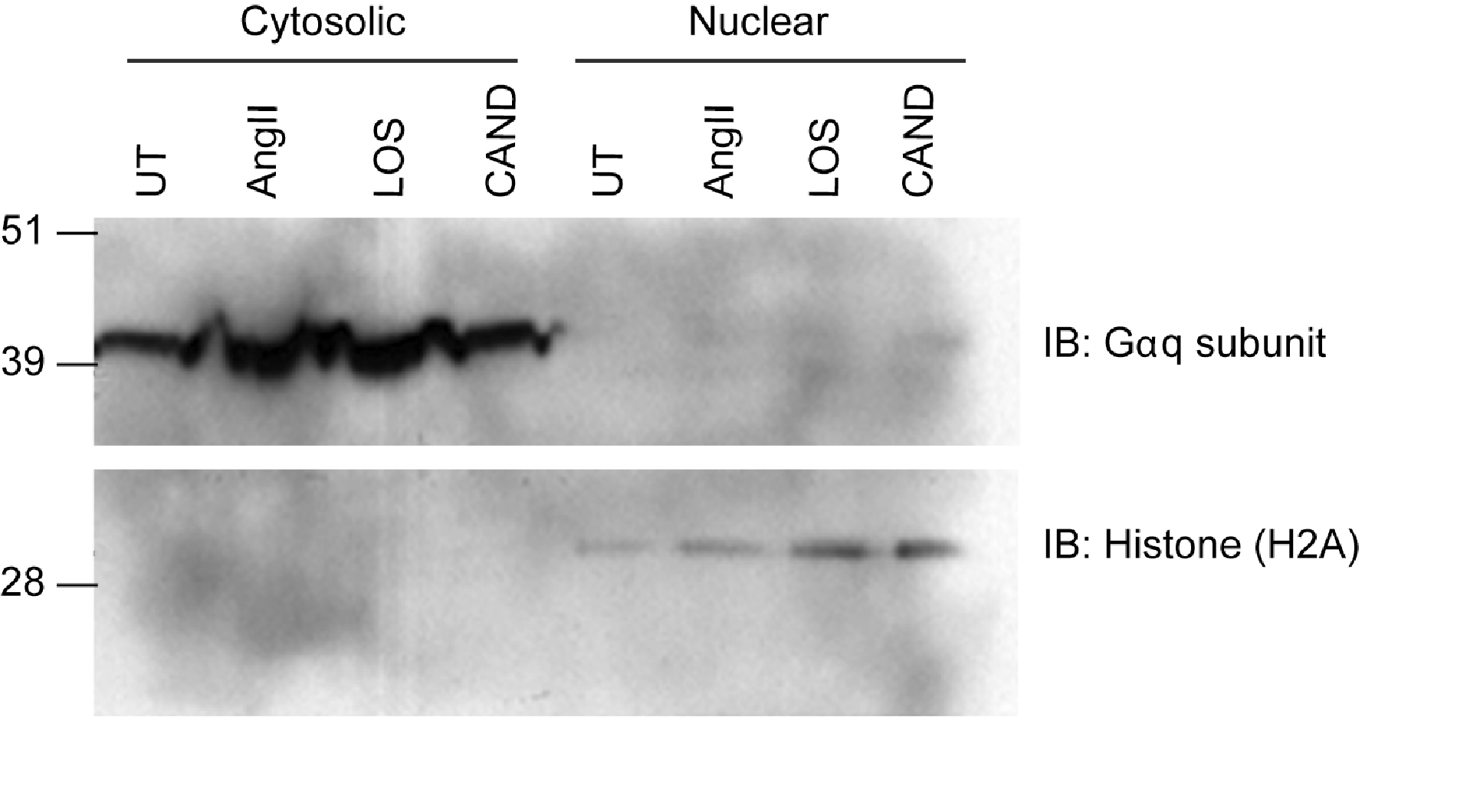

Supplement: Figure S2 — Preparation and validation of the chromatin proteome. Nuclear fraction extraction for mass spectrometry analysis. Cytoplasmic and nuclear fractions were prepared from untransfected (UT) and AT1R-expressing HEK-293 cells treated with different ligands (AngII, losartan and candesartan). Fifty micrograms of protein was loaded onto 10% Nu-PAGE gels and subjected to western blot analysis. The G-protein α-subunit, Gαq was only found in the cytoplasmic fraction, whereas histone H2A was found in the nucleus, and T-ERK1/2 was present in both fractions. Note: the chromatin proteome was queried for CID spectra of peptides corresponding to plasma membrane and cytosolic marker proteins (e.g., integrins, Gα, GAPDH, βactin, and cytochrome b5). None of the peptides corresponding to the above abundant proteins were detected in the nucleus, which confirms the fractionation procedure. (TIF) [file pone.0052689.s003.tif]

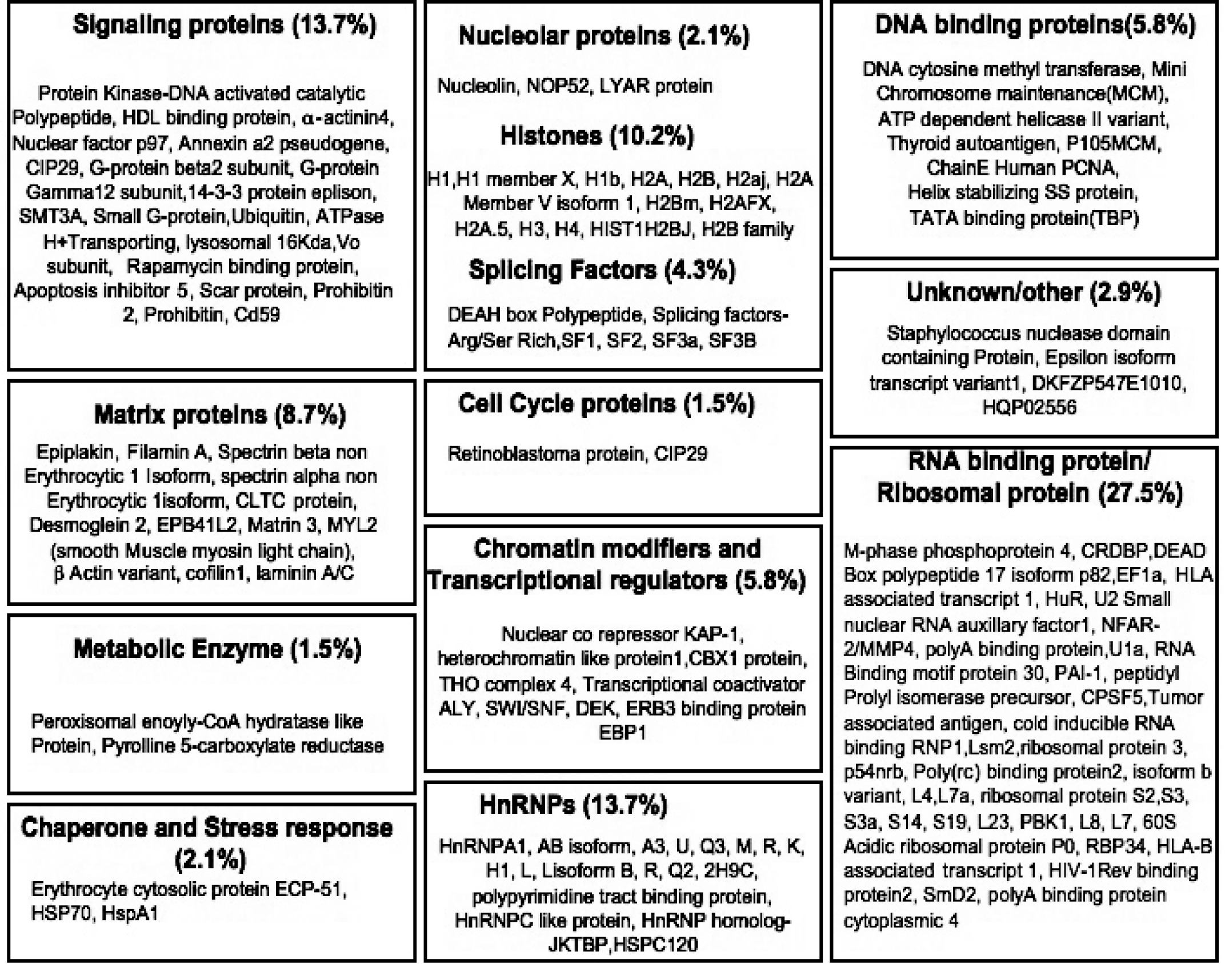

Supplement: Figure S3 — Classification of the chromatin proteome of AT1R-activated cells. All peaks with at least 15 product ions in the MS/MS spectra were extracted. The peak lists from three replicate experiments were searched against mouse and rat reference sequences using search parameters for human protein tryptic fragments and allowing for standard modifications and cleavage variation (1 missed cleavage/peptide). Quantitative analysis was performed by label-free spectrum counting after applying a threshold peptide ion score of 30 for MS/MS interpretation. All peptides were manually validated. The minimum criterion for positive identification of any protein was the presence of one signature peptide with a manually validated CID spectra. A total of 173 proteins were present on the peak list, of which 137 proteins met the selection criteria applied. (TIF) [file pone.0052689.s004.tif]

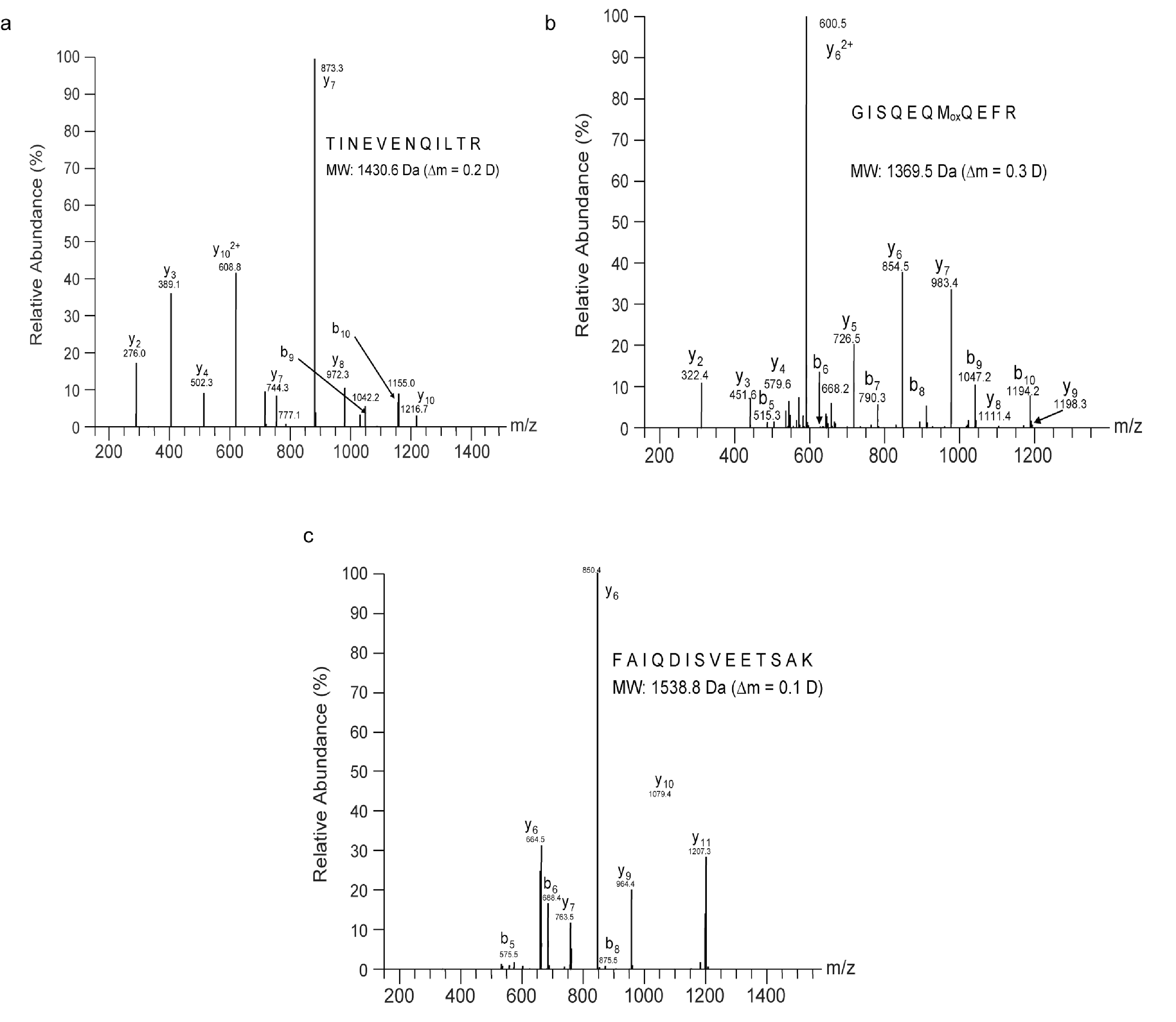

Supplement: Figure S4 — The CID spectra of α-actinin-4 peptides. The chromatin proteome of AT1R-activated cells consisted of peptides (CISQEQMOXQEFR, TINEVENQILTR, FAIQDISVEETSAK) assigned (MASCOT/NCBI non-redundant database) to α-actinin-4. (TIF) [file pone.0052689.s005.tif]

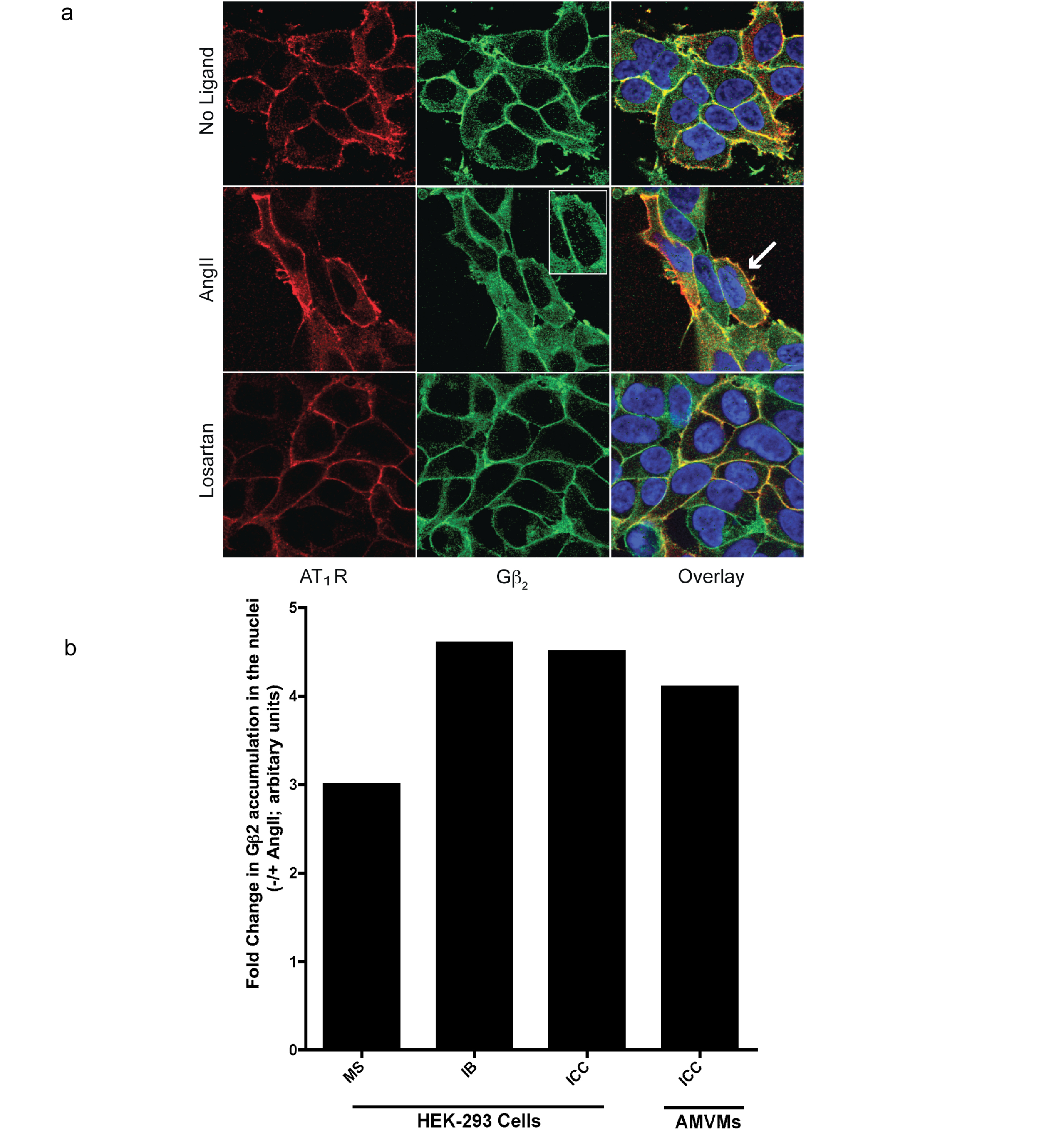

Supplement: Figure S5 — Gβ2 accumulates in the nucleus upon AT1R activation by AngII and is blocked by treatment with the AT1R antagonist, losartan. (a) The HEK-AT1R cells untreated or treated with 1 µM losartan and HA-AT1R were labeled red, and Gβ2 was labeled green. The inset in the right top corner of the middle Gβ2 panel shows a magnified image (1000×) of a single cell (arrow in overlay). The nucleus of the cell shows green staining that corresponds to Gβ2 in the nuclei. (b) Different analytical methods, including mass spectrometry (MS), immunoblot (IB) analysis and immunocytochemistry (ICC), showed equivalent fold changes in Gβ2 accumulation in the nucleus upon AT1R activation. A pixel counting approach estimated (50 cells, n = 3) that ∼30% of the Gβ2 pool was localized in the nucleus when AT1R was activated with 1 µM AngII for 30 min in HASM and HEK-AT1R cells. This distribution accounts for ∼2.5–4.5-fold increases in Gβ2 levels in the nucleus which is similar to that estimated by other methods. (TIF) [file pone.0052689.s006.tif]

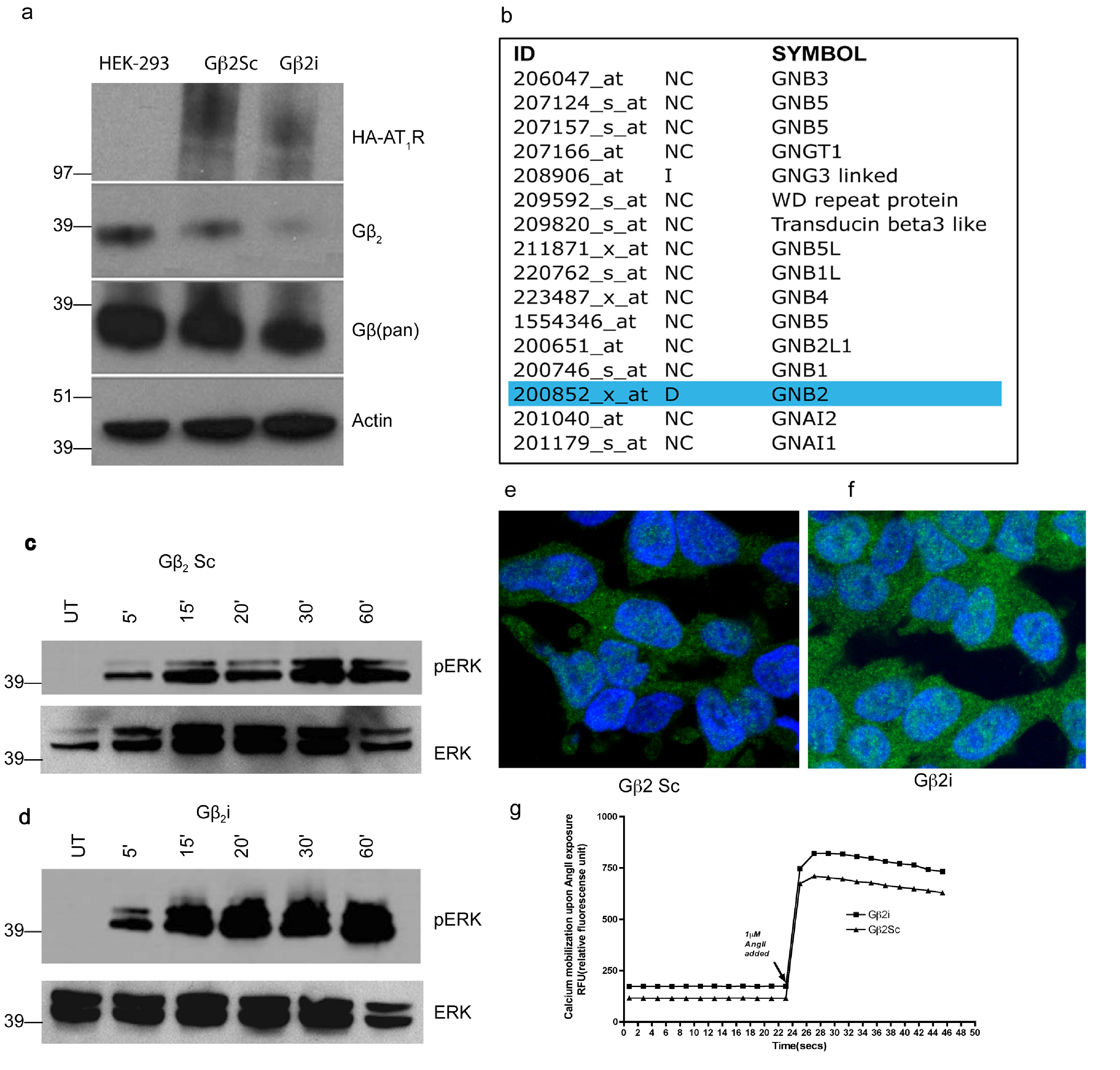

Supplement: Figure S6 — AT1R-mediated cytoplasmic signaling events are unaffected upon RNAi-mediated silencing of Gβ2. (a) Total lysates were prepared from untransfected HEK293 cells, dual plasmid-transfected clones expressing AT1R with scrambled Gβ2-scrambled (Gβ2Sc) and AT1R with a Gβ2RNAi plasmid. Lysates were subjected to immunoblot analysis to detect AT1R expression (anti-HA), Gβ (pan antibody) and β actin (loading control). Both of the cell lines exhibited equivalent levels of AT1R. The Bmax (maximal specific binding) obtained for AT1R-Gβ2Sc was 8.7+/−0.9 pmol/mg and 9.7+/−0.9 pmol.mg for AT1R-Gβ2i with a Kd value of 1732.5+/−170 pM. Taken together, both cell lines expressed comparable levels of AT1R. (b) Table showing the Affymetrix array gene expression data from Gβ2i stable cell lines compared to Gβ2+ cells revealed a knockdown specifically for GNB2. (c–d) Both cell types were serum starved for a minimum of 18 hr and then exposed to vehicle (−) or 1 µM AngII (+) for 5, 10, 15, 20, 30 and 60 min. Lysates were immunoblotted for pERK1/2 and total ERK1/2 in Gβ2Sc and Gβ2i cells. The phosphorylation of ERK1/2 upon AngII activation of AT1R was preserved in the absence of Gβ2. (e–f) Immunocytochemical analysis followed by confocal imaging of pERK1/2 (labeled green) localized in the nuclei (labeled blue with DAPl) in Gβ2Sc and Gβ2i cells upon AT1R activation with AngII. (g) Calcium mobilization upon AngII activation of AT1R was preserved in Gβ2Sc and Gβ2i cells (fluorescence-based assay using FLEX Station 3). (TIF) [file pone.0052689.s007.tif]

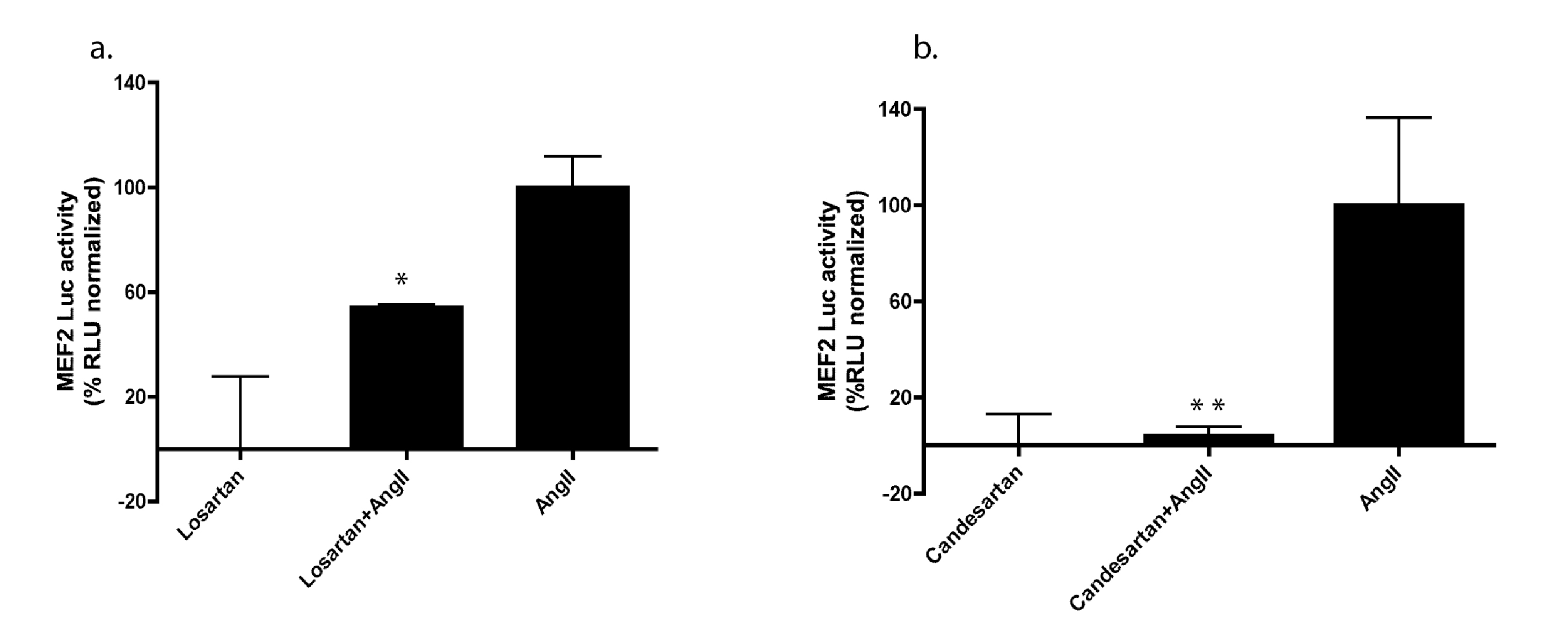

Supplement: Figure S7 — The AT1R blockers, losartan and candesartan, prevented the increase in AngII-mediated MEF2 reporter activity. (a) AngII treatment increased MEF2-luciferase expression, and this increase was blocked by treatment with the AT1R antagonist, losartan (∼54%), and (b) candesartan (∼96%). Note: losartan is a less potent AT1R antagonist compared to candesartan. Data are expressed as % RLU normalized to the AngII response (100%) with losartan/candesartan alone as 0%. Error bars indicate standard error of the mean (n = 3) of experiments performed in duplicate. P values were * = 0.03 and ** = 0.02 using an unpaired t-test (two-tailed with Welch's correction in GraphPad Prism software). (TIF) [file pone.0052689.s008.tif]

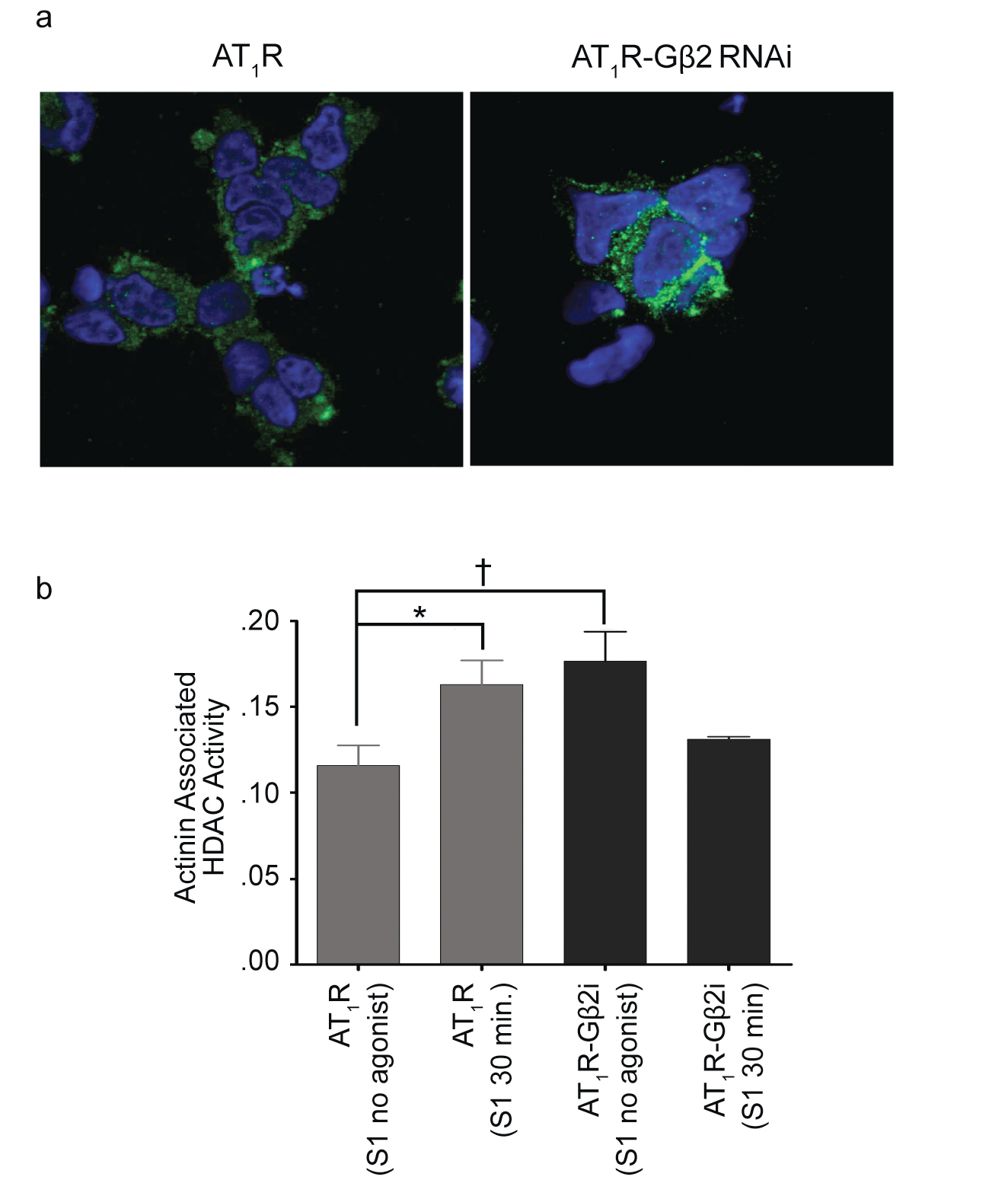

Supplement: Figure S8 — Gβ2 modulates the export of the α-actinin-4-HDAC complex from the nucleus to the cytosol. (a) Immunocytochemical analysis of AT1R and AT1R-Gβ2RNAi cells revealed increased cytoplasmic localization of α-actinin-4 (green) compared with control. (b) An actinin-associated HDAC activity assay on the cytosolic fraction of AT1R in AT1R-Gβ2 RNAi cells (no agonist and AngII 1 µM for 30 min). There was a significant increase in actinin-associated HDAC activity upon AngII treatment of AT1R cells. There was a significant increase under quiescent conditions in Gβ2i cells (no agonist). P value: * = 0.049 and † = 0.034. No significant change was observed upon agonist exposure under conditions of Gβ2 knockdown. (TIF) [file pone.0052689.s009.tif]

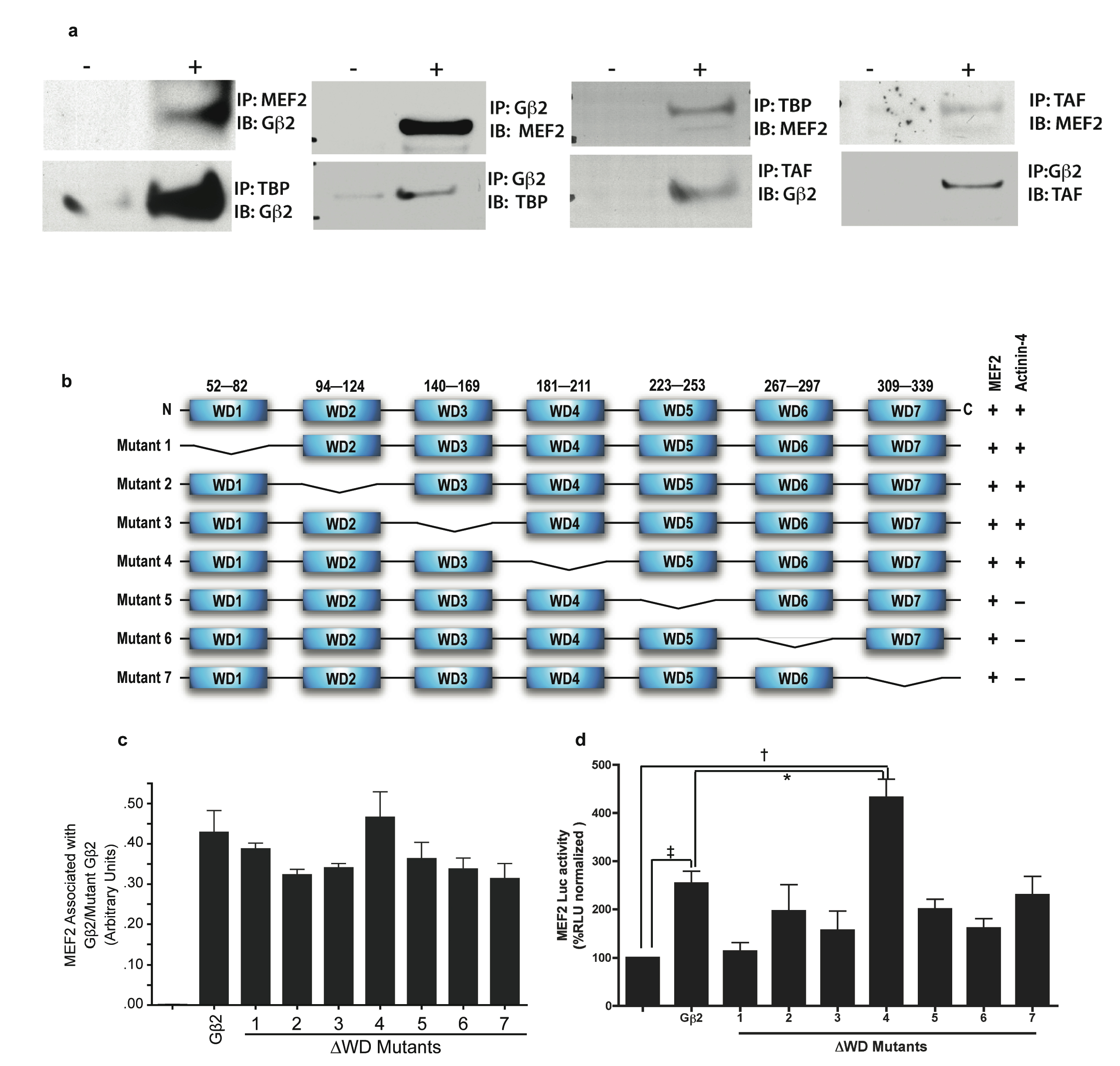

Supplement: Figure S9 — The WD repeats in Gβ2 form a platform to allow for the formation of a multimeric protein complex. Reverse Co-IPs were performed with Prot G (−) and Prot G (+) antibodies. (a) Gβ2, MEF2, TBP and TAF antibodies (+) were used for immunoprecipitation (IP), and the samples were immunoblotted (IB) for interacting proteins as shown here. (b) Schematic representation of sequential WD repeat deletions in Gβ2. (c) Co-immunoprecipitation with anti-M2 FLAG beads in FLAG-Gβ2/mutants and MEF2-expressing cells showed no significant change in MEF2 association with Gβ2 (n = 3). (d) MEF2 functional activity stimulated by Gβ2/WD repeat deletion mutants. Error bars indicate the standard error of the mean (n = 3), and P values were †, ‡ <0.003 and * = 0.011 as calculated using an unpaired t-test (two-tailed) with Welch's correction in GraphPad Prism software. (TIF) [file pone.0052689.s010.tif]

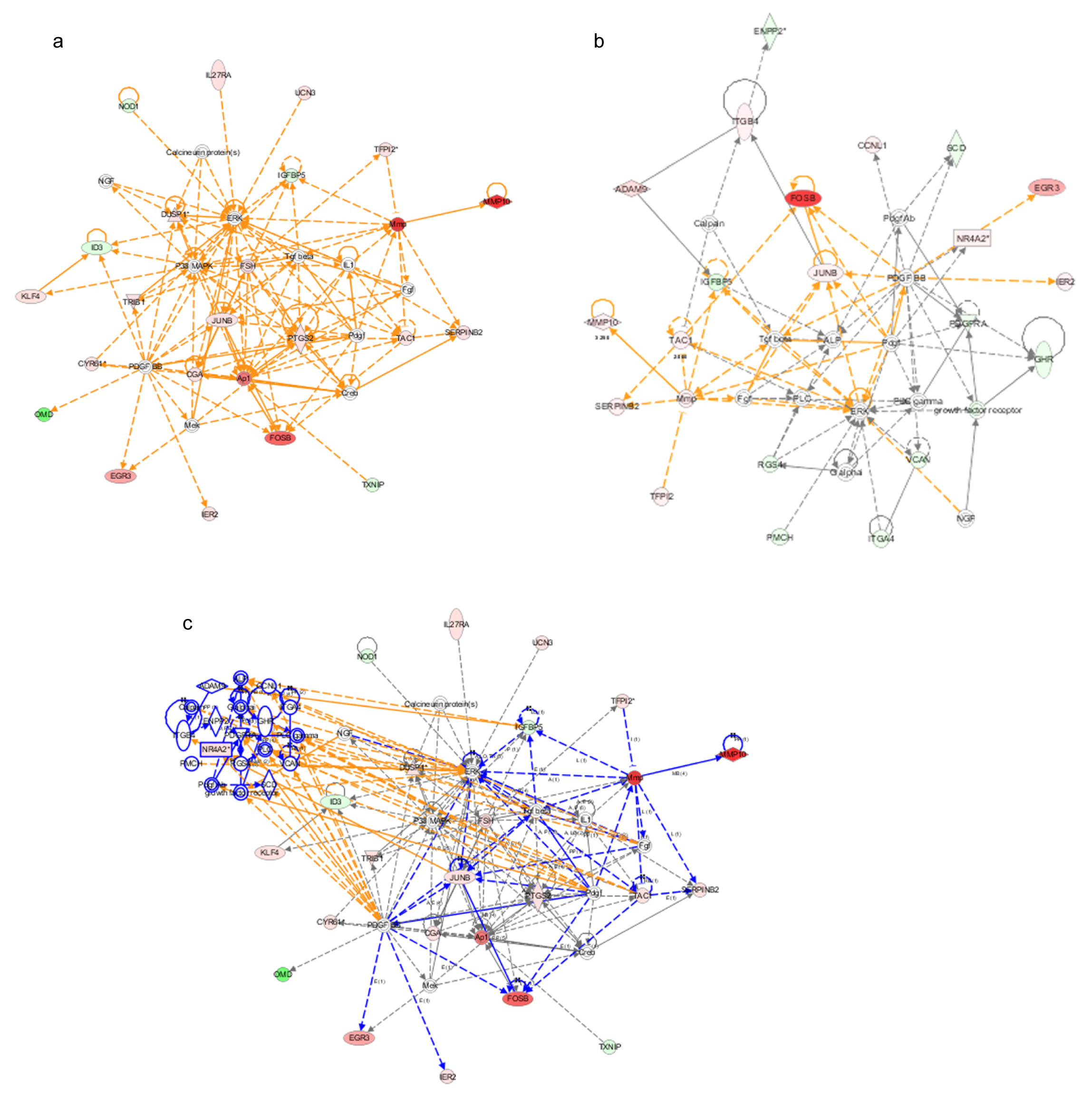

Supplement: Figure S10 — Influence of Gβ2 knockdown on the cellular growth and proliferation network. The knockdown of Gβ2 affected function of this network. (a) The Cellular Growth and Proliferation Network was derived from the IPA analysis of differentially regulated genes in the wild-type cells (in AngII vs. untreated cells). (b) The Cellular Growth and Proliferation Network derived from differentially regulated genes in the Gβ2i cells (in AngII vs. untreated cells). The assigned function for the Cellular Growth and Proliferation Network in Gβ2i cells (i.e., cellular growth and proliferation in connective tissue disorders and in nervous system development and function). (c) The Cellular Growth and Proliferation Network derived from Gβ2i cells is superimposed onto the wild-type network and shows the presence of molecules that now participate in the network and thus assigns it new specialized function. (TIF) [file pone.0052689.s011.tif]
