# Supplementary material for: Interaction of G-Protein βγ Complex with Chromatin Modulates GPCR-Dependent Gene Regulation
Source: PLoS One. 2013 Jan 9;8(1):e52689. doi: 10.1371/journal.pone.0052689 (PMC3541368; doi:10.1371/journal.pone.0052689)
Supplement: Table S1 — The Peptide Index. (DOC) [file pone.0052689.s012.doc]

| **Table S1. The Peptide Index** |  |  |
| --- | --- | --- |
| **Proteins** | **No: of Peptides** | **Peptides Coverage** |
| G-Protein beta 2 (Gβ2) | 4 | 14% |
|  |  |  |
| G-Protein gamma 12 (Gγ12) | 1 | 10% |
|  |  |  |
| α-Actinin4 | 7 | 8% |
|  |  |  |
| Histone H1 | 16 | 45% |
|  |  |  |
| Histone H2B | 11 | 46% |
|  |  |  |
| Histone Core | 5 | 17% |
|  |  |  |
| Histone H4 | 14 | 68% |
|  |  |  |

Peptide index mining showed the presence (number of peptides) and peptide

coverage of histones along with Gβ_2_ , Gγ12 and α-actinin4 in the chromatin

proteome extracted from AT_1_R activated cells.

Note: High abundant proteins in the nucleus have high peptide coverage

when compared to low abundant proteins.
