# Supplementary material for: Interaction of G-Protein βγ Complex with Chromatin Modulates GPCR-Dependent Gene Regulation
Source: PLoS One. 2013 Jan 9;8(1):e52689. doi: 10.1371/journal.pone.0052689 (PMC3541368; doi:10.1371/journal.pone.0052689)
Supplement: Table S2 — Location of Binding Sites for Gβ2 Interacting Transcription Factors within Promoters of Genes Associated with Cellular Growth and Proliferation Network. (DOC) [file pone.0052689.s013.doc]

| **Tables S2. Location of Binding Sites for Gβ_2_ Interacting Transcription Factors within Promoters of Genes Associated with Cellular Growth and Proliferation Network** | | | | | | |  |
| --- | --- | --- | --- | --- | --- | --- | --- |
|  |  |  | **Transcription Factors** | | | | |
| **Gene Name** | **Gene Symbol** | **Fold change^$^** | **MEF2** | **STAT1** | **STAT3** | **NFAT** | |
| ***Genes Leaving Network*** |  |  |  |  |  |  | |
| Jun oncogene | Ap1 | 1.0 | – | – | – | – | |
| Protein phosphatase 3 | Calcineurin proteins | 0.8 | – | – | – | – | |
| Glycoprotein hormone, alpha polypepetide | CGA | 1.3 | – | – | – | – | |
| DNA-binding response regulator CreB | CREB | 1.1 | – | – | – | – | |
| cysteine-rich, angiogenic inducer, 61 | CYR61 | -2.1* | – | – | 1253 | – | |
| dual specificity phosphatase 1 | DUSP1 | -1.2 | – | – | – | – | |
| Follicle stimulating hormone | FSH | 1.1 | – | – | – | – | |
| Inhibitor of DNA binding 3 | ID3 | -1.1 | – | – | – | – | |
| Interleukin1 | IL1 | -2.7* | 1493 | – | – | – | |
| Interleukin receptor 27a | IL27RA | -0.8 | – | – | – | – | |
| Kruppel like factor 4 | KLF4 | -4.9* | 154 | – | – | – | |
| MAP kinse-ERK kinase | MEK | -2.1* | – | 801 | 967 | – | |
| Nucleotide-binding oligomerization domain containing 1 | NOD1 | -3.1* | 207 | 214 | – | – | |
| Osteomodulin | OMD | -2.7* | 1116 | – | 180 | – | |
| Mitogen-activated protein kinase 14 | p38MAPK | 1.0 | – | – | – | – | |
| Prostaglandin-endoperoxide synthase 2 | PTGS2 | -3.6* | 522 | 412 | 1044 | – | |
| Tachykinin, precursor 1 | TAC1 | -2.7* | 848,1177 | – | – | – | |
| Tribbles homolog 1 | TRIB1 | -0.7 | – | – | – | – | |
| Transcription factor | UNC3 |  | NA | NA | NA | NA | |
| ***Genes Entering Network*** |  |  |  |  |  |  | |
| ADAM metallopeptidase domain 9 | ADAM9 | 3.9* | 1259 | – | – | 1271 | |
| Alkaline phosphatase | ALP | 2.3* | 1357 | – | – | – | |
| Calpain | CALPAIN | -1.0 | – | – | – | – | |
| Cyclin L1 | CCNL1 | 2.9* | 23 | 1491 | 1491 | – | |
| G-protein inhibitory alpha subunit | G ALPHAi | 2.1* | – | 858 | – | – | |
| Growth hormone receptor | GHR | 1.0 | – | – | – | – | |
| Growth factor receptor | GFR | -0.8 | – | – | – | – | |
| Integrin, alpha 4 | ITGA4 | 2.3* | 1455 | – | – | – | |
| Integrin, beta 4 | ITGB4 | 2.9* | 133 | – | – | – | |
| Nuclear receptor subfamily 4, group A, member 2 | NR4A2 | 2.3* | 334 | – | – | – | |
| Platelet derived growth factor alpha b | PDGFAB | 2.0* | 375 | – | – | – | |
| Platelet derived growth factor receptor alpha | PDGFRA | 1.1 | – | – | – | – | |
| Phopholipase C | PLC | 2.1* | – | – | 367 | – | |
| Phopholipase C, gamma subunit | PLCGAMMA | -1.2 | – | – | – | – | |
| Pro-melanin-concentrating hormone | PMCH | 1.3 | – | – | – | 18,281 | |
| Regulator of G-protein signaling4 | RGS4 | 0.9 | – | – | – | – | |
| Stearoyl-CoA desaturase | SCD | 1.0 | – | – | – | – | |
| Tachykinin receptor 1 | TACR1 | 3.1* | 7,781,107 | – | – | – | |
| Versican | VCAN | 1.1 | – | – | – | – | |
| Ectonucleotide pyrophosphatase/ phosphodiesterase 2 | ENPP2 | 4.6* | 1288 | – | 70 | – | |
| ^$^Fold change of transcript level estimated, AT_1_R-Gβ_2_ compared to AT_1_R-Gβ_2_-sc treated with 1μM AngII for 60 min. Bioinformatic analysis using Multi-genome Analysis of Positions and Patterns of Elements of Regulation (MAPPER), a platform for the computational identification of transcription factor binding sites (TFBSs). As described in results. 2000 bp region of the promoters of human genes analyzed. The Gβ_2_-knockdown affected participation of genes indicated above in the network, which changes the function. Significant change in expression is indicated by * (p<0.05) | | | | | | |  |
