# Supplementary material for: Interaction of G-Protein βγ Complex with Chromatin Modulates GPCR-Dependent Gene Regulation
Source: PLoS One. 2013 Jan 9;8(1):e52689. doi: 10.1371/journal.pone.0052689 (PMC3541368; doi:10.1371/journal.pone.0052689)
Supplement: Table S3 — Gβ2 Modulates the Expression of Histone and Histone Modifier Genes. (DOC) [file pone.0052689.s014.doc]

| **Table S3. Gβ_2_ Modulates the Expression of Histone and Histone Modifier Genes** | | |
| --- | --- | --- |
| **Gene Name** | **Gene Symbol** | **Fold Change**  **Gβ_2_i vs. Control** |
| Histone cluster 1, H2ac | HIST1H2AC | -2.069517840 |
| Histone cluster 1, H3h | HIST1H3H | -1.609790611 |
| Histone cluster 1, H4h | HIST1H4H | -1.604071967 |
| Histone deacetylase 2 | HDAC2 | -1.570111370 |
| Histone cluster 1, H2bk | HIST1H2BK | -1.533617495 |
| Histone cluster 1, H3bc | HIST1H2BC | 1.602215778 |
| Histone cluster 1, H2bg | HIST1H2BG | 2.263857729 |
| Histone deacetylase 7A | HDAC7A | 2.578063502 |
| -: down regulation. Modulation of expression of histone and histone modifier genes upon Gβ_2_ knockdown. | | |
