# Supplementary material for: Interaction of G-Protein βγ Complex with Chromatin Modulates GPCR-Dependent Gene Regulation
Source: PLoS One. 2013 Jan 9;8(1):e52689. doi: 10.1371/journal.pone.0052689 (PMC3541368; doi:10.1371/journal.pone.0052689)
Supplement: Table S4 — Gβ2 Modulates the Expression of Genes Involved in G-Protein Coupled Receptor Signaling. (DOC) [file pone.0052689.s015.doc]

| **Table S4. Gβ_2_ Modulates the Expression of Genes Involved in G-Protein Coupled Receptor Signaling** | | | |
| --- | --- | --- | --- |
| **Gene Name** | **Gene**  **Symbol** | **Fold Change** |  |
| G-protein coupled receptor 50 | GPR50 | 6.7 |  |
| Cholecystokinin B receptor | CCKBR | 2.2 |  |
| adrenergic, beta-1, receptor | ADRB1 | 2.7 |  |
| Endothelin receptor type B | EDNRB | 6.5 |  |
| Prostaglandin E receptor 4 | PTGER4 | 2.3 |  |
| Proteinase activated receptor-2 | PAR2 | 2.64 |  |
| Coagulation factor II (thrombin) receptor-like 1 | F2RL1 | 1.2 |  |
| G-protein coupled receptor 87 | GPR87 | 4.5 |  |
| G protein-coupled receptor 64 | GPR64 | 1.2 |  |
| G protein-coupled receptor 124 | GPR124 | -1.09 |  |
| Regulator of G-protein signaling 2 | RGS2 | -1.2 |  |
| Protein kinase (cAMP-dependent, catalytic) inhibitor alpha | PKIA | -2.3 |  |
| Mitogen-activated protein kinase kinase 2 | MAP2K2 | -1.09 |  |
| Rho GDP dissociation inhibitor (GDI) alpha | ARHGDIA | -2.00 |  |
| Inositol 1,4,5-triphosphate receptor, type 1 | ITPR1 | -1.09 |  |
| myo-inositol 1-phosphate synthase A1 | ISYNA1 | -1.4 |  |
| Phospholipase A2, group IVA | PLA2 | -1.09 |  |
| Protein kinase (cAMP-dependent, catalytic) inhibitor alpha | PKIA | -1.6 |  |
| phosphodiesterase 4C, cAMP-specific (phosphodiesterase E1 dunce homolog, Drosophila) | PDE4C | -2.9 |  |
| Ras-related C2 botulinum toxin substrate 3  (Rho family, small GTP binding protein Rac3) | RAC3 | -1.6 |  |
| Phosphoinositide-3-kinase, class 2, alpha polypeptide | P13K | 1.09 |  |
| ral guanine nucleotide dissociation stimulator-like 3 | RGL3 | -2.5 |  |
| protein phosphatase 2 (formerly 2A), regulatory subunit A, beta isoform | PPP2R1B | 1.0 |  |
| phospholipase D family, member 5 | PLD5 | 4.4 |  |

- Represents down regulation. Fold change of expression of proteins involved in GPCR signaling upon GB2 knockdown. 2% of modulated genes belong to GPCR function or regulation.
