# Supplementary material for: Interaction of G-Protein βγ Complex with Chromatin Modulates GPCR-Dependent Gene Regulation
Source: PLoS One. 2013 Jan 9;8(1):e52689. doi: 10.1371/journal.pone.0052689 (PMC3541368; doi:10.1371/journal.pone.0052689)
Supplement: Table S5 — Differentially Expressed Genes (p<0.01) Upon Gβ2 Knockdown. (DOC) [file pone.0052689.s016.doc]

| **Table S5.** **Differentially Expressed Genes (p<0.01) Upon Gβ_2_ Knockdown** | | | | | |
| --- | --- | --- | --- | --- | --- |
| **Identifier** | **UniGene ID** | **Gene Name** | **Symbol** | **Log2 Ratio** | **p-value** |
| NM_006820 | Hs.389724 | interferon-induced protein 44-like | IFI44L | 6.79999 | 0.00002 |
| NM_024512 | Hs.657345 | leucine rich repeat containing 2 | LRRC2 | 6.79999 | 0.00035 |
| NM_004224 | Hs.567390 | G protein-coupled receptor 50 | GPR50 | 6.70002 | 0.00002 |
| NM_000115 | Hs.82002 | endothelin receptor type B | EDNRB | 6.50001 | 0.00049 |
| AF154848 | Hs.418062 | beta-1,3-N-acetylgalactosaminyltransferase 1 | B3GALNT1 | 5.89990 | 0.00008 |
| AI822134 | Hs.49889 | poly(A) binding protein, cytoplasmic 4-like | PABPC4L | 5.49985 | 0.00002 |
| BC015907 | Hs.637783 | Homo sapiens, clone IMAGE:3922927, mRNA | --- | 5.39985 | 0.00002 |
| AL137566 | Hs.32405 | progesterone receptor | PGR | 5.39985 | 0.00002 |
| NM_000358 | Hs.369397 | transforming growth factor, beta-induced, 68kDa | TGFBI | 5.30012 | 0.00002 |
| AI123532 | Hs.146542 | neuronal growth regulator 1 | NEGR1 | 5.30012 | 0.00069 |
| R76258 | --- | --- | --- | 5.20006 | 0.00077 |
| AL390170 | Hs.587484 | Clone 23555 mRNA sequence | --- | 4.69988 | 0.00010 |
| AI983428 | Hs.210283 | collagen, type V, alpha 1 | COL5A1 | 4.59991 | 0.00009 |
| NM_023915 | Hs.591292 | G protein-coupled receptor 87 | GPR87 | 4.59991 | 0.00002 |
| AW167298 | Hs.556122 | Clone IMAGE:120162 mRNA sequence | --- | 4.59991 | 0.00003 |
| AA115106 | Hs.146542 | neuronal growth regulator 1 | NEGR1 | 4.59991 | 0.00003 |
| AI804932 | Hs.136247 | protein tyrosine phosphatase-like A domain containing 2 | PTPLAD2 | 4.59991 | 0.00002 |
| AU119545 | Hs.124673 | dermatan sulfate epimerase-like | DSEL | 4.59991 | 0.00004 |
| NM_005532 | Hs.532634 | interferon, alpha-inducible protein 27 | IFI27 | 4.50016 | 0.00002 |
| NM_003991 | Hs.82002 | endothelin receptor type B | EDNRB | 4.50016 | 0.00062 |
| U20428 | Hs.504315 | suppression of tumorigenicity 14 (colon carcinoma) | ST14 | 4.50016 | 0.00017 |
| AK091691 | Hs.672452 | phospholipase D family, member 5 | PLD5 | 4.39985 | 0.00002 |
| AK026736 | Hs.470399 | integrin, beta 6 | ITGB6 | 4.39985 | 0.00002 |
| NM_152711 | --- | --- | --- | 4.30012 | 0.00249 |
| BC001606 | Hs.587558 | neutrophil cytosolic factor 2 | NCF2 | 4.30012 | 0.00002 |
| NM_014031 | Hs.49765 | solute carrier family 27 | SLC27A6 | 4.30012 | 0.00011 |
| AA781795 | Hs.546467 | Epithelial stromal interaction 1 (breast) | EPSTI1 | 4.30012 | 0.0012 |
| BF477544 | Hs.613865 | Transcribed locus | --- | 4.30012 | 0.00004 |
| NM_000443 | Hs.654403 | ATP-binding cassette, sub-family B (MDR/TAP), member 4 | ABCB4 | 4.20006 | 0.00019 |
| NM_021616 | Hs.125300 | tripartite motif-containing 34 /// tripartite motif-containing 6 /// TRIM6-TRIM34 | TRIM34 /// TRIM6 /// TRIM6-TRIM34 | 4.20006 | 0.00008 |
| AI741051 | Hs.496512 | zinc finger, matrin type 1 | ZMAT1 | 4.20006 | 0.00015 |
| BE467023 | Hs.502328 | CD44 molecule (Indian blood group) | CD44 | 4.20006 | 0.00024 |
| AF083130 | Hs.579453 | CATX-14 | --- | 4.20006 | 0.00225 |
| AW189843 | Hs.17518 | radical S-adenosyl methionine domain containing 2 | RSAD2 | 4.20006 | 0.00134 |
| NM_001717 | Hs.459153 | basonuclin 1 | BNC1 | 4.10014 | 0.00006 |
| AY140646 | Hs.136348 | periostin, osteoblast specific factor | POSTN | 4.10014 | 0.00021 |
| BC033535 | Hs.385812 | CDNA clone IMAGE:4822119 | --- | 4.10014 | 0.00249 |
| NM_006183 | Hs.80962 | neurotensin | NTS | 4.10014 | 0.00009 |
| BE551416 | Hs.656805 | alpha-kinase 2 | ALPK2 | 4.10014 | 0.00002 |
| BC005111 | Hs.368538 | glutamate dehydrogenase 2 | GLUD2 | 4.00000 | 0.00134 |
| AK021539 | Hs.124673 | dermatan sulfate epimerase-like | DSEL | 4.00000 | 0.00044 |
| AA234091 | Hs.661436 | Transcribed locus | --- | 4.00000 | 0.00021 |
| AW292830 | Hs.666767 | Transcribed locus | --- | 4.00000 | 0.00003 |
| AI042341 | Hs.646604 | hypothetical protein LOC283481 | LOC283481 | 3.80012 | 0.00002 |
| NM_006588 | Hs.312644 | sulfotransferase family, cytosolic, 1C, member 4 | SULT1C4 | 3.70044 | 0.00011 |
| AK098263 | Hs.659966 | CDNA FLJ40944 fis, clone UTERU2008705 | --- | 3.70044 | 0.00134 |
| NM_005912 | Hs.532833 | melanocortin 4 receptor | MC4R | 3.70044 | 0.00149 |
| N71874 | Hs.163244 | leucine rich repeat neuronal 1 | LRRN1 | 3.70044 | 0.00003 |
| AI816061 | Hs.165636 | DIRAS family, GTP-binding RAS-like 2 | DIRAS2 | 3.70044 | 0.00021 |
| NM_004335 | Hs.118110 | bone marrow stromal cell antigen 2 | BST2 | 3.60051 | 0.00002 |
| AB050855 | Hs.418062 | beta-1,3-N-acetylgalactosaminyltransferase 1 | B3GALNT1 | 3.60051 | 0.00002 |
| BC014155 | Hs.159013 | Ras homolog enriched in brain like 1 | RHEBL1 | 3.49953 | 0.00069 |
| NM_006017 | Hs.614734 | prominin 1 | PROM1 | 3.49953 | 0.00002 |
| NM_022873 | Hs.511731 | interferon, alpha-inducible protein 6 | IFI6 | 3.49953 | 0.00003 |
| NM_000422 | Hs.2785 | keratin 17 | KRT17 | 3.49953 | 0.00002 |
| AB050856 | Hs.418062 | beta-1,3-N-acetylgalactosaminyltransferase 1 | B3GALNT1 | 3.40054 | 0.00035 |
| BC017958 | Hs.679431 | hypothetical gene supported by BC017958 | LOC347475 | 3.30012 | 0.00049 |
| Z19574 | Hs.2785 | keratin 17 | KRT17 | 3.30012 | 0.00002 |
| BG285837 | Hs.537370 | Clone HLS_IMAGE_626842 mRNA sequence | --- | 3.30012 | 0.00002 |
| BC002642 | Hs.181301 | cathepsin S | CTSS | 3.20006 | 0.00120 |
| AI420977 | Hs.335933 | V-set and transmembrane domain containing 2A | VSTM2A | 3.20006 | 0.00055 |
| AW242836 | Hs.504301 | transmembrane protein 45B | TMEM45B | 3.20006 | 0.00004 |
| NM_152632 | Hs.680415 | chromosome X open reading frame 22 | CXorf22 | 3.09930 | 0.00077 |
| U77917 | Hs.506076 | protein tyrosine phosphatase, receptor type, R | PTPRR | 3.09930 | 0.00015 |
| NM_006417 | Hs.82316 | interferon-induced protein 44 | IFI44 | 3.09930 | 0.00183 |
| BE502910 | Hs.5025 | nebulette | NEBL | 3.09930 | 0.00015 |
| AI247495 | Hs.685527 | Transcribed locus | --- | 3.09930 | 0.00097 |
| AL157448 | Hs.675505 | MRNA; cDNA DKFZp761E0311 (from clone DKFZp761E0311) | --- | 3.00000 | 0.00010 |
| NM_002345 | Hs.406475 | lumican | LUM | 3.00000 | 0.00002 |
| U25804 | Hs.138378 | caspase 4, apoptosis-related cysteine peptidase | CASP4 | 3.00000 | 0.00009 |
| H17038 | Hs.126856 | similar to CG4502-PA | FLJ25076 | 3.00000 | 0.00002 |
| AV726956 | Hs.47209 | BEX family member 5 | BEX5 | 3.00000 | 0.00031 |
| NM_003641 | Hs.458414 | interferon induced transmembrane protein 1 (9-27) | IFITM1 | 2.89918 | 0.00002 |
| AB030655 | Hs.647231 | EGF-containing fibulin-like extracellular matrix protein 2 | EFEMP2 | 2.89918 | 0.00003 |
| BC003096 | Hs.424312 | PDZ and LIM domain 4 | PDLIM4 | 2.89918 | 0.00027 |
| BF338947 | Hs.374650 | interferon induced transmembrane protein 3 (1-8U) | IFITM3 | 2.89918 | 0.00002 |
| NM_018476 | Hs.334370 | brain expressed, X-linked 1 | BEX1 | 2.89918 | 0.00002 |
| AW296421 | Hs.650577 | Transcribed locus | --- | 2.89918 | 0.00087 |
| BE552137 | Hs.370303 | zinc finger protein 366 | ZNF366 | 2.89918 | 0.00183 |
| AA749101 | Hs.458414 | interferon induced transmembrane protein 1 (9-27) | IFITM1 | 2.79909 | 0.00003 |
| AI816281 | Hs.700718 | CDNA FLJ42233 fis, clone THYMU3000420 | --- | 2.79909 | 0.00225 |
| BC017854 | Hs.436298 | epithelial membrane protein 1 | EMP1 | 2.70044 | 0.00108 |
| AW972359 | Hs.663352 | Transcribed locus | --- | 2.70044 | 0.00035 |
| AI625747 | Hs.99913 | adrenergic, beta-1-, receptor | ADRB1 | 2.70044 | 0.00013 |
| AA991551 | Hs.97013 | chromosome 11 open reading frame 52 | C11orf52 | 2.70044 | 0.00021 |
| NM_033128 | Hs.655515 | scinderin | SCIN | 2.59932 | 0.00002 |
| BC020672 | Hs.529509 | natural killer-tumor recognition sequence | NKTR | 2.59932 | 0.00249 |
| NM_014782 | Hs.48924 | armadillo repeat containing, X-linked 2 | ARMCX2 | 2.59932 | 0.00002 |
| AF043337 | Hs.551925 | interleukin 8 | IL8 | 2.59932 | 0.00017 |
| AJ276395 | Hs.203717 | fibronectin 1 | FN1 | 2.59932 | 0.00015 |
| L16895 | Hs.102267 | lysyl oxidase | LOX | 2.59932 | 0.00002 |
| AF228422 | Hs.112242 | chromosome 15 open reading frame 48 | C15orf48 | 2.59932 | 0.00077 |
| AW450397 | Hs.422967 | CDNA FLJ90099 fis, clone HEMBA1006016 | --- | 2.59932 | 0.00097 |
| BG149530 | Hs.122561 | vitelline membrane outer layer 1 homolog (chicken) | VMO1 | 2.59932 | 0.00203 |
| NM_145013 | Hs.351133 | chromosome 11 open reading frame 45 | C11orf45 | 2.50080 | 0.00249 |
| NM_005562 | Hs.591484 | laminin, gamma 2 | LAMC2 | 2.50080 | 0.00003 |
| NM_005101 | Hs.458485 | ISG15 ubiquitin-like modifier | ISG15 | 2.50080 | 0.00002 |
| X02761 | Hs.203717 | fibronectin 1 | FN1 | 2.50080 | 0.00002 |
| AK026737 | Hs.203717 | fibronectin 1 | FN1 | 2.50080 | 0.00002 |
| BC000680 | Hs.533626 | tRNA selenocysteine associated protein 1 | TRSPAP1 | 2.50080 | 0.00002 |
| BC001256 | Hs.272299 | chromosome 1 open reading frame 91 | C1orf91 | 2.50080 | 0.00165 |
| AW572911 | Hs.126856 | similar to CG4502-PA | FLJ25076 | 2.50080 | 0.00003 |
| AF276507 | Hs.655515 | scinderin | SCIN | 2.40054 | 0.00002 |
| AV731490 | Hs.310545 | synaptotagmin I | SYT1 | 2.40054 | 0.00003 |
| NM_002527 | Hs.99171 | neurotrophin 3 | NTF3 | 2.40054 | 0.00009 |
| M92934 | Hs.591346 | connective tissue growth factor | CTGF | 2.40054 | 0.00002 |
| M19154 | Hs.133379 | transforming growth factor, beta 2 | TGFB2 | 2.40054 | 0.00005 |
| BC005858 | Hs.203717 | fibronectin 1 | FN1 | 2.40054 | 0.00002 |
| AJ276395 | Hs.203717 | fibronectin 1 | FN1 | 2.40054 | 0.00002 |
| AA584310 | Hs.405614 | collagen triple helix repeat containing 1 | CTHRC1 | 2.40054 | 0.00002 |
| BG429893 | Hs.153408 | CDNA FLJ10570 fis, clone NT2RP2003117 | --- | 2.40054 | 0.00134 |
| AA054125 | --- | --- | --- | 2.40054 | 0.00019 |
| AI022632 | Hs.602954 | Transcribed locus | --- | 2.40054 | 0.00225 |
| BC017314 | Hs.369438 | v-ets erythroblastosis virus E26 oncogene homolog 1 (avian) | ETS1 | 2.29866 | 0.00007 |
| AI375915 | --- | --- | --- | 2.29866 | 0.00097 |
| NM_003246 | Hs.164226 | thrombospondin 1 | THBS1 | 2.29866 | 0.00031 |
| NM_000094 | Hs.476218 | collagen, type VII, alpha 1 ( | COL7A1 | 2.29866 | 0.00004 |
| AA897516 | Hs.199248 | prostaglandin E receptor 4 | PTGER4 | 2.29866 | 0.00002 |
| AB018580 | Hs.78183 | aldo-keto reductase family 1, member C3 | AKR1C3 | 2.29866 | 0.00002 |
| BC000740 | Hs.203 | cholecystokinin B receptor | CCKBR | 2.29866 | 0.00149 |
| NM_022168 | Hs.163173 | interferon induced with helicase C domain 1 | IFIH1 | 2.29866 | 0.00007 |
| AI282982 | Hs.504301 | transmembrane protein 45B | TMEM45B | 2.29866 | 0.00002 |
| H23551 | Hs.655757 | Primary neuroblastoma cDNA, clone:Nbla04246, full insert sequence | --- | 2.29866 | 0.00004 |
| NM_000693 | Hs.459538 | aldehyde dehydrogenase 1 family, member A3 | ALDH1A3 | 2.19849 | 0.00002 |
| AF130095 | Hs.203717 | fibronectin 1 | FN1 | 2.19849 | 0.00002 |
| N30339 | Hs.210283 | collagen, type V, alpha 1 | COL5A1 | 2.19849 | 0.00002 |
| AW004016 | Hs.709275 | ST6 beta-galactosamide alpha-2,6-sialyltranferase 2 | ST6GAL2 | 2.19849 | 0.00062 |
| AI797276 | Hs.591481 | Src homology 2 domain containing E | SHE | 2.19849 | 0.00203 |
| AI744280 | Hs.536395 | Hypothetical LOC642477 | FLJ39632 | 2.19849 | 0.00031 |
| BC035170 | Hs.335239 | ankyrin repeat domain 28 | ANKRD28 | 2.10098 | 0.00087 |
| NM_021076 | Hs.198760 | neurofilament, heavy polypeptide 200kDa | NEFH | 2.10098 | 0.00008 |
| BC005961 | Hs.591159 | parathyroid hormone-like hormone | PTHLH | 2.10098 | 0.00002 |
| BF342851 | Hs.332197 | peroxidasin homolog (Drosophila) | PXDN | 2.10098 | 0.00005 |
| AF070581 | Hs.655757 | Primary neuroblastoma cDNA, | --- | 2.10098 | 0.00002 |
| BF001941 | Hs.487471 | RNA binding motif protein 35A | RBM35A | 2.10098 | 0.00002 |
| AI742057 | Hs.7155 | cytidine monophosphate | CMPK2 | 2.10098 | 0.00011 |
| AL162069 | Hs.140978 | keratin 80 | KRT80 | 2.10098 | 0.00005 |
| AL530703 | Hs.536395 | hypothetical LOC642477 /// similar to double homeobox A | FLJ39632 /// LOC100131139 | 2.10098 | 0.00004 |
| AV729557 | Hs.656225 | Transcribed locus | --- | 2.10098 | 0.00009 |
| NM_153262 | Hs.658866 | synaptotagmin XIV | SYT14 | 2.00000 | 0.00149 |
| NM_021173 | Hs.523829 | polymerase (DNA-directed), delta 4 | POLD4 | 2.00000 | 0.00015 |
| NM_004030 | Hs.166120 | interferon regulatory factor 7 | IRF7 | 2.00000 | 0.00002 |
| AI493245 | Hs.502328 | CD44 molecule | CD44 | 2.00000 | 0.00002 |
| AC005378 | Hs.655684 | contactin associated protein-like 2 | CNTNAP2 | 2.00000 | 0.00149 |
| NM_022350 | Hs.591249 | endoplasmic reticulum aminopeptidase 2 | ERAP2 | 2.00000 | 0.00002 |
| AL035541 | Hs.517155 | prostate transmembrane protein, androgen induced 1 | PMEPA1 | 2.00000 | 0.00002 |
| BC006128 | Hs.98328 | chromosome 11 open reading frame 70 | C11orf70 | 2.00000 | 0.00002 |
| AA633203 | Hs.546467 | epithelial stromal interaction 1 (breast) | EPSTI1 | 2.00000 | 0.00009 |
| AI675836 | Hs.591915 | sortilin-related VPS10 domain containing receptor 1 | SORCS1 | 2.00000 | 0.00203 |
| AI272825 | Hs.595458 | microtubule associated serine/threonine kinase family member 4 /// hypothetical protein LOC100128443 | LOC100128443 /// MAST4 | 2.00000 | 0.00249 |
| BC029890 | Hs.463110 | hypothetical LOC653110 /// hypothetical protein LOC728449 /// hypothetical protein LOC100132646 | LOC100132646 /// LOC653110 /// LOC728449 | 1.89918 | 0.00019 |
| BF111821 | Hs.446017 | WD repeat and SOCS box-containing 1 | WSB1 | 1.89918 | 0.00009 |
| NM_005103 | Hs.224008 | fasciculation and elongation protein zeta 1 (zygin I) | FEZ1 | 1.89918 | 0.00002 |
| U91903 | Hs.128453 | frizzled-related protein | FRZB | 1.89918 | 0.00077 |
| NM_021822 | Hs.660143 | apolipoprotein B mRNA editing enzyme, catalytic polypeptide-like 3G /// apolipoprotein B mRNA editing enzyme, catalytic polypeptide-like 3F | APOBEC3F /// APOBEC3G | 1.89918 | 0.00002 |
| NM_021992 | Hs.56145 | thymosin-like 8 /// thymosin beta15b | MGC39900 /// TMSL8 | 1.89918 | 0.00027 |
| NM_005414 | Hs.581632 | SKI-like oncogene | SKIL | 1.89918 | 0.00165 |
| NM_002506 | Hs.2561 | nerve growth factor (beta polypeptide) | NGF | 1.89918 | 0.00027 |
| BC001234 | Hs.152944 | loss of heterozygosity, 11, chromosomal region 2, gene A | LOH11CR2A | 1.89918 | 0.00002 |
| D86983 | Hs.332197 | peroxidasin homolog (Drosophila) | PXDN | 1.89918 | 0.00077 |
| BF508948 | Hs.660143 | apolipoprotein B mRNA editing enzyme, catalytic polypeptide-like 3G /// apolipoprotein B mRNA editing enzyme, catalytic polypeptide-like 3F | APOBEC3F /// APOBEC3G | 1.89918 | 0.00002 |
| NM_017697 | Hs.487471 | RNA binding motif protein 35A | RBM35A | 1.89918 | 0.00002 |
| NM_020152 | Hs.222802 | chromosome 21 open reading frame 7 | C21orf7 | 1.89918 | 0.00013 |
| AK025651 | --- | --- | --- | 1.89918 | 0.00024 |
| BF591534 | Hs.21861 | transcription elongation factor A (SII)-like 7 | TCEAL7 | 1.89918 | 0.00002 |
| AV739182 | Hs.688696 | Transcribed locus | --- | 1.89918 | 0.00019 |
| X15306 | Hs.198760 | neurofilament, heavy polypeptide 200kDa | NEFH | 1.89918 | 0.00000 |
| AI424872 | Hs.678758 | Homo sapiens, clone IMAGE:4271781 | --- | 1.89918 | 0.00061 |
| NM_021978 | Hs.504315 | suppression of tumorigenicity 14 (colon carcinoma) | ST14 | 1.79909 | 0.00044 |
| M27968 | Hs.284244 | fibroblast growth factor 2 (basic) | FGF2 | 1.79909 | 0.00108 |
| M24915 | Hs.502328 | CD44 molecule (Indian blood group) | CD44 | 1.79909 | 0.00002 |
| NM_003186 | Hs.632099 | transgelin | TAGLN | 1.79909 | 0.00010 |
| AW084068 | Hs.507680 | NEDD4 binding protein 2-like 2 | N4BP2L2 | 1.79909 | 0.00002 |
| NM_022142 | Hs.104894 | epididymal sperm binding protein 1 | ELSPBP1 | 1.79909 | 0.00165 |
| BE218980 | Hs.369438 | v-ets erythroblastosis virus E26 oncogene homolog 1 (avian) | ETS1 | 1.79909 | 0.00002 |
| AK026748 | Hs.91521 | hypothetical LOC54492 | LOC54492 | 1.79909 | 0.00002 |
| AI754423 | Hs.435013 | vestigial like 3 (Drosophila) | VGLL3 | 1.79909 | 0.00002 |
| AI418293 | Hs.444075 | ubiquitin associated and SH3 domain containing, B | UBASH3B | 1.79909 | 0.00002 |
| AI871160 | --- | --- | --- | 1.79909 | 0.00062 |
| AI685841 | Hs.161354 | Transcribed locus | --- | 1.79909 | 0.0001 |
| BE738279 | Hs.536395 | hypothetical LOC642477 | FLJ39632 | 1.79909 | 0.00049 |
| BC017927 | Hs.591249 | endoplasmic reticulum aminopeptidase 2 | ERAP2 | 1.70044 | 0.00024 |
| NM_005860 | Hs.529038 | follistatin-like 3 (secreted glycoprotein) | FSTL3 | 1.70044 | 0.00013 |
| NM_014391 | Hs.448589 | ankyrin repeat domain 1 (cardiac muscle) | ANKRD1 | 1.70044 | 0.00035 |
| NM_002381 | Hs.656199 | matrilin 3 | MATN3 | 1.70044 | 0.00039 |
| NM_018891 | Hs.591484 | laminin, gamma 2 | LAMC2 | 1.70044 | 0.00087 |
| BC004372 | Hs.502328 | CD44 molecule (Indian blood group) | CD44 | 1.70044 | 0.00002 |
| U37546 | Hs.127799 | baculoviral IAP repeat-containing 3 | BIRC3 | 1.70044 | 0.00087 |
| AL050153 | --- | --- | --- | 1.70044 | 0.00127 |
| NM_022772 | Hs.55016 | EPS8-like 2 | EPS8L2 | 1.70044 | 0.00019 |
| NM_022074 | Hs.150651 | family with sequence similarity 111, member A | FAM111A | 1.70044 | 0.00003 |
| AL035541 | Hs.517155 | prostate transmembrane protein, androgen induced 1 | PMEPA1 | 1.70044 | 0.00005 |
| AA131041 | Hs.437609 | interferon-induced protein with tetratricopeptide repeats 2 | IFIT2 | 1.70044 | 0.00097 |
| AI475544 | Hs.642877 | Metastasis associated lung adenocarcinoma transcript 1 (non-protein coding) | MALAT1 | 1.70044 | 0.00002 |
| AV723914 | Hs.632788 | family with sequence similarity 19 (chemokine (C-C motif)-like), member A5 | FAM19A5 | 1.70044 | 0.00077 |
| AI761675 | --- | --- | --- | 1.70044 | 0.00002 |
| BF059748 | Hs.519884 | glucosaminyl (N-acetyl) transferase 2, I-branching enzyme (I blood group) | GCNT2 | 1.70044 | 0.00007 |
| BG292389 | Hs.591249 | endoplasmic reticulum aminopeptidase 2 | ERAP2 | 1.70044 | 0.00049 |
| AW195867 | --- | --- | --- | 1.70044 | 0.00134 |
| BE783098 | Hs.467759 | neuroblastoma-amplified protein | NAG | 1.70044 | 0.00134 |
| N23651 | Hs.435719 | sidekick homolog 2 (chicken) | SDK2 | 1.70044 | 0.00055 |
| AA765841 | Hs.610345 | Transcribed locus | --- | 1.70044 | 0.00225 |
| AI217992 | Hs.164162 | pleckstrin homology domain containing, family H (with MyTH4 domain) member 2 | PLEKHH2 | 1.70044 | 0.0012 |
| BC046208 | Hs.163173 | interferon induced with helicase C domain 1 | IFIH1 | 1.59932 | 0.00069 |
| BE221212 | Hs.172928 | collagen, type I, alpha 1 | COL1A1 | 1.59932 | 0.00002 |
| BC037944 | Hs.685021 | CDNA clone IMAGE:5285703 | --- | 1.59932 | 0.00249 |
| NM_002402 | Hs.270978 | mesoderm specific transcript homolog (mouse) | MEST | 1.59932 | 0.00002 |
| NM_005655 | Hs.435001 | Kruppel-like factor 10 | KLF10 | 1.59932 | 0.00002 |
| NM_005356 | Hs.470627 | lymphocyte-specific protein tyrosine kinase | LCK | 1.59932 | 0.00007 |
| NM_003726 | Hs.316931 | src kinase associated phosphoprotein 1 | SKAP1 | 1.59932 | 0.00004 |
| NM_018962 | Hs.254560 | Down syndrome critical region gene 6 | DSCR6 | 1.59932 | 0.00003 |
| Y13786 | Hs.483944 | ADAM metallopeptidase domain 19 (meltrin beta) | ADAM19 | 1.59932 | 0.00003 |
| BE903880 | Hs.502328 | CD44 molecule (Indian blood group) | CD44 | 1.59932 | 0.00002 |
| BE965029 | Hs.501928 | microtubule associated monoxygenase, calponin and LIM domain containing 2 | MICAL2 | 1.59932 | 0.00017 |
| NM_006187 | Hs.528634 | 2'-5'-oligoadenylate synthetase 3, 100kDa | OAS3 | 1.59932 | 0.00021 |
| AW138743 | Hs.501106 | actin filament associated protein 1-like 2 | AFAP1L2 | 1.59932 | 0.00008 |
| BE889628 | Hs.482910 | Transcribed locus | --- | 1.59932 | 0.00007 |
| BG481877 | Hs.414740 | B-cell CLL/lymphoma 9-like | BCL9L | 1.59932 | 0.00108 |
| BE856341 | Hs.503831 | layilin | LAYN | 1.59932 | 0.00002 |
| AI075407 | Hs.47338 | interferon-induced protein with tetratricopeptide repeats 3 | IFIT3 | 1.59932 | 0.00002 |
| AW025358 | Hs.594352 | Transcribed locus | --- | 1.59932 | 0.00134 |
| AK026836 | --- | --- | --- | 1.59932 | 0.00108 |
| AL049245 | Hs.638732 | MRNA; cDNA DKFZp564C203 (from clone DKFZp564C203) | --- | 1.50080 | 0.00015 |
| NM_000584 | Hs.551925 | interleukin 8 | IL8 | 1.50080 | 0.00002 |
| NM_004431 | Hs.171596 | EPH receptor A2 | EPHA2 | 1.50080 | 0.00002 |
| NM_002317 | Hs.102267 | lysyl oxidase | LOX | 1.50080 | 0.00003 |
| NM_001159 | Hs.406238 | aldehyde oxidase 1 | AOX1 | 1.50080 | 0.00024 |
| NM_012323 | Hs.517617 | v-maf musculoaponeurotic fibrosarcoma oncogene homolog F (avian) | MAFF | 1.50080 | 0.00002 |
| NM_000585 | Hs.654378 | interleukin 15 | IL15 | 1.50080 | 0.00002 |
| NM_000366 | Hs.133892 | tropomyosin 1 (alpha) | TPM1 | 1.50080 | 0.00021 |
| BG327863 | Hs.694721 | CD24 molecule | CD24 | 1.50080 | 0.00039 |
| AF081583 | Hs.445489 | pleckstrin homology domain containing, family B (evectins) member 1 | PLEKHB1 | 1.50080 | 0.00108 |
| AB029032 | Hs.408142 | KIAA1109 | KIAA1109 | 1.50080 | 0.00087 |
| AU148057 | Hs.292156 | dickkopf homolog 3 (Xenopus laevis) | DKK3 | 1.50080 | 0.00002 |
| NM_016639 | Hs.355899 | tumor necrosis factor receptor superfamily, member 12A | TNFRSF12A | 1.50080 | 0.00002 |
| AF154054 | Hs.40098 | gremlin 1, cysteine knot superfamily, homolog (Xenopus laevis) | GREM1 | 1.50080 | 0.00009 |
| NM_016206 | Hs.435013 | vestigial like 3 (Drosophila) | VGLL3 | 1.50080 | 0.00087 |
| AI042152 | --- | trophoblast-derived noncoding RNA | TncRNA | 1.50080 | 0.00003 |
| BF674052 | --- | microRNA 21 | MIRN21 | 1.50080 | 0.00002 |
| N21426 | Hs.369520 | synaptotagmin-like 2 | SYTL2 | 1.50080 | 0.00002 |
| AA532718 | Hs.662933 | Transcribed locus | --- | 1.50080 | 0.00024 |
| BF681162 | Hs.349306 | ring finger protein 145 | RNF145 | 1.50080 | 0.00049 |
| BF062399 | --- | --- | --- | 1.50080 | 0.00183 |
| AA910946 | Hs.18894 | adaptor-related protein complex 1, mu 2 subunit | AP1M2 | 1.50080 | 0.00000 |
| BC040579 | Hs.434703 | CDNA clone IMAGE:5268080 | --- | 1.40054 | 0.00039 |
| AF085995 | Hs.709491 | Hypothetical LOC645513 | LOC645513 | 1.40054 | 0.00203 |
| NM_000611 | Hs.278573 | CD59 molecule, complement regulatory protein | CD59 | 1.40054 | 0.00002 |
| NM_001423 | Hs.436298 | epithelial membrane protein 1 | EMP1 | 1.40054 | 0.00002 |
| NM_016201 | Hs.709272 | angiomotin like 2 | AMOTL2 | 1.40054 | 0.00031 |
| NM_001197 | Hs.475055 | BCL2-interacting killer (apoptosis-inducing) | BIK | 1.40054 | 0.00249 |
| NM_018393 | Hs.655341 | t-complex 11 (mouse)-like 1 | TCP11L1 | 1.40054 | 0.00165 |
| NM_005242 | Hs.154299 | coagulation factor II (thrombin) receptor-like 1 | F2RL1 | 1.40054 | 0.00002 |
| NM_003182 | Hs.2563 | tachykinin, precursor 1 | TAC1 | 1.40054 | 0.00002 |
| NM_001083 | Hs.647971 | phosphodiesterase 5A, cGMP-specific | PDE5A | 1.40054 | 0.00008 |
| NM_004936 | Hs.72901 | cyclin-dependent kinase inhibitor 2B (p15, inhibits CDK4) | CDKN2B | 1.40054 | 0.00097 |
| NM_001458 | Hs.58414 | filamin C, gamma (actin binding protein 280) | FLNC | 1.40054 | 0.00021 |
| AF087853 | Hs.110571 | growth arrest and DNA-damage-inducible, beta | GADD45B | 1.40054 | 0.00003 |
| AF078077 | Hs.110571 | growth arrest and DNA-damage-inducible, beta | GADD45B | 1.40054 | 0.00002 |
| BC001387 | Hs.502775 | HRAS-like suppressor 3 | HRASLS3 | 1.40054 | 0.00002 |
| AI814551 | Hs.264 | patatin-like phospholipase domain containing 4 | PNPLA4 | 1.40054 | 0.00007 |
| AF097495 | Hs.116448 | glutaminase | GLS | 1.40054 | 0.00010 |
| AJ002077 | Hs.180711 | syntaxin 3 | STX3 | 1.40054 | 0.00002 |
| AI087937 | Hs.475502 | transmembrane protein 40 | TMEM40 | 1.40054 | 0.00019 |
| AL136680 | --- | guanylate binding protein 3 | GBP3 | 1.40054 | 0.00087 |
| AI986239 | Hs.655602 | AHA1, activator of heat shock 90kDa protein ATPase homolog 2 (yeast) | AHSA2 | 1.40054 | 0.00003 |
| AI984607 | Hs.186810 | RALBP1 associated Eps domain containing 2 | REPS2 | 1.40054 | 0.00002 |
| AI110850 | Hs.23439 | chromosome 4 open reading frame 32 | C4orf32 | 1.40054 | 0.00002 |
| AA741307 | Hs.65641 | sterile alpha motif domain containing 9 | SAMD9 | 1.40054 | 0.00004 |
| AW139393 | Hs.710069 | hypothetical protein LOC100130938 | LOC100130938 | 1.40054 | 0.00120 |
| AI827906 | Hs.192877 | hypothetical protein LOC169834 | LOC169834 | 1.40054 | 0.00039 |
| AI765540 | Hs.548045 | Transcribed locus | --- | 1.40054 | 0.00011 |
| H04388 | Hs.651324 | hypothetical gene supported by BX538329 | DKFZp686L14188 | 1.40054 | 0.00165 |
| AC009227 | Hs.470277 | UDP-N-acetyl-alpha-D-galactosamine:polypeptide N-acetylgalactosaminyltransferase 13 (GalNAc-T13) | GALNT13 | 1.40054 | 0.00249 |
| AW293849 | Hs.659259 | Homo sapiens, clone IMAGE:5218412, mRNA | --- | 1.40054 | 0.00002 |
| AI440266 | Hs.170673 | epidermal retinal dehydrogenase 2 | RDHE2 | 1.40054 | 0.00044 |
| AA521463 | Hs.632527 | Solute carrier family 35, member F5 | SLC35F5 | 1.40054 | 0.00134 |
| BE645435 | Hs.98328 | chromosome 11 open reading frame 70 | C11orf70 | 1.40054 | 0.00009 |
| BC044938 | Hs.591275 | F-box and leucine-rich repeat protein 21 | FBXL21 | 1.29866 | 0.00013 |
| BQ183759 | Hs.662457 | Hypothetical protein LOC149478 | LOC149478 | 1.29866 | 0.00120 |
| AL552534 | Hs.502328 | CD44 molecule (Indian blood group) | CD44 | 1.29866 | 0.00002 |
| NM_002705 | Hs.192233 | periplakin | PPL | 1.29866 | 0.00013 |
| NM_006115 | Hs.30743 | preferentially expressed antigen in melanoma | PRAME | 1.29866 | 0.00225 |
| NM_002006 | Hs.284244 | fibroblast growth factor 2 (basic) | FGF2 | 1.29866 | 0.00024 |
| NM_004726 | Hs.186810 | RALBP1 associated Eps domain containing 2 | REPS2 | 1.29866 | 0.00069 |
| NM_015675 | Hs.110571 | growth arrest and DNA-damage-inducible, beta | GADD45B | 1.29866 | 0.00002 |
| BE966922 | Hs.180711 | syntaxin 3 | STX3 | 1.29866 | 0.00002 |
| AL574096 | Hs.438231 | tissue factor pathway inhibitor 2 | TFPI2 | 1.29866 | 0.00002 |
| AB044806 | Hs.647099 | potassium voltage-gated channel, subfamily H (eag-related), member 2 | KCNH2 | 1.29866 | 0.00021 |
| AF098641 | Hs.502328 | CD44 molecule (Indian blood group) | CD44 | 1.29866 | 0.00003 |
| Z24727 | Hs.133892 | tropomyosin 1 (alpha) | TPM1 | 1.29866 | 0.00002 |
| AI922605 | Hs.17441 | collagen, type IV, alpha 1 | COL4A1 | 1.29866 | 0.00039 |
| BG287862 | Hs.502756 | AHNAK nucleoprotein | AHNAK | 1.29866 | 0.00002 |
| AL080170 | Hs.323858 | tripartite motif-containing 58 | TRIM58 | 1.29866 | 0.00021 |
| AK000168 | Hs.694721 | CD24 molecule | CD24 | 1.29866 | 0.00002 |
| AV700298 | Hs.502328 | CD44 molecule (Indian blood group) | CD44 | 1.29866 | 0.00007 |
| NM_018071 | Hs.35125 | hypothetical protein FLJ10357 | FLJ10357 | 1.29866 | 0.00007 |
| BC001875 | Hs.184727 | antigen p97 (melanoma associated) identified by monoclonal antibodies 133.2 and 96.5 | MFI2 | 1.29866 | 0.00021 |
| AK025567 | Hs.655832 | jub, ajuba homolog (Xenopus laevis) | JUB | 1.29866 | 0.00031 |
| BF982002 | Hs.397010 | ribonuclease H2, subunit C | RNASEH2C | 1.29866 | 0.00002 |
| BF221547 | Hs.647971 | phosphodiesterase 5A, cGMP-specific | PDE5A | 1.29866 | 0.00019 |
| BF446390 | Hs.534613 | coiled-coil domain containing 84 | CCDC84 | 1.29866 | 0.00003 |
| N45140 | Hs.596164 | Transcribed locus, strongly similar to XP_001174013.1 PREDICTED: cortactin isoform 1 [Pan troglodytes] | --- | 1.29866 | 0.00002 |
| AI127800 | Hs.474251 | scavenger receptor class F, member 2 | SCARF2 | 1.29866 | 0.00009 |
| AA528070 | Hs.655980 | Transcribed locus | --- | 1.29866 | 0.00035 |
| AA699852 | Hs.503429 | CDNA clone IMAGE:5277380 | --- | 1.29866 | 0.00027 |
| AA971753 | Hs.400698 | Bardet-Biedl syndrome 12 | BBS12 | 1.29866 | 0.00027 |
| AW058617 | Hs.159430 | fibronectin type III domain containing 3B | FNDC3B | 1.29866 | 0.00005 |
| AK021919 | Hs.657345 | leucine rich repeat containing 2 | LRRC2 | 1.29866 | 0.00249 |
| AB046817 | Hs.369520 | synaptotagmin-like 2 | SYTL2 | 1.29866 | 0.00004 |
| AL110175 | Hs.661554 | MRNA; cDNA DKFZp564H0616 (from clone DKFZp564H0616) | --- | 1.29866 | 0.00183 |
| AK025218 | Hs.677339 | CDNA: FLJ21565 fis, clone COL06463 | --- | 1.29866 | 0.00134 |
| AI935586 | Hs.107527 | chromosome 4 open reading frame 19 | C4orf19 | 1.29866 | 0.00027 |
| AA599017 | Hs.159195 | dedicator of cytokinesis 1 | DOCK1 | 1.29866 | 0.00049 |
| AL021977 | Hs.517617 | v-maf musculoaponeurotic fibrosarcoma oncogene homolog F (avian) | MAFF | 1.29866 | 0.00000 |
| BC040322 | Hs.659225 | CDNA clone IMAGE:4828492 | --- | 1.20163 | 0.00077 |
| AA297258 | Hs.482730 | EGF-like repeats and discoidin I-like domains 3 | EDIL3 | 1.20163 | 0.00002 |
| BC039495 | Hs.528540 | hypothetical LOC401074 | LOC401074 | 1.20163 | 0.00062 |
| AL541655 | Hs.444569 | transmembrane protein 49 | TMEM49 | 1.20163 | 0.00002 |
| X16447 | Hs.278573 | CD59 molecule, complement regulatory protein | CD59 | 1.20163 | 0.00002 |
| BF346014 | Hs.460960 | iduronate 2-sulfatase (Hunter syndrome) | IDS | 1.20163 | 0.00005 |
| NM_000202 | Hs.460960 | iduronate 2-sulfatase (Hunter syndrome) | IDS | 1.20163 | 0.00003 |
| NM_021913 | Hs.590970 | AXL receptor tyrosine kinase | AXL | 1.20163 | 0.00039 |
| NM_002081 | Hs.328232 | glypican 1 | GPC1 | 1.20163 | 0.00009 |
| NM_001945 | Hs.799 | heparin-binding EGF-like growth factor | HBEGF | 1.20163 | 0.00108 |
| NM_005756 | Hs.146978 | G protein-coupled receptor 64 | GPR64 | 1.20163 | 0.00009 |
| NM_000366 | Hs.133892 | tropomyosin 1 (alpha) | TPM1 | 1.20163 | 0.00002 |
| NM_003360 | Hs.144197 | UDP glycosyltransferase 8 (UDP-galactose ceramide galactosyltransferase) | UGT8 | 1.20163 | 0.00002 |
| NM_006260 | Hs.656476 | DnaJ (Hsp40) homolog, subfamily C, member 3 | DNAJC3 | 1.20163 | 0.00008 |
| AF179221 | Hs.124147 | F-box and leucine-rich repeat protein 11 | FBXL11 | 1.20163 | 0.00097 |
| U94592 | Hs.80658 | uncoupling protein 2 (mitochondrial, proton carrier) | UCP2 | 1.20163 | 0.00004 |
| J03580 | Hs.591159 | parathyroid hormone-like hormone | PTHLH | 1.20163 | 0.00019 |
| M29277 | Hs.599039 | melanoma cell adhesion molecule | MCAM | 1.20163 | 0.00003 |
| X05610 | Hs.508716 | collagen, type IV, alpha 2 | COL4A2 | 1.20163 | 0.00002 |
| M27487 | Hs.347270 | major histocompatibility complex, class II, DP alpha 1 | HLA-DPA1 | 1.20163 | 0.00011 |
| AI745185 | Hs.503692 | Yes-associated protein 1, 65kDa | YAP1 | 1.20163 | 0.00004 |
| N33167 | Hs.106070 | cyclin-dependent kinase inhibitor 1C (p57, Kip2) | CDKN1C | 1.20163 | 0.00039 |
| BE965369 | Hs.154299 | coagulation factor II (thrombin) receptor-like 1 | F2RL1 | 1.20163 | 0.00002 |
| BF677486 | Hs.56145 | thymosin-like 8 /// thymosin beta15b | MGC39900 /// TMSL8 | 1.20163 | 0.00002 |
| AW188201 | Hs.160556 | acetyl-Coenzyme A carboxylase alpha | ACACA | 1.20163 | 0.00027 |
| NM_018192 | Hs.374191 | leprecan-like 1 | LEPREL1 | 1.20163 | 0.00002 |
| NM_016619 | Hs.546392 | placenta-specific 8 | PLAC8 | 1.20163 | 0.00004 |
| AB020675 | Hs.655684 | contactin associated protein-like 2 | CNTNAP2 | 1.20163 | 0.00021 |
| NM_021785 | Hs.708804 | retinoic acid induced 2 | RAI2 | 1.20163 | 0.00002 |
| NM_017447 | Hs.293811 | chromosome 21 open reading frame 91 | C21orf91 | 1.20163 | 0.00002 |
| AF016005 | Hs.463041 | arginine-glutamic acid dipeptide (RE) repeats | RERE | 1.20163 | 0.00165 |
| AC004010 | Hs.121520 | adhesion molecule with Ig-like domain 2 | AMIGO2 | 1.20163 | 0.00009 |
| AI770166 | Hs.113876 | Wolf-Hirschhorn syndrome candidate 1 | WHSC1 | 1.20163 | 0.00009 |
| BC002490 | Hs.189119 | CXXC finger 5 | CXXC5 | 1.20163 | 0.00003 |
| AF277181 | Hs.709882 | chromosome 1 open reading frame 79 | C1orf79 | 1.20163 | 0.00002 |
| AI446756 | Hs.642877 | metastasis associated lung adenocarcinoma transcript 1 (non-protein coding) | MALAT1 | 1.20163 | 0.00011 |
| AF001540 | Hs.642877 | metastasis associated lung adenocarcinoma transcript 1 (non-protein coding) | MALAT1 | 1.20163 | 0.00003 |
| BE675516 | --- | trophoblast-derived noncoding RNA | TncRNA | 1.20163 | 0.00013 |
| AI091372 | Hs.370950 | AXIN1 up-regulated 1 | AXUD1 | 1.20163 | 0.00024 |
| AI701055 | Hs.436410 | transforming growth factor beta regulator 1 | TBRG1 | 1.20163 | 0.00002 |
| AW190565 | Hs.306814 | lysyl oxidase-like 4 | LOXL4 | 1.20163 | 0.00013 |
| AI832118 | Hs.157726 | CDNA FLJ26252 fis, clone DMC03335 | --- | 1.20163 | 0.00002 |
| AI652872 | Hs.654802 | erythrocyte membrane protein band 4.1 like 5 | EPB41L5 | 1.20163 | 0.00003 |
| AW025602 | Hs.162125 | GLIS family zinc finger 3 | GLIS3 | 1.20163 | 0.00011 |
| AA046424 | Hs.49433 | acyl-CoA thioesterase 4 | ACOT4 | 1.20163 | 0.00004 |
| BF448048 | Hs.510407 | SET domain containing 3 | SETD3 | 1.20163 | 0.00035 |
| H25097 | Hs.431081 | ubiquitin specific peptidase 53 | USP53 | 1.20163 | 0.00004 |
| AB046778 | Hs.503022 | SAPS domain family, member 3 | SAPS3 | 1.20163 | 0.00069 |
| AK024907 | Hs.677290 | CDNA: FLJ21254 fis, clone COL01317 | --- | 1.20163 | 0.00003 |
| AI927919 | Hs.444075 | ubiquitin associated and SH3 domain containing, B | UBASH3B | 1.20163 | 0.00007 |
| AI202235 | Hs.570347 | hypothetical LOC401522 /// hypothetical protein LOC100130623 | LOC100130623 /// RP11-160N1.10 | 1.20163 | 0.00049 |
| AI809536 | Hs.683621 | Transcribed locus | --- | 1.20163 | 0.00087 |
| NM_019022 | Hs.440534 | thioredoxin domain containing 10 | TXNDC10 | 1.09761 | 0.00087 |
| NM_032783 | Hs.659311 | carbonyl reductase 4 | CBR4 | 1.09761 | 0.00225 |
| BC030580 | Hs.178499 | zinc finger and BTB domain containing 44 | ZBTB44 | 1.09761 | 0.00055 |
| BC039079 | Hs.175437 | erythrocyte membrane protein band 4.1 (elliptocytosis 1, RH-linked) | EPB41 | 1.09761 | 0.00108 |
| AK093532 | Hs.683909 | CDNA FLJ36213 fis, clone THYMU2000671 | --- | 1.09761 | 0.00039 |
| CA418310 | Hs.659712 | Transcribed locus | --- | 1.09761 | 0.00044 |
| BC040952 | Hs.175343 | phosphoinositide-3-kinase, class 2, alpha polypeptide | PIK3C2A | 1.09761 | 0.00007 |
| BF983379 | Hs.278573 | CD59 molecule, complement regulatory protein | CD59 | 1.09761 | 0.00002 |
| AI826799 | Hs.76224 | EGF-containing fibulin-like extracellular matrix protein 1 | EFEMP1 | 1.09761 | 0.00002 |
| NM_014822 | Hs.189641 | SEC24 related gene family, member D (S. cerevisiae) | SEC24D | 1.09761 | 0.00024 |
| NM_001982 | Hs.118681 | v-erb-b2 erythroblastic leukemia viral oncogene homolog 3 (avian) | ERBB3 | 1.09761 | 0.00183 |
| NM_001450 | Hs.443687 | four and a half LIM domains 2 | FHL2 | 1.09761 | 0.00002 |
| NM_004357 | Hs.654379 | CD151 molecule (Raph blood group) | CD151 | 1.09761 | 0.00002 |
| NM_016725 | Hs.73769 | folate receptor 1 (adult) | FOLR1 | 1.09761 | 0.00002 |
| NM_018393 | Hs.655341 | t-complex 11 (mouse)-like 1 | TCP11L1 | 1.09761 | 0.00002 |
| NM_002521 | Hs.219140 | natriuretic peptide precursor B | NPPB | 1.09761 | 0.00003 |
| BC005127 | Hs.3416 | adipose differentiation-related protein | ADFP | 1.09761 | 0.00004 |
| AI139569 | Hs.153026 | SWAP-70 protein | SWAP70 | 1.09761 | 0.00007 |
| D43968 | Hs.149261 | runt-related transcription factor 1 (acute myeloid leukemia 1; aml1 oncogene) | RUNX1 | 1.09761 | 0.00002 |
| AF002280 | Hs.85862 | PDZ and LIM domain 3 | PDLIM3 | 1.09761 | 0.00002 |
| AA761181 | Hs.694721 | CD24 molecule | CD24 | 1.09761 | 0.00002 |
| AF096304 | Hs.31130 | transmembrane 7 superfamily member 2 | TM7SF2 | 1.09761 | 0.00203 |
| AF167343 | Hs.478673 | interleukin 1 receptor accessory protein | IL1RAP | 1.09761 | 0.00019 |
| AF220028 | Hs.370515 | tripartite motif-containing 5 | TRIM5 | 1.09761 | 0.0001 |
| M19267 | Hs.133892 | tropomyosin 1 (alpha) | TPM1 | 1.09761 | 0.00002 |
| M28882 | Hs.599039 | melanoma cell adhesion molecule | MCAM | 1.09761 | 0.00009 |
| NM_001845 | Hs.17441 | collagen, type IV, alpha 1 | COL4A1 | 1.09761 | 0.00019 |
| AL575922 | Hs.111779 | secreted protein, acidic, cysteine-rich (osteonectin) | SPARC | 1.09761 | 0.00002 |
| BE138888 | Hs.517601 | ras-related C3 botulinum toxin substrate 2 (rho family, small GTP binding protein Rac2) | RAC2 | 1.09761 | 0.00044 |
| BE222257 | Hs.533712 | RNA binding motif protein 4 | RBM4 | 1.09761 | 0.00002 |
| AU134977 | Hs.648467 | Trophoblast-derived noncoding RNA | TncRNA | 1.09761 | 0.00011 |
| AL049250 | Hs.661773 | hypothetical protein LOC339047 /// hypothetical protein 348162 /// hypothetical LOC642778 /// similar to nuclear pore complex interacting protein | LOC339047 /// LOC348162 /// LOC642778 /// LOC729602 | 1.09761 | 0.00027 |
| AL110298 | Hs.419240 | solute carrier family 2 (facilitated glucose transporter), member 3 /// solute carrier family 2 (facilitated glucose transporter), member 14 | SLC2A14 /// SLC2A3 | 1.09761 | 0.00002 |
| NM_021101 | Hs.439060 | claudin 1 | CLDN1 | 1.09761 | 0.00183 |
| NM_005461 | Hs.712609 | v-maf musculoaponeurotic fibrosarcoma oncogene homolog B (avian) | MAFB | 1.09761 | 0.00002 |
| NM_018302 | Hs.107527 | chromosome 4 open reading frame 19 | C4orf19 | 1.09761 | 0.00035 |
| NM_018413 | Hs.17569 | carbohydrate (chondroitin 4) sulfotransferase 11 | CHST11 | 1.09761 | 0.00008 |
| NM_018190 | Hs.591694 | Bardet-Biedl syndrome 7 | BBS7 | 1.09761 | 0.00006 |
| NM_017654 | Hs.65641 | sterile alpha motif domain containing 9 | SAMD9 | 1.09761 | 0.00035 |
| AI809961 | Hs.507680 | NEDD4 binding protein 2-like 2 | N4BP2L2 | 1.09761 | 0.00002 |
| AF101051 | Hs.439060 | claudin 1 | CLDN1 | 1.09761 | 0.00002 |
| AF220030 | --- | tripartite motif-containing 6 | TRIM6 | 1.09761 | 0.00005 |
| AF312769 | Hs.567542 | cripto, FRL-1, cryptic family 1 /// cripto, FRL-1, cryptic family 1B | CFC1 /// CFC1B | 1.09761 | 0.00134 |
| AA056548 | Hs.709426 | poly (ADP-ribose) polymerase family, member 14 | PARP14 | 1.09761 | 0.00149 |
| BF247906 | Hs.503692 | Yes-associated protein 1, 65kDa | YAP1 | 1.09761 | 0.00003 |
| BF342661 | Hs.368281 | microtubule-associated protein 2 | MAP2 | 1.09761 | 0.00003 |
| AA195074 | Hs.310640 | TRAF-interacting protein with forkhead-associated domain | TIFA | 1.09761 | 0.00002 |
| AU155361 | --- | trophoblast-derived noncoding RNA | TncRNA | 1.09761 | 0.00002 |
| AI244908 | Hs.527973 | suppressor of cytokine signaling 3 | SOCS3 | 1.09761 | 0.00017 |
| BF939317 | Hs.256206 | FLJ41603 protein | FLJ41603 | 1.09761 | 0.00044 |
| AI798680 | --- | --- | --- | 1.09761 | 0.00017 |
| BF435621 | Hs.578433 | FERM domain containing 8 | FRMD8 | 1.09761 | 0.00062 |
| AI041522 | Hs.696735 | Transcribed locus, strongly similar to NP_005768.1 RNA binding motif protein 6 [Homo sapiens] | --- | 1.09761 | 0.00007 |
| AU145950 | Hs.657198 | CDNA FLJ11812 fis, clone HEMBA1006364 | --- | 1.09761 | 0.00039 |
| AI754871 | Hs.709648 | spermatogenesis associated 5 | SPATA5 | 1.09761 | 0.00039 |
| AW070877 | Hs.529677 | Transcribed locus | --- | 1.09761 | 0.00108 |
| BF509573 | Hs.444835 | Transcribed locus | --- | 1.09761 | 0.00062 |
| AW135003 | Hs.709693 | Transcribed locus | --- | 1.09761 | 0.00002 |
| AI554075 | Hs.120204 | Transcribed locus | --- | 1.09761 | 0.00021 |
| AI828075 | Hs.597557 | Transcribed locus | --- | 1.09761 | 0.00002 |
| U73778 | Hs.101302 | collagen, type XII, alpha 1 | COL12A1 | 1.09761 | 0.00009 |
| BE266638 | Hs.406787 | F-box protein 3 | FBXO3 | 1.09761 | 0.00055 |
| AI683802 | Hs.591694 | Bardet-Biedl syndrome 7 | BBS7 | 1.09761 | 0.00004 |
| AA359612 | Hs.591960 | protein associated with topoisomerase II homolog 1 (yeast) | PATL1 | 1.09761 | 0.00108 |
| BF447113 | Hs.656008 | CDNA clone IMAGE:5206119 | --- | 1.09761 | 0.00002 |
| AA354181 | Hs.657806 | KIAA1627 protein | KIAA1627 | 1.09761 | 0.00002 |
| AI421972 | Hs.654666 | nuclear receptor binding SET domain protein 1 | NSD1 | 1.09761 | 0.00039 |
| W02209 | Hs.659999 | Transcribed locus | --- | 1.09761 | 0.00183 |
| BE674055 | Hs.150458 | Transcribed locus | --- | 1.09761 | 0.00010 |
| BF508786 | Hs.613959 | Transcribed locus | --- | 1.09761 | 0.00134 |
| AW975051 | Hs.687674 | Transcribed locus | --- | 1.09761 | 0.00108 |
| W73136 | Hs.49853 | Cell division cycle and apoptosis regulator 1 | CCAR1 | 1.09761 | 0.00008 |
| AL041224 | Hs.655051 | Transcribed locus | --- | 1.09761 | 0.00002 |
| AW292752 | Hs.6483 | oral-facial-digital syndrome 1 | OFD1 | 1.09761 | 0.00019 |
| AI916641 | Hs.191475 | Clone HLS_IMAGE_238756 mRNA sequence | --- | 1.09761 | 0.00003 |
| AA931284 | Hs.599613 | Transcribed locus | --- | 1.09761 | 0.00015 |
| AI521166 | Hs.632156 | CDNA clone IMAGE:4372223 | --- | 1.09761 | 0.00005 |
| AI467945 | Hs.669156 | Transcribed locus | --- | 1.09761 | 0.00002 |
| AA022679 | Hs.529901 | X (inactive)-specific transcript (non-protein coding) | XIST | 1.09761 | 0.00009 |
| AI539783 | Hs.431045 | sperm antigen with calponin homology and coiled-coil domains 1 | SPECC1 | 1.09761 | 0.00002 |
| BC014852 | Hs.591415 | interferon regulatory factor 6 | IRF6 | 1.00000 | 0.00003 |
| AF394735 | Hs.591697 | MAD2 mitotic arrest deficient-like 1 (yeast) | MAD2L1 | 1.00000 | 0.00005 |
| AA960844 | Hs.186579 | family with sequence similarity 111, member B | FAM111B | 1.00000 | 0.00002 |
| NM_001154 | Hs.480653 | annexin A5 | ANXA5 | 1.00000 | 0.00002 |
| BG474541 | Hs.591936 | signal recognition particle receptor ('docking protein') | SRPR | 1.00000 | 0.00031 |
| BG326045 | Hs.171825 | basic helix-loop-helix domain containing, class B, 2 | BHLHB2 | 1.00000 | 0.00003 |
| N24643 | Hs.446017 | WD repeat and SOCS box-containing 1 | WSB1 | 1.00000 | 0.00097 |
| NM_006622 | Hs.398157 | polo-like kinase 2 (Drosophila) | PLK2 | 1.00000 | 0.00002 |
| BE439987 | Hs.462214 | growth arrest-specific 7 | GAS7 | 1.00000 | 0.00021 |
| NM_003113 | Hs.369056 | SP100 nuclear antigen | SP100 | 1.00000 | 0.00002 |
| M65254 | Hs.584790 | protein phosphatase 2 (formerly 2A), regulatory subunit A, beta isoform | PPP2R1B | 1.00000 | 0.00002 |
| NM_002318 | Hs.626637 | lysyl oxidase-like 2 /// ectonucleoside triphosphate diphosphohydrolase 4 | ENTPD4 /// LOXL2 | 1.00000 | 0.00039 |
| NM_004729 | --- | zinc finger, BED-type containing 1 | ZBED1 | 1.00000 | 0.00006 |
| NM_012080 | Hs.185910 | haloacid dehalogenase-like hydrolase domain containing 1A | HDHD1A | 1.00000 | 0.00002 |
| NM_005630 | Hs.518270 | solute carrier organic anion transporter family, member 2A1 | SLCO2A1 | 1.00000 | 0.00003 |
| BG251266 | Hs.283565 | FOS-like antigen 1 | FOSL1 | 1.00000 | 0.00013 |
| NM_005033 | Hs.91728 | exosome component 9 | EXOSC9 | 1.00000 | 0.00004 |
| AI367275 | Hs.471277 | acyl-Coenzyme A dehydrogenase, long chain | ACADL | 1.00000 | 0.00002 |
| NM_014951 | Hs.22653 | zinc finger protein 365 | ZNF365 | 1.00000 | 0.00035 |
| NM_004900 | Hs.226307 | apolipoprotein B mRNA editing enzyme, catalytic polypeptide-like 3B | APOBEC3B | 1.00000 | 0.00003 |
| BC004295 | Hs.437191 | polymerase I and transcript release factor | PTRF | 1.00000 | 0.00004 |
| U34690 | Hs.415067 | coronin, actin binding protein, 1A | CORO1A | 1.00000 | 0.00055 |
| L27624 | Hs.438231 | tissue factor pathway inhibitor 2 | TFPI2 | 1.00000 | 0.00017 |
| BC002538 | Hs.104879 | serpin peptidase inhibitor, clade B (ovalbumin), member 9 | SERPINB9 | 1.00000 | 0.00002 |
| AF001294 | Hs.154036 | pleckstrin homology-like domain, family A, member 2 | PHLDA2 | 1.00000 | 0.00011 |
| AF119889 | Hs.579108 | ADP-ribosylation factor-like 17 pseudogene 1 | ARL17P1 | 1.00000 | 0.00013 |
| AF003114 | Hs.8867 | cysteine-rich, angiogenic inducer, 61 | CYR61 | 1.00000 | 0.00002 |
| U41815 | Hs.524750 | nucleoporin 98kDa | NUP98 | 1.00000 | 0.00002 |
| AI753792 | Hs.502004 | related RAS viral (r-ras) oncogene homolog 2 | RRAS2 | 1.00000 | 0.00002 |
| AL567376 | Hs.706894 | LY6/PLAUR domain containing 1 | LYPD1 | 1.00000 | 0.00002 |
| AI148659 | Hs.477420 | HEG homolog 1 (zebrafish) | HEG1 | 1.00000 | 0.00017 |
| BG475299 | Hs.632133 | cortactin | CTTN | 1.00000 | 0.00031 |
| BF592058 | Hs.175955 | YTH domain containing 1 | YTHDC1 | 1.00000 | 0.00024 |
| AL358975 | Hs.444213 | transducin-like enhancer of split 4 (E(sp1) homolog, Drosophila) | TLE4 | 1.00000 | 0.00039 |
| BF431488 | Hs.591987 | tripartite motif-containing 44 | TRIM44 | 1.00000 | 0.00003 |
| NM_018222 | Hs.607144 | parvin, alpha | PARVA | 1.00000 | 0.00002 |
| NM_016162 | Hs.524210 | inhibitor of growth family, member 4 | ING4 | 1.00000 | 0.00183 |
| NM_022750 | Hs.12646 | poly (ADP-ribose) polymerase family, member 12 | PARP12 | 1.00000 | 0.00006 |
| NM_016445 | Hs.170473 | pleckstrin 2 | PLEK2 | 1.00000 | 0.00015 |
| NM_017515 | Hs.524014 | solute carrier family 35, member F2 | SLC35F2 | 1.00000 | 0.00004 |
| NM_018320 | Hs.368554 | ring finger protein 121 | RNF121 | 1.00000 | 0.00225 |
| NM_014423 | Hs.519313 | AF4/FMR2 family, member 4 | AFF4 | 1.00000 | 0.00019 |
| NM_020645 | Hs.523467 | nuclear receptor interacting protein 3 | NRIP3 | 1.00000 | 0.00002 |
| NM_020179 | Hs.438064 | chromosome 11 open reading frame 75 | C11orf75 | 1.00000 | 0.00002 |
| NM_007083 | Hs.558459 | nudix (nucleoside diphosphate linked moiety X)-type motif 6 | NUDT6 | 1.00000 | 0.00120 |
| AL136935 | Hs.709344 | resistance to inhibitors of cholinesterase 8 homolog A (C. elegans) | RIC8A | 1.00000 | 0.00002 |
| AW246673 | Hs.10784 | family with sequence similarity 46, member A | FAM46A | 1.00000 | 0.00002 |
| AJ012370 | Hs.503560 | N-acetylated alpha-linked acidic dipeptidase 2 | NAALAD2 | 1.00000 | 0.00097 |
| BE549964 | Hs.352588 | chromosome 11 open reading frame 30 | C11orf30 | 1.00000 | 0.00002 |
| AF277187 | Hs.700861 | protein tyrosine phosphatase, mitochondrial 1 | PTPMT1 | 1.00000 | 0.00002 |
| BC005997 | --- | chromosome 1 open reading frame 97 | C1orf97 | 1.00000 | 0.00002 |
| AA557632 | Hs.503692 | Yes-associated protein 1, 65kDa | YAP1 | 1.00000 | 0.00002 |
| AI991160 | Hs.200136 | myosin VB | MYO5B | 1.00000 | 0.00010 |
| AK025444 | Hs.477114 | pleckstrin homology-like domain, family B, member 2 | PHLDB2 | 1.00000 | 0.00002 |
| AI440338 | Hs.709363 | protein tyrosine phosphatase, mitochondrial 1 | PTPMT1 | 1.00000 | 0.00002 |
| AU156710 | Hs.123072 | RAB3B, member RAS oncogene family | RAB3B | 1.00000 | 0.00002 |
| AW519141 | Hs.709762 | Armadillo repeat containing, X-linked 4 | ARMCX4 | 1.00000 | 0.00097 |
| AV687517 | Hs.272011 | Transcribed locus, strongly similar to XP_531236.1 PREDICTED: hypothetical protein [Pan troglodytes] | --- | 1.00000 | 0.00002 |
| N22272 | Hs.144197 | UDP glycosyltransferase 8 (UDP-galactose ceramide galactosyltransferase) | UGT8 | 1.00000 | 0.00002 |
| BE465475 | Hs.130593 | kelch-like 29 (Drosophila) | KLHL29 | 1.00000 | 0.00007 |
| N64025 | Hs.591095 | Protein disulfide isomerase family A, member 3 | PDIA3 | 1.00000 | 0.00019 |
| AW188464 | Hs.431081 | ubiquitin specific peptidase 53 | USP53 | 1.00000 | 0.00002 |
| BF110534 | Hs.480068 | Transcribed locus | --- | 1.00000 | 0.00002 |
| AI222435 | Hs.90250 | CDNA FLJ36413 fis, clone THYMU2010816 | --- | 1.00000 | 0.00183 |
| AF129536 | Hs.464419 | F-box protein 6 | FBXO6 | 1.00000 | 0.00009 |
| AL137725 | Hs.200412 | epiplakin 1 | EPPK1 | 1.00000 | 0.00002 |
| AL137725 | Hs.200412 | epiplakin 1 | EPPK1 | 1.00000 | 0.00003 |
| AA632758 | Hs.663064 | CDNA: FLJ22765 fis, clone KAIA1180 | --- | 1.00000 | 0.00002 |
| N55756 | Hs.85524 | tripartite motif-containing 55 | TRIM55 | 1.00000 | 0.00069 |
| AK021649 | Hs.435771 | apoptosis inhibitor 5 | API5 | 1.00000 | 0.00008 |
| AK001782 | Hs.189119 | CXXC finger 5 | CXXC5 | 1.00000 | 0.00007 |
| AI677701 | Hs.519904 | RNA binding motif protein 24 | RBM24 | 1.00000 | 0.00203 |
| AL577823 | Hs.654868 | Aminopeptidase-like 1 | NPEPL1 | 1.00000 | 0.00024 |
| BF526230 | Hs.8739 | Elongation protein 2 homolog (S. cerevisiae) | ELP2 | 1.00000 | 0.00035 |
| BF676361 | Hs.656309 | Transcribed locus | --- | 1.00000 | 0.00203 |
| BF109910 | Hs.603491 | Transcribed locus | --- | 1.00000 | 0.00134 |
| N76327 | Hs.529618 | transferrin receptor (p90, CD71) | TFRC | 1.00000 | 0.00002 |
| AA481560 | Hs.484195 | chromosome 5 open reading frame 41 | C5orf41 | 1.00000 | 0.00011 |
| AI422414 | Hs.664592 | Transcribed locus | --- | 1.00000 | 0.00024 |
| BF446723 | Hs.644713 | Transcribed locus | --- | 1.00000 | 0.00225 |
| AA931539 | Hs.587092 | MRNA; cDNA DKFZp686P18215 (from clone DKFZp686P18215) | --- | 1.00000 | 0.00024 |
| BF513404 | Hs.678575 | Transcribed locus | --- | 1.00000 | 0.00007 |
| AA782908 | Hs.114777 | hypothetical protein LOC283152 | LOC283152 | 1.00000 | 0.00011 |
| AA707411 | Hs.664843 | Transcribed locus | --- | 1.00000 | 0.00002 |
| AI088104 | Hs.657487 | Transcribed locus | --- | 1.00000 | 0.00021 |
| AW150877 | Hs.127403 | chromosome 11 open reading frame 77 | C11orf77 | 1.00000 | 0.00149 |
| AL525928 | Hs.352588 | chromosome 11 open reading frame 30 | C11orf30 | 1.00000 | 0.00013 |
| AI823453 | Hs.648744 | KIAA1267 | KIAA1267 | 1.00000 | 0.00249 |
| AI763206 | Hs.472838 | serine/threonine kinase 4 | STK4 | 1.00000 | 0.00004 |
| BF063657 | Hs.604381 | Transcribed locus | --- | 1.00000 | 0.00225 |
| AA169554 | Hs.570737 | translocation associated membrane protein 1-like 1 | TRAM1L1 | 1.00000 | 0.00087 |
| L33930 | Hs.694721 | CD24 molecule | CD24 | 1.00000 | 0.00001 |
| AFFX-HUMISGF3A/M97935_MA | Hs.708051 | signal transducer and activator of transcription 1, 91kDa | STAT1 | 1.00000 | 0.00000 |
| NM_145039 | Hs.513832 | hypothetical protein MGC16385 | MGC16385 | -1.00000 | 0.00005 |
| NM_058219 | Hs.461187 | exosome component 6 | EXOSC6 | -1.00000 | 0.00002 |
| AY150851 | Hs.287412 | armadillo repeat containing 10 | ARMC10 | -1.00000 | 0.00077 |
| AI915861 | Hs.623520 | Transcribed locus | --- | -1.00000 | 0.00225 |
| BI715235 | --- | hypothetical gene supported by NM_194304 | LOC439911 | -1.00000 | 0.00087 |
| AA678241 | Hs.558396 | stearoyl-CoA desaturase (delta-9-desaturase) | SCD | -1.00000 | 0.00002 |
| NM_001823 | Hs.173724 | creatine kinase, brain | CKB | -1.00000 | 0.00002 |
| NM_002997 | Hs.224607 | syndecan 1 | SDC1 | -1.00000 | 0.00017 |
| NM_002166 | Hs.180919 | inhibitor of DNA binding 2, dominant negative helix-loop-helix protein | ID2 | -1.00000 | 0.00002 |
| NM_013291 | Hs.493202 | cleavage and polyadenylation specific factor 1, 160kDa | CPSF1 | -1.00000 | 0.00069 |
| NM_004615 | Hs.441664 | tetraspanin 7 | TSPAN7 | -1.00000 | 0.00008 |
| NM_002467 | Hs.202453 | v-myc myelocytomatosis viral oncogene homolog (avian) | MYC | -1.00000 | 0.00003 |
| NM_002485 | Hs.492208 | nibrin | NBN | -1.00000 | 0.00002 |
| NM_000101 | Hs.513803 | cytochrome b-245, alpha polypeptide | CYBA | -1.00000 | 0.00002 |
| NM_014034 | Hs.292316 | ASF1 anti-silencing function 1 homolog A (S. cerevisiae) | ASF1A | -1.00000 | 0.00002 |
| NM_000397 | Hs.292356 | cytochrome b-245, beta polypeptide (chronic granulomatous disease) | CYBB | -1.00000 | 0.00062 |
| U75667 | Hs.708024 | arginase, type II | ARG2 | -1.00000 | 0.00002 |
| NM_000314 | Hs.500466 | phosphatase and tensin homolog (mutated in multiple advanced cancers 1) | PTEN | -1.00000 | 0.00077 |
| NM_001123 | Hs.656586 | adenosine kinase | ADK | -1.00000 | 0.00008 |
| NM_006207 | Hs.458573 | platelet-derived growth factor receptor-like | PDGFRL | -1.00000 | 0.00015 |
| NM_016446 | Hs.493808 | chromosome 9 open reading frame 127 | C9orf127 | -1.00000 | 0.00203 |
| NM_004638 | Hs.436093 | HLA-B associated transcript 2 | BAT2 | -1.00000 | 0.00004 |
| AF288679 | Hs.406534 | high-mobility group 20B | HMG20B | -1.00000 | 0.00017 |
| AI302100 | Hs.93675 | chromosome 10 open reading frame 10 | C10orf10 | -1.00000 | 0.0012 |
| AI951185 | Hs.519445 | Nuclear receptor subfamily 2, group F, member 1 | NR2F1 | -1.00000 | 0.00002 |
| AF286598 | Hs.528051 | angiomotin | AMOT | -1.00000 | 0.00049 |
| BC001811 | Hs.71827 | RRS1 ribosome biogenesis regulator homolog (S. cerevisiae) | RRS1 | -1.00000 | 0.00002 |
| BG054916 | Hs.494538 | patched homolog 1 (Drosophila) | PTCH1 | -1.00000 | 0.00002 |
| AI246769 | Hs.659350 | homeobox A9 | HOXA9 | -1.00000 | 0.00002 |
| M55575 | Hs.654441 | branched chain keto acid dehydrogenase E1, beta polypeptide (maple syrup urine disease) | BCKDHB | -1.00000 | 0.00004 |
| D86586 | Hs.512680 | C-type lectin domain family 11, member A | CLEC11A | -1.00000 | 0.00087 |
| Z25422 | Hs.492333 | serine/threonine kinase 3 (STE20 homolog, yeast) | STK3 | -1.00000 | 0.00004 |
| AA551784 | Hs.371416 | coactivator-associated arginine methyltransferase 1 | CARM1 | -1.00000 | 0.00002 |
| BE613178 | Hs.533013 | cystathionine-beta-synthase | CBS | -1.00000 | 0.00003 |
| U41813 | Hs.659350 | homeobox A9 | HOXA9 | -1.00000 | 0.00002 |
| AL050025 | Hs.210995 | carbonic anhydrase XII | CA12 | -1.00000 | 0.00044 |
| NM_021242 | Hs.522605 | MID1 interacting protein 1 (gastrulation specific G12 homolog (zebrafish)) | MID1IP1 | -1.00000 | 0.00002 |
| NM_016373 | Hs.461453 | WW domain containing oxidoreductase | WWOX | -1.00000 | 0.00049 |
| NM_016593 | Hs.387367 | cytochrome P450, family 39, subfamily A, polypeptide 1 | CYP39A1 | -1.00000 | 0.00003 |
| NM_016202 | Hs.631551 | zinc finger protein 580 | ZNF580 | -1.00000 | 0.0001 |
| NM_021615 | Hs.289092 | coactosin-like 1 (Dictyostelium) | COTL1 | -1.00000 | 0.00002 |
| AL442077 | Hs.705490 | ER lipid raft associated 2 | ERLIN2 | -1.00000 | 0.00002 |
| BC005342 | Hs.282204 | nucleosomal binding protein 1 | NSBP1 | -1.00000 | 0.00005 |
| AI808448 | Hs.221873 | potassium channel tetramerisation domain containing 15 | KCTD15 | -1.00000 | 0.00009 |
| AF245044 | Hs.520341 | 5'-nucleotidase domain containing 1 | NT5DC1 | -1.00000 | 0.00002 |
| AI932310 | Hs.179260 | chromosome 14 open reading frame 4 | C14orf4 | -1.00000 | 0.00002 |
| AK022661 | Hs.709290 | KIAA1967 | KIAA1967 | -1.00000 | 0.00044 |
| AW248770 | Hs.100043 | coiled-coil domain containing 124 | CCDC124 | -1.00000 | 0.00006 |
| AV727346 | Hs.22410 | serine/threonine kinase 11 interacting protein | STK11IP | -1.00000 | 0.00165 |
| BE897866 | Hs.81934 | acyl-Coenzyme A dehydrogenase, short/branched chain | ACADSB | -1.00000 | 0.00002 |
| BE866854 | Hs.631535 | v-akt murine thymoma viral oncogene homolog 2 | AKT2 | -1.00000 | 0.00002 |
| AL036088 | Hs.511265 | sema domain, transmembrane domain (TM), and cytoplasmic domain, (semaphorin) 6D | SEMA6D | -1.00000 | 0.00044 |
| AF141339 | Hs.116935 | zinc finger protein 521 | ZNF521 | -1.00000 | 0.00006 |
| AK002152 | Hs.561815 | staufen, RNA binding protein, homolog 2 (Drosophila) | STAU2 | -1.00000 | 0.00002 |
| AA001400 | Hs.712608 | CDNA clone IMAGE:5311370 | --- | -1.00000 | 0.00031 |
| BF057084 | Hs.513804 | ring finger protein 166 | RNF166 | -1.00000 | 0.00015 |
| BF447286 | Hs.595400 | CDNA FLJ41934 fis, clone PERIC2005111 | --- | -1.00000 | 0.00002 |
| AI652839 | --- | --- | --- | -1.00000 | 0.00249 |
| BE672700 | Hs.491148 | pericentriolar material 1 | PCM1 | -1.00000 | 0.00134 |
| AU152837 | Hs.135787 | sal-like 1 (Drosophila) | SALL1 | -1.00000 | 0.00003 |
| AA057585 | Hs.33519 | CDNA FLJ34311 fis, clone FEBRA2008255 | --- | -1.00000 | 0.00225 |
| BF431989 | Hs.187861 | thyroid hormone receptor, beta (erythroblastic leukemia viral (v-erb-a) oncogene homolog 2, avian) | THRB | -1.00000 | 0.00108 |
| AA429735 | Hs.657197 | chromosome 18 open reading frame 18 | C18orf18 | -1.00000 | 0.00077 |
| AI921002 | Hs.658684 | chromosome 16 open reading frame 46 | C16orf46 | -1.00000 | 0.00055 |
| AI792670 | Hs.586321 | Transcribed locus, moderately similar to XP_001085179.1 PREDICTED: hypothetical protein [Macaca mulatta] | --- | -1.00000 | 0.00017 |
| AA053875 | Hs.491872 | Transcribed locus | --- | -1.00000 | 0.00249 |
| AF113017 | --- | PRO1268 protein | PRO1268 | -1.00000 | 0.00027 |
| AW167727 | Hs.656280 | forkhead box P2 | FOXP2 | -1.00000 | 0.00006 |
| BG166796 | Hs.437366 | Proteasome (prosome, macropain) 26S subunit, ATPase, 2 | PSMC2 | -1.00000 | 0.00006 |
| BF028405 | Hs.653144 | RUN and FYVE domain containing 2 | RUFY2 | -1.00000 | 0.00087 |
| AI921586 | Hs.513490 | aldolase A, fructose-bisphosphate | ALDOA | -1.00000 | 0.00005 |
| BF691045 | Hs.649155 | Transcribed locus, weakly similar to NP_060312.1 hypothetical protein LOC55652 [Homo sapiens] | --- | -1.00000 | 0.00008 |
| AW190593 | Hs.658042 | CDNA FLJ36977 fis, clone BRACE2006344 | --- | -1.00000 | 0.00062 |
| AI686521 | Hs.221737 | lin-7 homolog B (C. elegans) | LIN7B | -1.00000 | 0.00049 |
| AI760495 | Hs.437060 | cytochrome c, somatic | CYCS | -1.00000 | 0.00031 |
| C18318 | Hs.436495 | mex-3 homolog D (C. elegans) | MEX3D | -1.00000 | 0.00012 |
| BC033077 | Hs.710004 | HEAT repeat containing 3 | HEATR3 | -1.09761 | 0.00002 |
| AB052957 | Hs.301989 | stabilin 1 | STAB1 | -1.09761 | 0.00249 |
| R34841 | Hs.702118 | Transcribed locus | --- | -1.09761 | 0.00004 |
| AI635187 | Hs.376071 | cyclin D2 | CCND2 | -1.09761 | 0.00013 |
| U15174 | Hs.144873 | BCL2/adenovirus E1B 19kDa interacting protein 3 | BNIP3 | -1.09761 | 0.00035 |
| NM_002222 | Hs.712541 | inositol 1,4,5-triphosphate receptor, type 1 | ITPR1 | -1.09761 | 0.00007 |
| NM_014789 | Hs.694018 | zinc finger protein 623 | ZNF623 | -1.09761 | 0.00006 |
| AF332197 | Hs.101937 | SIX homeobox 2 | SIX2 | -1.09761 | 0.00062 |
| NM_006080 | Hs.252451 | sema domain, immunoglobulin domain (Ig), short basic domain, secreted, (semaphorin) 3A | SEMA3A | -1.09761 | 0.00087 |
| NM_021057 | Hs.282274 | interferon, alpha 7 | IFNA7 | -1.09761 | 0.00183 |
| AL110191 | Hs.522074 | TSC22 domain family, member 3 | TSC22D3 | -1.09761 | 0.00002 |
| M31523 | Hs.371282 | transcription factor 3 (E2A immunoglobulin enhancer binding factors E12/E47) | TCF3 | -1.09761 | 0.00002 |
| M68874 | Hs.497200 | phospholipase A2, group IVA (cytosolic, calcium-dependent) | PLA2G4A | -1.09761 | 0.00002 |
| M33653 | Hs.695934 | collagen, type XIII, alpha 1 | COL13A1 | -1.09761 | 0.00249 |
| D32039 | Hs.695930 | versican | VCAN | -1.09761 | 0.00002 |
| BC005810 | Hs.512680 | C-type lectin domain family 11, member A | CLEC11A | -1.09761 | 0.00004 |
| AB002363 | Hs.515271 | splicing factor, arginine/serine-rich 14 | SFRS14 | -1.09761 | 0.00003 |
| BE620457 | Hs.131704 | neuropilin 1 | NRP1 | -1.09761 | 0.00002 |
| AI375919 | Hs.592166 | homeobox A10 | HOXA10 | -1.09761 | 0.00011 |
| AI762811 | Hs.465627 | mitogen-activated protein kinase kinase 2 | MAP2K2 | -1.09761 | 0.00017 |
| AL031228 | Hs.415058 | hydroxysteroid (17-beta) dehydrogenase 8 | HSD17B8 | -1.09761 | 0.00077 |
| AB007877 | Hs.654682 | heat shock 70kDa protein 12A | HSPA12A | -1.09761 | 0.00027 |
| AL050035 | Hs.656457 | CDNA FLJ37852 fis, clone BRSSN2014513 | --- | -1.09761 | 0.00011 |
| NM_018052 | Hs.709636 | Vac14 homolog (S. cerevisiae) /// hypothetical protein LOC100130369 | LOC100130369 /// VAC14 | -1.09761 | 0.00006 |
| NM_007197 | Hs.31664 | frizzled homolog 10 (Drosophila) | FZD10 | -1.09761 | 0.00069 |
| AF130089 | Hs.663176 /// Hs.708053 | Transcribed locus | --- | -1.09761 | 0.00035 |
| AL574186 | Hs.557655 | chromosome 19 open reading frame 22 | C19orf22 | -1.09761 | 0.00002 |
| BF197222 | Hs.591375 | chromosome 6 open reading frame 120 | C6orf120 | -1.09761 | 0.00002 |
| W73820 | Hs.221873 | potassium channel tetramerisation domain containing 15 | KCTD15 | -1.09761 | 0.00002 |
| BG290193 | Hs.288042 | zinc finger protein 703 | ZNF703 | -1.09761 | 0.00002 |
| AF277178 | Hs.657061 | SSU72 RNA polymerase II CTD phosphatase homolog (S. cerevisiae) | SSU72 | -1.09761 | 0.00002 |
| AF277178 | Hs.657061 | SSU72 RNA polymerase II CTD phosphatase homolog (S. cerevisiae) | SSU72 | -1.09761 | 0.00006 |
| BC004393 | Hs.111099 | alkB, alkylation repair homolog 7 (E. coli) | ALKBH7 | -1.09761 | 0.00005 |
| AY029179 | Hs.470654 | cell division cycle associated 7 | CDCA7 | -1.09761 | 0.00002 |
| BE747815 | Hs.18949 | EP300 interacting inhibitor of differentiation 2 | EID2 | -1.09761 | 0.00002 |
| BF680438 | Hs.180178 | LON peptidase N-terminal domain and ring finger 1 | LONRF1 | -1.09761 | 0.00002 |
| W55975 | Hs.301431 | zinc finger protein 71 | ZNF71 | -1.09761 | 0.00049 |
| AI199072 | Hs.710673 | similar to RIKEN cDNA C030006K11 gene | MGC70857 | -1.09761 | 0.0012 |
| AW130600 | Hs.99472 | MRNA; cDNA DKFZp564O0862 (from clone DKFZp564O0862) | --- | -1.09761 | 0.00003 |
| AA418816 | Hs.370055 | chromosome 6 open reading frame 117 | C6orf117 | -1.09761 | 0.00055 |
| BE672291 | Hs.63841 | kelch repeat and BTB (POZ) domain containing 7 | KBTBD7 | -1.09761 | 0.00008 |
| AA622837 | --- | homeo box A11, antisense | tcag7.1238 | -1.09761 | 0.00049 |
| N66307 | Hs.253994 | FRAS1 related extracellular matrix protein 2 | FREM2 | -1.09761 | 0.00027 |
| AA843242 | Hs.656581 | basonuclin 2 | BNC2 | -1.09761 | 0.00183 |
| AI452512 | Hs.667007 | Transcribed locus | --- | -1.09761 | 0.00087 |
| AI620209 | Hs.37916 | Dipeptidyl-peptidase 7 | DPP7 | -1.09761 | 0.00002 |
| R33750 | Hs.414467 | Transcribed locus | --- | -1.09761 | 0.00002 |
| BG028463 | Hs.571099 | Transcribed locus | --- | -1.09761 | 0.00003 |
| AI803010 | Hs.554052 | CDNA clone IMAGE:5301910 | --- | -1.09761 | 0.00019 |
| BF513800 | Hs.675919 | potassium voltage-gated channel, KQT-like subfamily, member 5 | KCNQ5 | -1.09761 | 0.00002 |
| AA746320 | Hs.184492 | ELAV (embryonic lethal, abnormal vision, Drosophila)-like 1 (Hu antigen R) | ELAVL1 | -1.09761 | 0.00069 |
| AI655903 | Hs.274136 | G protein-coupled receptor 124 | GPR124 | -1.09761 | 0.00152 |
| BC009278 | --- | hypothetical LOC645722 | LOC645722 | -1.20163 | 0.00002 |
| CA448125 | Hs.445239 | CDNA FLJ12777 fis, clone NT2RP2001720 | --- | -1.20163 | 0.00002 |
| BF692729 | Hs.461187 | exosome component 6 | EXOSC6 | -1.20163 | 0.00004 |
| AW827204 | Hs.658350 | CDNA clone IMAGE:5277883 | --- | -1.20163 | 0.00049 |
| NM_001759 | Hs.376071 | cyclin D2 | CCND2 | -1.20163 | 0.00002 |
| D13891 | Hs.180919 | inhibitor of DNA binding 2, dominant negative helix-loop-helix protein | ID2 | -1.20163 | 0.00008 |
| NM_002923 | Hs.78944 | regulator of G-protein signaling 2, 24kDa | RGS2 | -1.20163 | 0.00004 |
| NM_007361 | Hs.369840 | nidogen 2 (osteonidogen) | NID2 | -1.20163 | 0.00002 |
| NM_006823 | Hs.433700 | protein kinase (cAMP-dependent, catalytic) inhibitor alpha | PKIA | -1.20163 | 0.00002 |
| NM_012338 | Hs.16529 | tetraspanin 12 | TSPAN12 | -1.20163 | 0.00002 |
| NM_030781 | Hs.464422 | collectin sub-family member 12 | COLEC12 | -1.20163 | 0.00002 |
| U15642 | Hs.445758 | E2F transcription factor 5, p130-binding | E2F5 | -1.20163 | 0.00008 |
| BF218922 | Hs.695930 | versican | VCAN | -1.20163 | 0.00002 |
| BF431618 | Hs.591375 | chromosome 6 open reading frame 120 | C6orf120 | -1.20163 | 0.00002 |
| AB040897 | Hs.368569 | RAN binding protein 10 | RANBP10 | -1.20163 | 0.00049 |
| BC005085 | Hs.334518 | zinc finger protein 607 | ZNF607 | -1.20163 | 0.00027 |
| AK000680 | Hs.266175 | phosphoprotein associated with glycosphingolipid microdomains 1 | PAG1 | -1.20163 | 0.00004 |
| AW592604 | Hs.528574 | topoisomerase (DNA) I, mitochondrial | TOP1MT | -1.20163 | 0.00044 |
| BF593636 | Hs.445239 | CDNA FLJ12777 fis, clone NT2RP2001720 | --- | -1.20163 | 0.00002 |
| AI202201 | Hs.657367 | junctophilin 1 | JPH1 | -1.20163 | 0.00004 |
| BE464337 | Hs.86045 | family with sequence similarity 120C | FAM120C | -1.20163 | 0.00004 |
| AI939363 | Hs.554050 | CDNA FLJ43322 fis, clone NT2RI2027975 | --- | -1.20163 | 0.00002 |
| BF185922 | Hs.479954 | methylenetetrahydrofolate dehydrogenase (NADP+ dependent) 2-like | MTHFD2L | -1.20163 | 0.00225 |
| AI612775 | Hs.145710 | Transcribed locus | --- | -1.20163 | 0.00039 |
| AI916560 | Hs.662386 | Transcribed locus | --- | -1.20163 | 0.00149 |
| BG484193 | Hs.199487 | RAS-like, estrogen-regulated, growth inhibitor | RERG | -1.20163 | 0.00007 |
| AK054643 | Hs.335293 | sterile alpha motif domain containing 11 | SAMD11 | -1.29866 | 0.0001 |
| AL832136 | Hs.407141 | Transcribed locus | --- | -1.29866 | 0.00002 |
| NM_004052 | Hs.144873 | BCL2/adenovirus E1B 19kDa interacting protein 3 | BNIP3 | -1.29866 | 0.00002 |
| NM_002290 | Hs.654572 | laminin, alpha 4 | LAMA4 | -1.29866 | 0.00002 |
| BF590263 | Hs.695930 | versican | VCAN | -1.29866 | 0.00002 |
| NM_004385 | Hs.695930 | versican | VCAN | -1.29866 | 0.00002 |
| NM_000735 | Hs.119689 | glycoprotein hormones, alpha polypeptide | CGA | -1.29866 | 0.00002 |
| L35594 | Hs.190977 | ectonucleotide pyrophosphatase/phosphodiesterase 2 (autotaxin) | ENPP2 | -1.29866 | 0.00002 |
| U79297 | Hs.530199 | ankyrin repeat domain 46 | ANKRD46 | -1.29866 | 0.00002 |
| AL137534 | Hs.56876 | hypothetical protein DKFZp434H1419 | DKFZp434H1419 | -1.29866 | 0.00225 |
| NM_016579 | Hs.558499 | CD320 molecule | CD320 | -1.29866 | 0.00002 |
| AA888858 | Hs.445711 | Transcribed locus | --- | -1.29866 | 0.00013 |
| AI218850 | Hs.467151 | Josephin domain containing 2 | JOSD2 | -1.29866 | 0.00002 |
| BE644809 | Hs.479439 | protocadherin 7 | PCDH7 | -1.29866 | 0.00002 |
| R42166 | Hs.298705 | contactin 4 | CNTN4 | -1.29866 | 0.00013 |
| AW005298 | Hs.143408 | CDNA clone IMAGE:30332316 | --- | -1.29866 | 0.00003 |
| AI262277 | Hs.632559 | hypothetical LOC646903 | LOC646903 | -1.29866 | 0.00134 |
| AA706818 | Hs.119878 | mixed lineage kinase domain-like | MLKL | -1.29866 | 0.0001 |
| AI091533 | Hs.135167 | acidic repeat containing | ACRC | -1.29866 | 0.00013 |
| AI421796 | Hs.132591 | solute carrier family 10 (sodium/bile acid cotransporter family), member 4 | SLC10A4 | -1.29866 | 0.00002 |
| AW052186 | Hs.547771 | Transcribed locus | --- | -1.29866 | 0.00134 |
| R49102 | Hs.709289 | Hypothetical protein LOC100130837 | LOC100130837 | -1.29866 | 0.00011 |
| AW291402 | Hs.656280 | forkhead box P2 | FOXP2 | -1.29866 | 0.00002 |
| NM_005345 | Hs.520028 | heat shock 70kDa protein 1A /// heat shock 70kDa protein 1B | HSPA1A /// HSPA1B | -1.40054 | 0.00002 |
| NM_005346 | Hs.520028 | heat shock 70kDa protein 1A /// heat shock 70kDa protein 1B | HSPA1A /// HSPA1B | -1.40054 | 0.00002 |
| NM_000540 | Hs.466664 | ryanodine receptor 1 (skeletal) | RYR1 | -1.40054 | 0.00165 |
| AW301641 | Hs.369017 | RAB2A, member RAS oncogene family | RAB2A | -1.40054 | 0.00055 |
| BF449063 | Hs.409662 | collagen, type XIV, alpha 1 | COL14A1 | -1.40054 | 0.00006 |
| AB018322 | Hs.477547 | transmembrane and coiled-coil domain family 1 | TMCC1 | -1.40054 | 0.00035 |
| AL137749 | Hs.405873 | myo-inositol 1-phosphate synthase A1 | ISYNA1 | -1.40054 | 0.00002 |
| AI936523 | Hs.556754 | hypothetical protein LOC27320 /// trinucleotide repeat containing 18 | LOC27320 /// TNRC18 | -1.40054 | 0.00002 |
| AA603467 | Hs.195710 | zinc finger protein 503 | ZNF503 | -1.40054 | 0.00003 |
| AI215106 | Hs.705877 | Transcribed locus, strongly similar to NP_058767.1 insulin receptor [Rattus norvegicus] | --- | -1.40054 | 0.00002 |
| AI650353 | Hs.129452 | dachshund homolog 1 (Drosophila) | DACH1 | -1.40054 | 0.00009 |
| BF114646 | Hs.592572 | CDNA clone IMAGE:5263177 | --- | -1.40054 | 0.00002 |
| AW952781 | Hs.708094 | hypothetical protein LOC100128822 | LOC100128822 | -1.40054 | 0.00002 |
| BG400596 | Hs.193172 | hypothetical protein LOC202781 | LOC202781 | -1.40054 | 0.00009 |
| AI758317 | Hs.124384 | Transcribed locus | --- | -1.40054 | 0.00031 |
| NM_173495 | Hs.319503 | patched domain containing 1 | PTCHD1 | -1.5008 | 0.00002 |
| BG704082 | Hs.550108 | CDNA clone IMAGE:5261375 | --- | -1.5008 | 0.00021 |
| BC024246 | Hs.156928 | CDNA clone IMAGE:5288145 | --- | -1.5008 | 0.00013 |
| NM_003748 | Hs.77448 | aldehyde dehydrogenase 4 family, member A1 | ALDH4A1 | -1.5008 | 0.00027 |
| NM_003662 | Hs.495728 | pirin (iron-binding nuclear protein) | PIR | -1.5008 | 0.00002 |
| NM_001965 | Hs.3052 | early growth response 4 | EGR4 | -1.5008 | 0.00024 |
| NM_014432 | Hs.445868 | interleukin 20 receptor, alpha | IL20RA | -1.5008 | 0.00134 |
| AJ249900 | Hs.497349 | SPARC related modular calcium binding 1 | SMOC1 | -1.5008 | 0.00002 |
| AI417268 | Hs.355559 | hypothetical LOC550643 | LOC550643 | -1.5008 | 0.00004 |
| BF593856 | Hs.437060 | cytochrome c, somatic | CYCS | -1.5008 | 0.00069 |
| N34402 | Hs.27996 | Transcribed locus | --- | -1.5008 | 0.00009 |
| BM978026 | Hs.588994 | hypothetical LOC284356 | LOC284356 | -1.59932 | 0.00019 |
| NM_000633 | Hs.150749 | B-cell CLL/lymphoma 2 | BCL2 | -1.59932 | 0.00062 |
| NM_005052 | Hs.45002 | ras-related C3 botulinum toxin substrate 3 (rho family, small GTP binding protein Rac3) | RAC3 | -1.59932 | 0.00055 |
| M58026 | Hs.239600 | calmodulin-like 3 | CALML3 | -1.59932 | 0.0001 |
| AW007532 | Hs.635441 | insulin-like growth factor binding protein 5 | IGFBP5 | -1.59932 | 0.00004 |
| R94644 | Hs.695930 | versican | VCAN | -1.59932 | 0.00002 |
| BF245954 | Hs.433700 | Protein kinase (cAMP-dependent, catalytic) inhibitor alpha | PKIA | -1.59932 | 0.00005 |
| AA479290 | Hs.105575 | MRNA; cDNA DKFZp686C1384 (from clone DKFZp686C1384) | --- | -1.59932 | 0.00009 |
| D59759 | --- | --- | --- | -1.59932 | 0.00004 |
| H78106 | Hs.446671 | Transcribed locus, strongly similar to NP_001027392.1 syntaxin binding protein 1 isoform b [Homo sapiens] | --- | -1.59932 | 0.00002 |
| AI862542 | Hs.363526 | CDNA clone IMAGE:4837650 | --- | -1.59932 | 0.00009 |
| H46176 | Hs.573319 | Transcribed locus | --- | -1.59932 | 0.00069 |
| NM_014705 | Hs.654652 | dedicator of cytokinesis 4 | DOCK4 | -1.70044 | 0.00002 |
| NM_003604 | Hs.592215 | insulin receptor substrate 4 | IRS4 | -1.70044 | 0.00002 |
| BC004241 | Hs.654572 | laminin, alpha 4 | LAMA4 | -1.70044 | 0.00011 |
| NM_017990 | Hs.655245 | pyruvate dehydrogenase phosphatase regulatory subunit | PDPR | -1.70044 | 0.00225 |
| BG289306 | Hs.592172 | homeobox A13 | HOXA13 | -1.70044 | 0.00044 |
| BF445865 | --- | --- | --- | -1.70044 | 0.00039 |
| AI827473 | --- | --- | --- | -1.70044 | 0.00015 |
| AFFX-HUMRGE/M10098_3 | --- | --- | --- | -1.70044 | 0.00000 |
| NM_017797 | Hs.465543 | BTB (POZ) domain containing 2 | BTBD2 | -1.79909 | 0.00049 |
| AU145890 | Hs.348883 | forkhead box C1 | FOXC1 | -1.79909 | 0.00002 |
| BG532690 | Hs.694732 | integrin, alpha 4 (antigen CD49D, alpha 4 subunit of VLA-4 receptor) | ITGA4 | -1.79909 | 0.00005 |
| BF590528 | --- | similar to hCG2042068 | LOC100130740 | -1.79909 | 0.00009 |
| BE222279 | Hs.593430 | mindbomb homolog 2 (Drosophila) | MIB2 | -1.89918 | 0.00044 |
| AFFX-HUMRGE/M10098_M | --- | --- | --- | -1.89918 | 0.00000 |
| NM_030820 | Hs.47629 | collagen, type XXI, alpha 1 | COL21A1 | -2.00000 | 0.00069 |
| AK024456 | Hs.509664 | FYVE, RhoGEF and PH domain containing 2 | FGD2 | -2.00000 | 0.00165 |
| BF589359 | Hs.266175 | phosphoprotein associated with glycosphingolipid microdomains 1 | PAG1 | -2.10098 | 0.00008 |
| NM_001453 | Hs.348883 | forkhead box C1 | FOXC1 | -2.19849 | 0.00002 |
| AA416756 | Hs.681030 | Transcribed locus | --- | -2.19849 | 0.00165 |
| AI927000 | Hs.648106 | sclerostin domain containing 1 | SOSTDC1 | -2.29866 | 0.00011 |
| AA001390 | --- | --- | --- | -2.29866 | 0.00183 |
| AFFX-HUMRGE/M10098_5 | --- | --- | --- | -2.40054 | 0.00000 |
| AI379517 | Hs.375142 | ral guanine nucleotide dissociation stimulator-like 3 | RGL3 | -2.50080 | 0.00183 |
| AI864183 | Hs.110069 | family with sequence similarity 133, member A | FAM133A | -2.50080 | 0.00134 |
| AA148535 | Hs.319503 | patched domain containing 1 | PTCHD1 | -2.59932 | 0.00006 |
| NM_016354 | Hs.235782 | solute carrier organic anion transporter family, member 4A1 /// similar to Solute carrier organic anion transporter family, member 4A1 | LOC100134295 /// SLCO4A1 | -2.79909 | 0.00225 |
| AI016316 | Hs.491292 | CDNA FLJ36559 fis, clone TRACH2009291 | --- | -2.79909 | 0.00039 |
| AI051127 | Hs.132584 | phosphodiesterase 4C, cAMP-specific (phosphodiesterase E1 dunce homolog, Drosophila) | PDE4C | -2.89918 | 0.00021 |
| AB033100 | Hs.202351 | KIAA1274 | KIAA1274 | -3.00000 | 0.00009 |
| AA461190 | Hs.369479 | CDNA FLJ26260 fis, clone DMC05193 | --- | -3.09930 | 0.00120 |
| BE855713 | Hs.23133 | hypothetical protein MGC9913 | MGC9913 | -3.20006 | 0.00007 |
| X95654 | Hs.112743 | synaptonemal complex protein 1 | SYCP1 | -3.40054 | 0.00225 |
| AI802969 | Hs.633921 | Transcribed locus | --- | -3.60051 | 0.00062 |
| AW474158 | Hs.662043 | zinc finger protein 528 | ZNF528 | -3.70044 | 0.00069 |
| AW135556 | Hs.669747 | Transcribed locus | --- | -3.70044 | 0.00134 |
| BC017743 | Hs.680651 | Homo sapiens, clone IMAGE:4391558, mRNA | --- | -3.80012 | 0.00249 |
| AI936034 | Hs.72981 | Transcribed locus | --- | -3.90014 | 0.00015 |
| BC038548 | Hs.385793 | Homo sapiens, clone IMAGE:5242593, mRNA | --- | -4.10014 | 0.00069 |
| BC000568 | Hs.191616 | transmembrane protein 108 | TMEM108 | -4.20006 | 0.00165 |
| AI264121 | Hs.658134 | plexin domain containing 2 | PLXDC2 | -4.20006 | 0.00013 |
| BC035349 | --- | --- | --- | -4.39985 | 0.00134 |
| N22776 | Hs.556072 | Transcribed locus | --- | -4.39985 | 0.00039 |
| AW611685 | Hs.372303 | CDNA FLJ45450 fis, clone BRSTN2002691 | --- | -4.50016 | 0.00004 |
| BC041865 | Hs.616780 | CDNA clone IMAGE:5271374 | --- | -4.59991 | 0.0012 |
| AF232217 | Hs.545395 | Pregnancy-induced hypertension syndrome-related protein (PIH2) | --- | -4.80012 | 0.00062 |
| BC020814 | Hs.442781 | SGT1, suppressor of G2 allele of SKP1 like 1 (S. cerevisiae) /// similar to SGT1, suppressor of G2 allele of SKP1 like 1 (S. cerevisiae) | LOC100130423 /// SUGT1L1 | -5.49985 | 0.00225 |
