# Supplementary figures and images for: Correction: Interaction of G-Protein βγ Complex with Chromatin Modulates GPCR-Dependent Gene Regulation
Source: PLoS One. 2016 May 4;11(5):e0155198. doi: 10.1371/journal.pone.0155198 (PMC4856381; doi:10.1371/journal.pone.0155198)

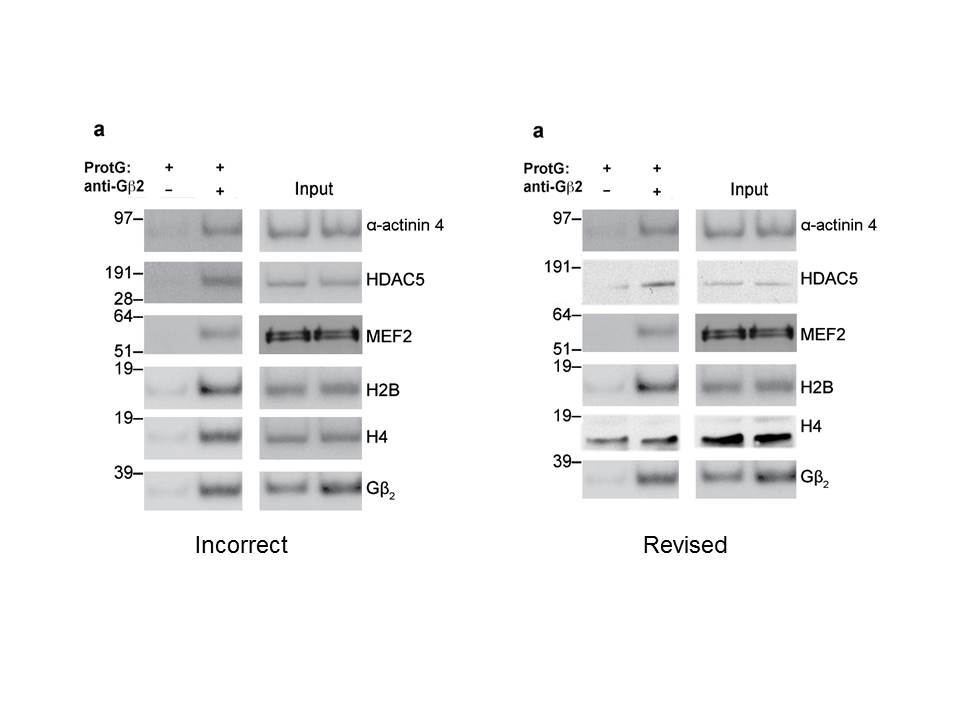

Supplement: S1 Fig — (TIF) [file pone.0155198.s001.tif]
